# Selective uni- and bidirectional homologation of diborylmethane†
†Electronic supplementary information (ESI) available: General procedures, synthetic details and NMR spectra. See DOI: 10.1039/c6sc05338f



**DOI:** 10.1039/c6sc05338f

**Published:** 2017-02-09

**Authors:** Daniel J. Blair, Damiano Tanini, Joseph M. Bateman, Helen K. Scott, Eddie L. Myers, Varinder K. Aggarwal

**Affiliations:** a School of Chemistry , University of Bristol , Cantock's Close , Bristol , BS8 1TS , UK . Email: V.Aggarwal@bristol.ac.uk

## Abstract

Diborylmethane can be homologated uni- and bidirectionally by using enantiomerically pure lithium-stabilized carbenoids to give 1,2- and 1,3-bis(boronic esters), respectively, in good yield and with excellent levels of enantio- and diastereoselectivity. The high sensitivity of the transformation to steric hindrance enables the exclusive operation of either manifold, effected through the judicious choice of the type of carbenoid, which can be a sparteine-ligated or a diamine-free lithiated benzoate/carbamate. The scope of the 1,2-bis(boronic esters) so generated is complementary to that encompassed by the asymmetric diboration of alkenes, in that primary–secondary and primary–tertiary 1,2-bis(boronic esters) can be prepared with equally high levels of selectivity and that functional groups, such as terminal alkynes and alkenes, are tolerated. Methods for forming *C*_2_-symmetric and non-symmetrical *anti* and *syn* 1,3-bis(boronic esters) are also described and represent a powerful route towards 1,3-functionalized synthetic intermediates.

## Abstract 2

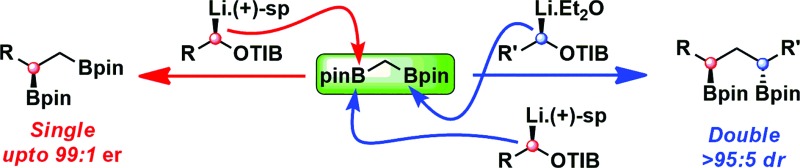
Diborylmethane can be homologated uni- and bidirectionally by using enantiomerically pure lithium-stabilized carbenoids to give 1,2- and 1,3-bis(boronic esters).

## Introduction

**Fig. 1:**
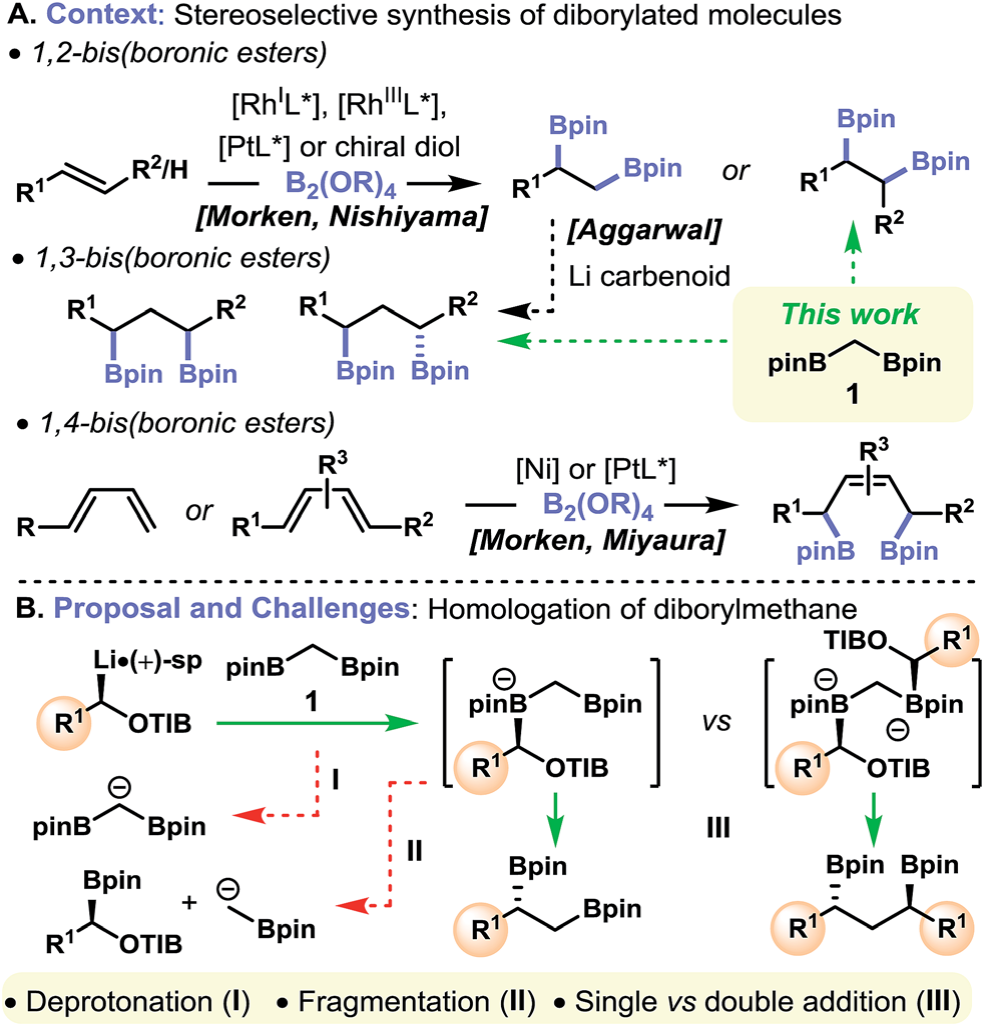
Context, proposal and challenges.

Investigations into the utility of organic molecules containing multiple C-sp^3^ boryl units continue to flourish, enabled in part by recent developments in the asymmetric diboration of alkenes.^1^ It is now clear that polyboronic esters are powerful intermediates in organic synthesis owing to the abundance of functional groups in which the C–B bonds can be transformed.^2^ Many of these methods are stereospecific and regioselective transformation of polyboronic esters is feasible through the judicious manipulation of steric effects^3^ and proximal functional groups.^4^ Despite these advances, the types of polyboronic esters available for further functionalization remain limited. For example, although primary–secondary and a very limited number of secondary–secondary 1,2-bis(boronic) esters are readily available with high levels of enantiopurity, obtained through the platinum-^5^ or rhodium-mediated^6^ diboration of the corresponding alkenes or the hydroxyl-directed diboration^7^ of enantioenriched alkenyl alcohols (Fig. 1A), 1,2-bis(boronic) esters with higher levels of substitution are not.^8^ Furthermore, metal-catalyzed diboration reactions are not tolerant of several functional groups, for example alkynes, thus further limiting the diversity of polyboronic esters available. Herein, we present an alternative method for preparing 1,2-bis(boronic esters)—the one-carbon homologation of diborylmethane **1**. Specifically, when treated with enantioenriched lithiated benzoates or carbamates, diborylmethane **1** undergoes stereospecific homologation to give primary–secondary and primary–tertiary 1,2-bis(boronic esters). Furthermore, we show that diborylmethane can be homologated in both directions in a one-pot process to give secondary–secondary 1,3-bis(boronic esters), a class of polyboronic esters with similar potential for application to their 1,2-related counterparts.^9^

We have recently developed efficient methodology for the homologation of boronic esters by using stereodefined lithiated carbamates^10^ and 2,4,6-triisopropylbenzoates^11^ (TIB esters). The transformation involves the stereoretentive complexation of the lithiated carbamate^12^/TIB ester^13^ with the boronic ester to form a boronate complex, which then undergoes a stereoinvertive 1,2-metallate rearrangement^14^ to generate a homologated boronic ester. Our attention was drawn to commercially available diborylmethane **1**,^15^ which we envisioned serving as a valuable reagent for the elaboration of stereodefined carbenoids (Fig. 1B). Specifically, the complexation of one boryl moiety of diborylmethane **1** with a carbenoid followed by 1,2-metallate rearrangement would give a 1,2-bis(boronic ester). In a second manifold, the complexation of both boryl groups to the same or different carbenoids, thus forming a dianionic 1,1-bis(boronate), with subsequent rearrangement at both centers, would give a 1,3-bis(boronic ester), thus representing a three-component coupling with diborylmethane **1** acting as a linchpin. For these transformations to be successful, a number of key criteria would have to be met: (i) the acidic methylene group of diborylmethane **1** must not undergo deprotonation (**I**);^16^ (ii) fragmentation of the intermediate boronate complex to form stabilized α-boryl carbanions must not occur (**II**);^15*a*^ (iii) single *versus* double homologation must be controlled (**III**) (Fig. 1B).

## Results and discussion

**Fig. 2:**
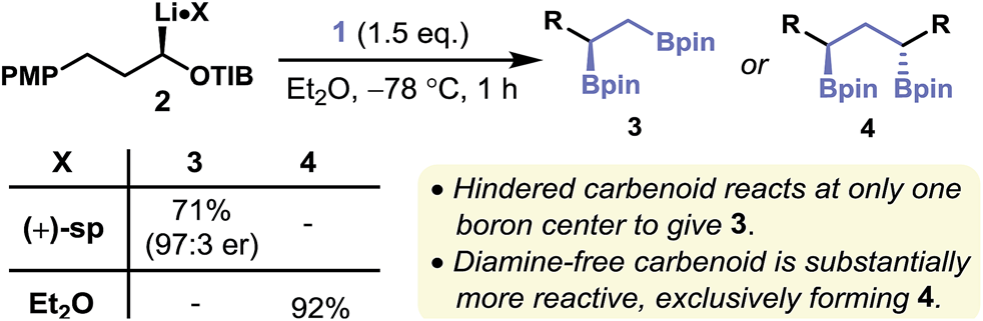
Key findings.

(+)-Sparteine-ligated lithiated TIB ester **2** ^17^ met all three of the criteria outlined above: by generating this carbenoid through the sparteine-mediated deprotonation of the corresponding benzoate and then treatment with diborylmethane **1**, the single-homologation product **3** was isolated in high yield and with very high levels of enantiopurity (Fig. 2). Surprisingly, when using the corresponding diamine-free carbenoid (generated by tin–lithium exchange of the corresponding enantioenriched α-stannyl benzoate) the double-addition product **4** was obtained exclusively; none of the monoaddition product **3** was observed.^18^ In contrast, Shibata and co-workers have shown that the diamine-free Matteson-type carbenoid (LiCH_2_Cl), which was generated *in situ*, reacts with the more sterically hindered 1,1-diborylphenylethane to give the single-homologation product.^19,20^ Clearly the level of homologation of geminal diboryl compounds is very sensitive to the nature of the carbenoid employed.

**Fig. 3:**
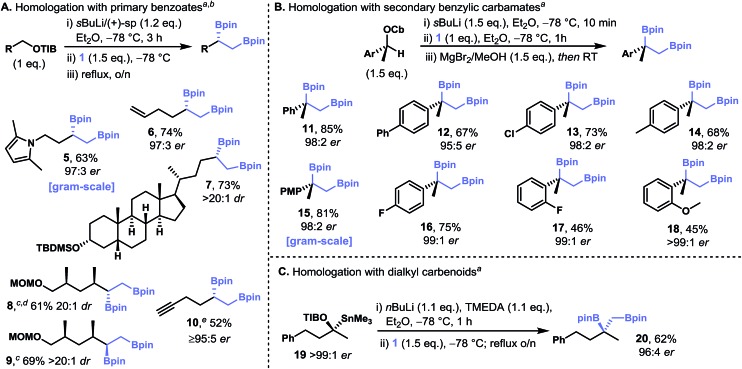
Stereoselective synthesis of 1,2-bis(boronic esters) from primary benzoates (A), secondary benzylic carbamates (B) and secondary dialkyl benzoates (C). Footnotes: (a) enantiomeric ratios determined by HPLC, GC or SFC analysis using chiral stationary phases. (b) Diastereomeric ratios determined by ^1^H NMR analysis. (c) Prepared from the corresponding carbamate (d) using (–)-sparteine in place of (+)-sparteine. (e) 2.2 equivalents of (+)-sparteine, *s*BuLi and **1**. Abbreviations: Cb = *N*,*N*-diisopropyl carbamoyl, MOM = methoxymethyl, PMP = *para*-methoxyphenyl, TBS = *tert*-butyldimethylsilyl, TIB = 2,4,6-triisopropylbenzoyl, TMEDA = *N*,*N*,*N*′,*N*′-tetramethylethylenediamine, pin = pinacolato.

Having established the optimal conditions for single and double homologation we set out to demonstrate the generality of our protocol for the synthesis of chiral non-racemic 1,2-bis(boronic esters). The homologation of diborylmethane **1** with lithiated primary benzoates containing a pyrrole-masked primary amine,^21^ a terminal olefin, and a steroidal group gave the corresponding 1,2-bis(boronic esters), **5–7**, in high yield and enantiopurity (Fig. 3A). Additionally, the 1,2-bis(boronic ester) **5** was prepared on gram scale with comparable yields and selectivity. In contrast to metal-mediated diboration, the stereoselectivity of this homologative process appears to be insensitive to proximal stereogenic centers as epimeric 1,2-bis(boronic esters) **8** and **9** were prepared with similarly high levels of diastereoselectivity. The potential adverse effect of acidic functional groups on the carbenoid partner could be mitigated by their *in situ* protection as the corresponding conjugate bases by using excess *s*-BuLi. This protocol facilitated the diborylative transformation of a carbenoid bearing a terminal alkyne (to give **10**). Again, this particular transformation is significant in that alkyne groups are incompatible with alkene diboration reactions.

Based on our observation that sterically hindered diamine-ligated primary carbenoids are highly selective for the single homologation of **1**, we reasoned that secondary benzylic carbenoids, which can be generated through the stereospecific deprotonation of the corresponding carbamates in the absence of a diamine, should be similarly selective. We were also mindful that the products, primary–tertiary 1,2-bis(boronic esters), are not accessible in useful yields or levels of enantiopurity through the asymmetric diboration of 1,1-disubstituted alkenes, thus giving such a process extra significance. Pleasingly, the treatment of enantiopure (*S*)-1-phenylethanol-derived lithiated carbamate with diborylmethane **1** gave primary–tertiary 1,2-bis(boronic ester) **11** in high yield and with a high level of enantiopurity (98 : 2 e.r.; Fig. 3B). The addition of a Lewis acid (MgBr_2_) and MeOH at –78 °C immediately after the formation of the boronate complex, yet prior to warming to room temperature (the 1,2-metallate rearrangement only occurs at elevated temperatures), was essential for achieving high yields and high levels of enantiopurity.^10*c*^

These homologation reactions were insensitive to the electronic demands of the aromatic ring as *p*-Ph, *p*-Cl, *p*-Me, *p*-MeO and *p*-F substituted carbamates gave the desired 1,2-bis(boronic esters) **12–16** in high yields and high levels of enantioselectivity. These transformations were similarly effective on gram scale (**15**). Although the transformation of *ortho*-substituted benzylic carbamates (*o*-F and *o*-MeO: **17** and **18**) gave more moderate yields of the products, the levels of enantiopurity remained very high. The transformation of secondary alkyl benzoates (*i.e.* non-benzylic)^22^ necessitated the generation of the chiral carbenoid from the corresponding stannane through tin–lithium exchange. The carbenoid so generated from **19** (>99 : 1 e.r.) reacted with diborylmethane **1** to give the corresponding 1,2-bis(boronic ester) **20** in 62% yield and with high levels of enantiospecificity (96 : 4 e.r.), the slight erosion of stereochemical information being most likely due to competing stereoinversion of the carbanion during the formation of the boronate complex.^22,23^

**Fig. 4:**
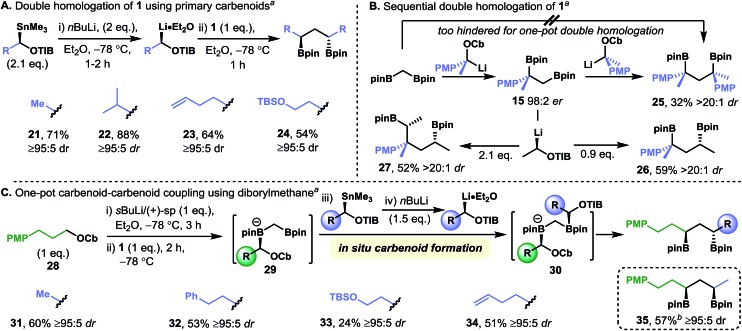
Synthesis of *C*_2_-symmetric-1,3-bis(boronic esters) from diborylmethane **1** by using diamine-free primary carbenoids (A) and stepwise double homologation with secondary benzylic carbamates (B). Also, the one-pot mixed homologation of diborylmethane to form non-symmetric 1,3-bis(boronic esters) (C). Footnotes: (a) diastereomeric ratios were determined by ^1^H NMR analysis. (b) Opposite enantiomer of stannane was used. Abbreviations: Cb = *N*,*N*-diisopropyl carbamoyl, PMP = *para*-methoxyphenyl, TBS = *tert*-butyldimethylsilyl, TIB = 2,4,6-triisopropylbenzoyl, pin = pinacolato.

Our initial studies established that diamine-free primary lithiated benzoates react with diborylmethane **1** to give *C*_2_-symmetric secondary–secondary 1,3-bis(boronic esters), that is, both ends of the diborylmethane undergo homologation (Fig. 2). We explored the scope of this transformation by using a range of diamine-free lithiated benzoates, which were generated through tin–lithium exchange of the corresponding enantiopure α-stannyl benzoates (Fig. 4A). The double-homologation reactions of diborylmethane **1** with primary lithiated benzoates gave a range of *C*_2_-symmetric secondary–secondary 1,3-bis(boronic esters) (**21–24**) in high yield and near-perfect diastereoselectivity. In contrast, one-pot double homologation with the more hindered secondary benzylic lithiated carbamates proved more challenging: a low yield of *C*_2_-symmetric tertiary–tertiary 1,3-bis(boronic ester) **25** was obtained, even when using a large excess of the requisite lithiated carbamate (Fig. 4B). However, we overcame this limitation through the isolation of the mono-homologation product, **15**, and resubjecting it to the homologation conditions, a protocol that gave tertiary–tertiary 1,3-bis(boronic ester) **25** in moderate yield and very high levels of diastereoselectivity. Presumably formation of the dianionic geminal bis(boronate), which would be required for the one-pot process, is disfavored due to steric hindrance. Attempts to homologate **15** with (+)-sparteine-ligated primary benzoates were unsuccessful, underscoring the sensitivity of primary–tertiary 1,2-bis(boronic esters) to steric hindrance. Pleasingly, both the selective homologation of the primary boronic ester moiety of **15** (to give secondary–tertiary 1,3-bis(boronic ester) **26**) and the double homologation of **15** (to give secondary–secondary 1,4-bis(boronic ester) **27**) could be carried out by using 0.9 and 2.1 equivalents, respectively, of diamine-free lithiated benzoate.

The regioselective single homologation of diborylmethane **1** with sparteine-ligated carbenoids, and the relative insensitivity of the diamine-free carbenoids to steric hindrance in their reactions with boronic esters, suggested that these types of carbenoids could be coupled in a one-pot three-component coupling reaction with diborylmethane **1** acting as a linchpin. Specifically, the generation of a (+)-sparteine-ligated carbenoid at –78 °C and the addition of diborylmethane **1** would give the mixed-valent diboryl species **29**. Subsequent addition of an enantiomerically pure α-stannyl benzoate followed by an equivalent portion of *n*-BuLi would lead to rapid and selective tin–lithium exchange followed by the reaction of the resulting diamine-free carbenoid with the remaining boronic ester moiety to give the heteroleptic geminal 1,1-bis(boronate) **30**. Finally, upon warming to room temperature 1,1-bis(boronate) **30** would undergo 1,2-metallate rearrangement at both boron centers to give the corresponding non-symmetrical secondary–secondary 1,3-bis(boronic esters) (Fig. 4C). This one-pot process was carried out for sparteine-ligated lithiated carbamate **28** ^24^ with a range of α-stannyl benzoates to give 1,3-*anti*-bis(boronic esters) **31–34** in moderate yield and with very high levels of diastereoselectivity. As is the nature of the transformation, 1,3-*syn*-bis(boronic esters), such as **35**, can be formed with equal ease and selectivity simply by using the enantiomeric α-stannyl benzoate. This transformation is a rare example of a fragment coupling reaction in which 1,3-related stereo centers are formed directly with complete control of the relative and absolute configuration, suggesting that it could be used as a strategy for bringing together advanced fragments for the preparation of complex molecules.

**Fig. 5:**
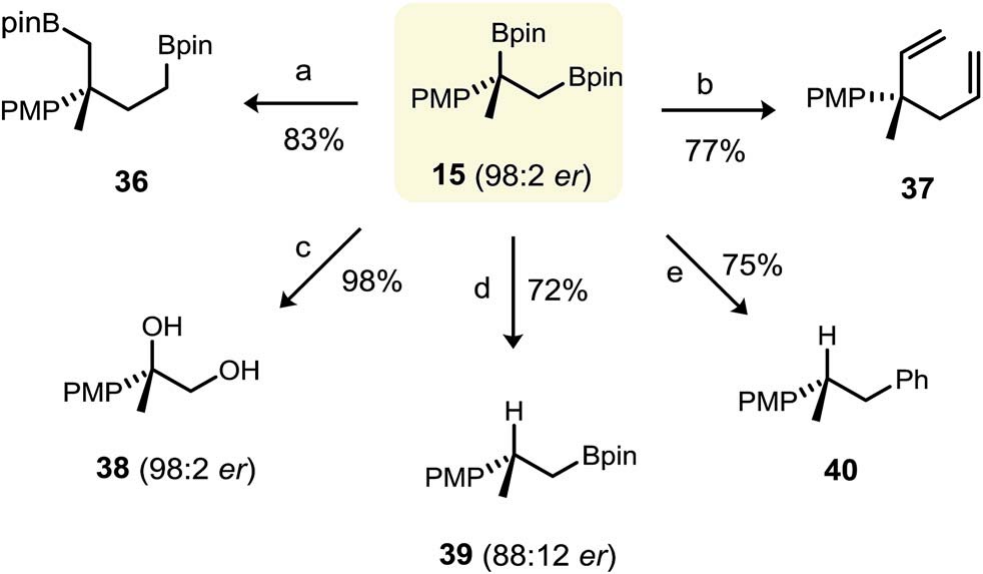
C–B functionalisation of primary–tertiary 1,2-bis(boronic ester) **15**. Reaction conditions: (a) BrCH_2_Cl (6 eq.), *n*-BuLi (5 eq.), Et_2_O, –78 °C (20 min); then RT (1 h); (b) vinyllithium (4.0 eq.), Et_2_O, –78 °C, 45 min; then I_2_/THF (4.0 eq.), –78 °C, 30 min; then NaOMe/MeOH (8.0 eq.), –78 °C to RT. (c) aq. 2 M NaOH/aq. 30% H_2_O_2_ (2 : 1), THF, 0 °C to RT, 4 h. (d) TBAF·3H_2_O (3.0 eq.), toluene, 90 °C, 2 h. (e) Pd(OAc)_2_/RuPhos (1 mol%), bromobenzene (1.5 eq.), KOH (3 eq.), THF/H_2_O, 70 °C, 12 h.

We briefly explored a number C–B functionalisation reactions of primary–tertiary 1,2-bis(boronic ester) **15** (Fig. 5). Its subjection to standard Matteson homologation,^25^ Zweifel olefination^26^ and standard oxidation conditions gave the corresponding difunctionalisation products **36–38**, respectively, in good yield. We were also particularly interested in whether selective functionalisation of either the primary or the tertiary boronic ester could be achieved. As expected, selective protodeboronation of the tertiary benzylic boronic ester of **15** (98 : 2 e.r.) was possible, but with only moderate enantiospecificity (primary boronic ester **39** was obtained in 88 : 12 e.r.).^27^ The group of Morken has shown that primary boronic esters can be transformed selectively in the presence of a vicinally-positioned secondary boronic ester under Suzuki cross-coupling conditions.^3*b*^ However, although facile cross coupling of the primary boronic ester in **15** was evident, the conditions were also favourable for protodeboronation of the tertiary boronic ester, giving 1,2-diarylpropane **40** in 75% yield. Clearly, further exploration and optimisation of the conditions for the group-selective functionalisation of primary–tertiary bis(boronic esters) is warranted.

## Conclusions

In summary, we have developed a regiodivergent strategy for the stereoselective synthesis of 1,2- and 1,3-bis(boronic esters) from commercially available diborylmethane **1**. Using hindered chiral carbenoids we have been able to effect the selective single homologation of diborylmethane **1** to prepare a wide range of 1,2-bis(boronic esters). Generating primary chiral carbenoids in the absence of diamines enabled the exclusive double homologation of diborylmethane **1** to afford *C*_2_-symmetric 1,3-bis(boronic esters). The strategic combination of these carbenoids (sparteine-ligated and diamine-free) with diborylmethane **1**, representing a formal carbenoid–carbenoid coupling reaction, allowed non-symmetrical 1,3-bis(boronic esters) to be prepared directly.
