# Supplementary material for: Selective uni- and bidirectional homologation of diborylmethane
Source: Chem Sci. 2017 Feb 9;8(4):2898–903. doi: 10.1039/c6sc05338f (PMC5376717; doi:10.1039/c6sc05338f)

Electronic Supplementary Material (ESI) for Chemical Science.
This journal is © The Royal Society of Chemistry 2017

Supporting Information

For

Selective Uni- and Bidirectional Homologation of Diborylmethane

Daniel J. Blair, Damiano Tanini, Joseph M. Bateman, Helen K. Scott, Eddie L. Myers, Varinder K. Aggarwal

Table of Contents

General Experimental Details S3

Synthesis of Starting Materials S4

Synthesis of Primary Benzoates S4
Synthesis of α-Stannyl Benzoates S7
Synthesis of Secondary Benzylic Carbamates S13

Synthesis of 1,2-bis(boronic esters) S14

Using Primary Benzoates S14
Using Secondary Benzylic Carbamates S19
Using Secondary Dialkyl Carbenoids S26

Synthesis of 1,3-bis(boronic esters) S27

C_2-Symmetric 1,3-bis(boronic esters) S27
Sequential Homologation using Secondary Carbamates S29
Non-symmetric 1,3-bis(boronic esters) through One-pot Double Homologation S32

C-B Functionalisation Reactions S35

References S39

^{1}H and ^{13}C NMR Spectra S41

General Experimental Details

Reaction mixtures were stirred magnetically. Air- and moisture-sensitive reactions were carried out in flame-dried glassware under argon atmosphere by using standard Schlenk manifold techniques. Fine chemicals were purchased from Acros Organics, Alfa Aesar, Inochem-Frontier Scientific or Sigma-Aldrich and used as received unless otherwise mentioned. *n*-Butyllithium (*n*BuLi) was received from Acros Organics as a 1.3 M solution in cyclohexane/hexane 92:8 and the molarity was verified by titration with *N*-benzylbenzamide.[1] TMEDA was distilled over CaH_2 before use; (–)-sparteine and (+)-sparteine were isolated from the commercially available sulfate salt following a procedure by Beak.[2] Petrol refers to the fraction of petroleum ether boiling at 40–60 °C. Anhydrous THF, CH_2Cl_2, toluene, hexane, acetonitrile and Et_2O were dried by passing through a modified Grubbs system[3] of alumina columns, manufactured by Anhydrous Engineering and were transferred under argon *via* syringe. Microwave reactions were carried out in a Biotage Initiator EXP EU microwave synthesiser. ^1H Nuclear Magnetic Resonance (NMR) spectra were recorded in $CDCl_3$ at 300, 400 or 500 MHz on a Joel Lambda 300, Joel ECP 400, a Varian 400-MR or a VNMRS500a Fourier transform spectrometer. Chemical shifts (δ_H) are quoted in parts per million (ppm) and referred to the residual protio solvent signals of $CHCl_3$ (7.27 ppm). ^1H NMR coupling constants are reported in hertz and refer to apparent multiplicities. Data are reported as follows: chemical shift, multiplicity (s = singlet, br. s = broad singlet, d = doublet, t = triplet, q = quartet, quin = quintet, sext = sextet, sept = septet, m = multiplet, dd = doublet of doublet, etc.), coupling constant, integration, and assignment. ^{13}C NMR spectra were recorded at 101 or 126 MHz. Chemical shifts (δ_C) are quoted in ppm and referenced to $CHCl_3$ (77.0 ppm) or acetone (29.92 ppm). ^{11}B NMR spectra were measured using Norell S-200-QTZ quartz NMR tubes at 96 or 128 MHz with complete proton decoupling. ^{19}F NMR spectra were recorded at 283, 376 or 470 MHz. Mass spectra were recorded by the University of Bristol, School of Chemistry departmental mass spectrometry service using electron impact ionisation (EI), chemical ionisation (CI) or electrospray ionisation (ESI) techniques for low- and high-resolution mass spectra. HRMS EI and CI were performed on a VG Analytical Autospec mass spectrometer at 70 eV. HRMS ESI was performed on either a Bruker Daltonics Apex IV, 7-Tesla FT-ICR or microTOF II. Samples were submitted in EtOAc. For low-resolution mass spectra (*m*/*z*) only molecular ions (M^+ or M+H^+) and major peaks are reported with intensities quoted as percentage of the base peak. All infrared spectra were recorded on the neat compounds using a PerkinElmer Spectrum One FT-IR spectrometer, irradiating between 4000 cm^{-1} and 600 cm^{-1}. Only strong and selected absorbance values (ν_{max}) are reported. Analytical TLC was performed on aluminium-backed silica plates (Merck, Silica Gel 60 F_{254}, 0.25 mm). Compounds were visualised by fluorescence quenching or by staining the plates with 5% solution of phosphomolybdic acid ($H_3PMo_{12}O_{40}$) in EtOH followed by heating. Flash column chromatography was performed on silica gel (Aldrich, Silica Gel 60, 40–63 μm). All mixed solvent eluents are reported as *v*/*v* solutions. Optical rotations were obtained using a Bellingham + Stanley Ltd. ADP220 polarimeter at 589 nm (Na D-line) in a cell with a path length of 1 dm. Specific rotation values are given in (deg mL)/(g dm). Melting points were measured with a Reichert hot stage apparatus and are uncorrected. The *ee* values of boronic ester products were determined through standard oxidation to the corresponding alcohol (see page S38) followed by either chiral HPLC or SFC analysis. Chiral high performance liquid chromatography (HPLC) separations were performed on an Agilent 1100 Series HPLC unit equipped with UV-vis diode-array detector monitored at 210.8 nm, using Daicel Chiralpak ADH, IA, IB or IC columns (4.6 × 250 mm^2, 5 μm) fitted with respective guards (4 × 10 mm^2). Chiral supercritical fluid chromatography (SFC) was performed using Diacel Chiralpak IA, IB and IC columns (4.6 × 250 mm × 5 μm) or a Whelk O-1 column (4.6 × 250 mm × 5 μm) on a Waters TharSFC system and monitored by DAD (diode array detector).

Synthesis of Primary Benzoates

General Procedure 1 (GP1): Benzoate synthesis from a primary alkyl bromide

Following Beak's procedure,[4] a biphasic mixture of 2,4,6-triisopropylbenzoic acid (20.2 g, 81.3 mmol, 1.0 eq.), $NBu_4(HSO_4)$ (2.21 g, 6.5 mmol, 0.08 eq.), NaOH (10.1 g, 252.0 mmol, 3.1 eq.) and primary alkyl halide (81.3 mmol, 1 eq.) in $CHCl_3$ (400 mL) and H_2O (320 mL) was stirred overnight at room temperature. The phases were separated and the aqueous phase extracted with CH_2Cl_2 (3 × 100 mL). The combined organic phases were washed with brine (300 mL), dried ($MgSO_4$) and concentrated under reduced pressure. The crude product was dissolved in pentane (60 mL) and the insoluble salts filtered off. The solvent was removed from the filtrate under reduced pressure to give benzoate ester which was used without further purification.

General procedure 2 (GP2): Benzoate synthesis from a primary alcohol

To a stirred solution of PPh_3 (11 mmol), secondary alcohol (10 mmol) and 2,4,6-triisopropylbenzoic acid (11.5 mmol) in THF (15 ml) at 0 °C (ice bath), was added DIAD (11 mmol) dropwise over 10 min. After stirring the mixture for 4 h at 0 °C (ice bath), the volatiles were removed in vacuo. The residue was dissolved in pentane (15 ml) and the resulting solution stirred in for 5 min. The white suspension was filtered and the filter cake washed with pentane (100 ml). The solvent was removed in vacuo and the residue purified by flash column chromatography to give pure benzoate.

3-(2,5-dimethyl-1*H*-pyrrol-1-yl)propyl 2,4,6-triisopropylbenzoate (41)

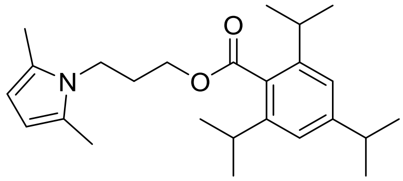

Using **GP2** and 3-(2,5-dimethyl-1*H*-pyrrol-1-yl)propan-1-ol[5] (1.5 g, 9.8 mmol) gave benzoate **41** (3.325 g, 89 %) after purification by column chromatography (10% Et_2O/pentane) as a colourless oil.

^{1}H NMR (400 MHz, $CDCl_3$): 7.02 (s, 2H), 5.78 (s, 2H), 4.35 (t, *J* 6.2, 2H), 3.88 (m, 2H), 2.90 (hept, *J* 6.9, 1H), 2.84 (hept, *J* 6.9, 2H), 2.22 (s, 6H), 2.05 (m, 2H), 1.26 (app. d, *J* 6.9, 18H).

^{13}C NMR (101 MHz, $CDCl_3$): 170.9, 150.4, 144.9, 130.3, 127.4, 120.9, 105.5, 62.3, 40.7, 34.4, 31.6, 30.2, 24.2, 23.9, 12.5.

IR (film): υ (cm^{-1}). 2958, 1720, 1251.

HRMS (ESI) calc. $C_{25}H_{38}NO_2$ $[M+H]^+$ 384.2897, found 384.2912.

pent-4-en-1-yl 2,4,6-triisopropylbenzoate (42)

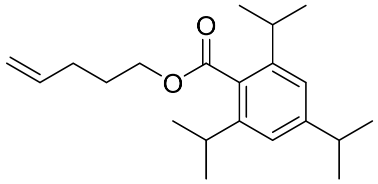

Using **GP1** and 5-bromopent-1-ene (5 mL, 40 mmol) gave benzoate **42** (8.12 g, 64%).

^{1}H NMR (400 MHz, $CDCl_3$): 7.01 (s, 2H), 5.82 (ddt, *J* 16.9, 10.2, 6.7, 1H), 5.05 (dq, *J* 16.9, 1.4, 1H), 5.01 (dq, *J* 10.2, 1.6, 1H), 4.31 (t, *J* 6.6, 2H), 2.95 – 2.76 (m, 3H), 2.18 (q, *J* 6.8, 2H), 1.83 (p, *J* 6.8, 2H), 1.25 (d, *J* 6.9, 12H), 1.24 (d, *J* 7.0, 6H).

^{13}C NMR (101 MHz, $CDCl_3$): 170.9, 150.1, 144.7, 137.3, 130.6, 120.8, 115.4, 64.3, 34.4, 31.5, 30.1, 27.8, 24.1, 23.9.

IR (film): υ (cm^{-1}) 2960, 2930, 2870, 1724, 1606, 1462, 1250, 1137, 1074, 912, 876.

HRMS (ESI) calc. $C_{21}H_{32}O_2Na$ $[M+Na]^+$ 339.2295, found 339.2295

(4*R*)-4-((5*R*,8*R*,9*S*,10*S*,13*R*,14*S*,17*R*)-3-((tert-butyldimethylsilyl)oxy)-10,13-dimethylhexadecahydro-1H-cyclopenta[a]phenanthren-17-yl)pentyl 2,4,6-tritriisopropylbenzoate (43)

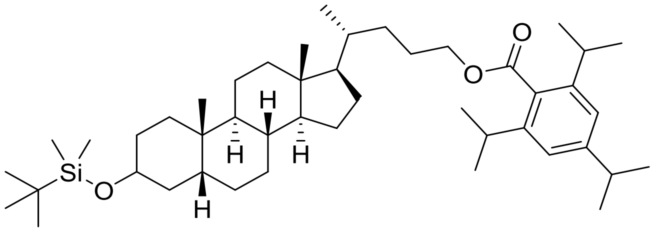

Using **GP2** and (4*R*)-4-((5*R*,8*R*,9*S*,10*S*,13*R*,14*S*,17*R*)-3-((*tert*-butyldimethylsilyl)oxy)-10,13-dimethylhexadecahydro-1H-cyclopenta[a]phenanthren-17-yl)pentan-1-ol[6] (3.01 g, 6.32 mmol) gave benzoate **43** (2.60 g, 58%) after column chromatography (20 % DCM/pentane).

^{1}H NMR (400 MHz, $CDCl_3$): 7.00 (s, 2H), 4.33–4.21 (m, 2H), 3.58 (m, 1H), 1.95 (d, *J* 12.0, 1H), 1.87–1.71 (m, 5H), 1.64–1.29 (s, 13H), 1.28–1.00 (m, 27H), 0.92 (d, *J* 6.5, 3H), 0.89 (s, 3H), 0.89 (s, 9H), 0.63 (s, 3H), 0.06 (s, 6H).

^{13}C NMR (101 MHz, $CDCl_3$): 171.03, 149.95, 144.68, 130.73, 120.78, 72.82, 65.62, 56.39, 56.27, 42.69, 42.29, 40.20, 40.16, 36.91, 35.85, 35.57, 35.54, 34.58, 34.40, 32.29, 31.46, 31.01, 28.32, 27.29, 26.39, 25.97, 25.45, 24.19, 24.17, 24.14, 23.94, 23.38, 20.80, 18.48, 18.33, 12.01, −4.61.

IR (film): υ (cm^{-1}) 2927, 2862, 1725, 1606, 1461, 1381, 1249, 1136, 871, 834, 772, 667.

HRMS (ESI) calc. $C_{46}H_{78}O_3SiNa$ [*M*+Na]$^+$ 729.5612, found 729.5607.

$[\alpha]_D^{23}$ +24 (*c* 1, $CHCl_3$)

pent-4-yn-1-yl 2,4,6-triisopropylbenzoate (44)

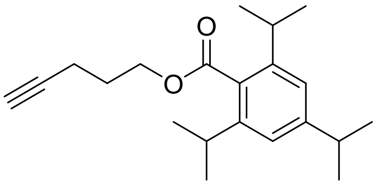

Using **GP2** and pent-4-yn-1-ol (3 mL, 32 mmol) gave benzoate **44** (7.36 g, 74%) after purification by column chromatography (1% Et_2O/pentane).

^{1}H NMR (400 MHz, $CDCl_3$): 7.01 (s, 2H), 4.41 (t, *J* 6.3, 2H), 2.89 (hept, *J* 6.9, 1H), 2.85 (hept, *J* 6.8, 2H), 2.34 (td, *J* 7.0, 2.6, 2H), 1.99 (t, *J* 2.6, 1H), 1.95 (p, *J* 6.9, 2H), 1.25 (d, *J* 6.9, 18H).

^{13}C NMR (101 MHz, $CDCl_3$): 170.8, 150.1, 144.7, 130.4, 120.8, 82.7, 69.2, 63.3, 34.4, 31.5, 27.4, 24.1, 23.9, 15.2.

IR (film): υ (cm^{-1}) 3290, 2961, 2930, 2871, 1725, 1606, 1249, 1073, 876, 631.

HRMS (ESI) calc. $C_{21}H_{30}O_2Na$ [*M*+Na]$^+$ 337.2138, found 337.2141.

3-((*tert*-butyldimethylsilyl)oxy)propyl 2,4,6 triisopropylbenzoate (45)

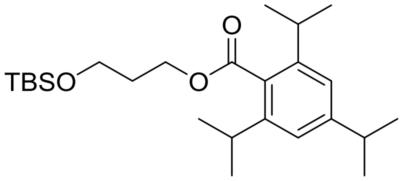

Using **GP2** and 3-((*tert*-butyldimethylsilyl)oxy)propan-1-ol[7] (0.089 g, 0.47 mmol) gave benzoate **45** (0.135 g, 68 %) after purification by column chromatography (1% Et_2O/pentane) as a colourless oil.

^{1}H NMR (400 MHz, $CDCl_3$): 7.00 (s, 2H), 4.40 (t, *J* 6.2, 2H), 3.72 (t, *J* 6.2, 2H), 2.88 (sept, *J* 6.9, 1H) 2.84 (sept, *J* 6.9, 2H), 1.93 (p, *J* 6.2, 2H), 1.24 (d, *J* 6.7, 18H), 0.89 (s, 9H), 0.05 (s, 6H).

^{13}C NMR (101 MHz, $CDCl_3$): 171.0, 150.1, 144.8, 130.7, 120.9, 62.1, 59.6, 34.5, 32.0, 31.6, 26.0, 24.2, 24.0, 18.3, −5.3.

IR (film): *υ* (cm^{-1}). 2958.3, 2869.2, 1726.0, 1462.1, 1250.4, 1073.4 and 835.7.

HRMS (ESI) calc. $C_{25}H_{44}NaO_3Si$ $[M+Na]^+$ 443.2952, found 443.2940.

Synthesis of Stannyl Benzoates

General Procedure 3 (GP3)

The requisite alkyl 2,4,6-triisopropylbenzoate (1.0 eq.) and (+)-sparteine (1.3 eq.) were dissolved in anhydrous diethyl ether (0.33 M) and cooled to –78 °C. *s*BuLi (1.3 M in hexanes, 1.3 eq.) was added dropwise and the reaction mixture was stirred at this temperature for 3–5 h. A trialkyltin chloride (1.5 eq) was added at −78 °C, and after 5 minutes the reaction mixture was warmed to room temperature. The reaction mixture was diluted with Et_2O (10 mL/mmol) and washed with equal volume of 1 M aq. HCl. The organic layer was separated, dried over $MgSO_4$, filtered, concentrated *in vacuo* and the residue purified by column chromatography. *Racemic samples were prepared using TMEDA in place of (+)-sparteine.*

(*R*)-3-(4-methoxyphenyl)-1-(tributylstannyl)propyl 2,4,6-triisopropylbenzoate (46)

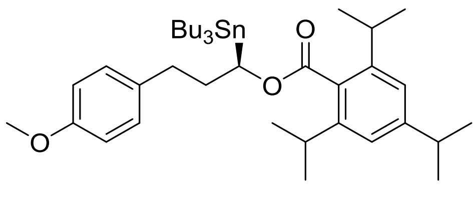

Using **GP3**, 3-(4-methoxyphenyl)propyl-2,4,6-triisopropylbenzoate[8] (3.00 g, 7.5 mmol) and tributyltin chloride (2.7 mL, 9.75 mmol) gave **46** (3.78 g, 73%) after column chromatography (5% Et_2O/pentane).

^{1}H NMR (400 MHz, $CDCl_3$): 7.09 (d, *J* 8.6, 2H), 7.01 (s, 2H), 6.84 (d, *J* 8.6, 2H), 5.17 (dd, *J* 9.0, 4.0, 1H), 3.79 (s, 3H), 2.97–2.80 (m, *J* 6.8, 3H), 2.73 (ddd, *J* 15.4, 10.7, 4.9, 1H), 2.61 (ddd, *J* 13.6, 10.5, 6.0, 1H), 2.23 (m, 1H), 2.10 (m, 1H), 1.62 – 1.40 (m, 6H), 1.31 (p, *J* 7.3, 6H), 1.26 (d, *J* 6.7, 12H), 1.25 (d, *J* 6.8, 6H), 1.01–0.93 (m, 6H), 0.89 (t, *J* 7.3, 9H).

^{13}C NMR (100 MHz, $CDCl_3$,): 171.1, 157.8, 149.8, 144.8, 133.8, 130.9, 129.2, 120.8, 113.8, 71.5, 55.2, 36.8, 34.4, 33.8, 31.5, 29.1, 27.5, 24.6, 24.2, 23.9, 13.7, 9.8.

IR (film): υ (cm^{-1}) 2957, 2925, 2870, 2854, 1706, 1608, 1512, 1245, 1068, 876, 819.

HRMS (ESI) calc. $C_{38}H_{62}NaO_3Sn$ $[M+Na]^+$ 709.3621, found 709.3604.

$[\alpha]_D^{22} = -27$ (*c* 1, $CHCl_3$).

Despite much effort, an *ee* value could not be obtained for this compound owing to poor separation of the racemate into enantiomers by using chiral HPLC. However, the high d.r value obtained in its use for the synthesis of C_2-symmetric 1,3-bis(boronic ester) **4**, suggests that the *ee* value is >95:5; a value of 96:4 is typical for the asymmetric deprotonation of similar benzoate esters.

(*S*)-1-(Trimethylstannyl)ethyl 2,4,6-triisopropylbenzoate (47)

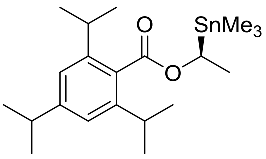

According to **GP3** ethyl 2,4,6-triisopropyl benzoate[9] (14 g, 60 mmol, 1 eq.), (−)-sparteine (17 mL, 74 mmol, 1.2 eq.) and trimethyltin chloride (1 M in hexanes, 80 mL, 80 mmol, 1.3 eq.) gave a yellow solid (~95:5 *er*) after work up, which was purified and enriched by recrystallization (MeOH 4mL/g) giving **47** as long cubic prisms (61%, 16.2 g, >99:1 *er*) and a second crop of crystals from the mother liquor (25%, 6.6 g, 92:8 *er*).

M.p. 64–65 °C (MeOH), Lit.[9] 65–66 °C (MeOH)

^{1}H NMR ($CDCl_3$, 400 MHz): 6.99 (s, 2H), 5.04 (q, *J* 6.9, 1H), 2.80–2.95 (m, 3H), 1.58 (m, 3H), 1.24 (d, *J* 6.9, 18H), 0.18 (s, d, *J* 54.1, and d, *J* 51.7, 9H).

^{13}C NMR ($CDCl_3$, 100 MHz): 173.3, 149.9, 144.8, 120.8, 67.0, 34.4, 31.3, 24.3, 24.1, 24.1, 23.9, −9.9.

$[\alpha]_D^{22} = +40$ (*c*. 1, $CHCl_3$), Lit.[9] $[\alpha]_D^{20} = +38.3$ (*c*. 1.1, $CHCl_3$)

Chiral HPLC (IB , hexane, 0.7 mL/min, RT) t_R = 7.5 (major) and 10.0 (minor).

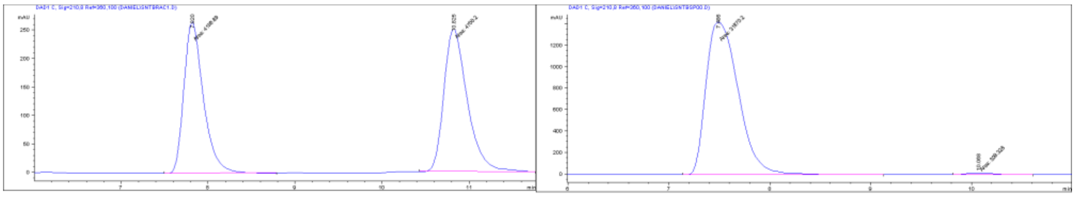

(*R*)-1-(Trimethylstannyl)ethyl 2,4,6-triisopropylbenzoate (*ent*-47)

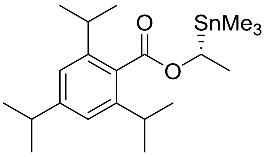

According to **GP3** using ethyl 2,4,6-triisopropylbenzoate (5.5 g, 30 mmol, 1 eq.),[9] (+)-sparteine surrogate (6.7 g, 37 mmol, 1.2 eq.) or (+)-sparteine (9.1 g, 37 mmol, 1.2 eq.), and trimethyltin chloride (1 M in hexanes, 40 mL, 40 mmol, 1.3 eq.) gave a yellow solid, which was purified and enriched by recrystallization (MeOH 4mL/g) giving *ent*-**47** as long cubic prisms (62 %, 5.3 g, >99:1 *er*) and a second crop of crystals from the mother liquor (20%, 1.7 g, 87:13 *er*).

M.p. 64–65 °C (MeOH)

$[\alpha]_D^{22} = -40$ (*c*. 1, $CHCl_3$)

Chiral HPLC (IB, hexane, 0.7 mL/min, RT), t_R = 7.7 (minor) and 10.4 (minor).

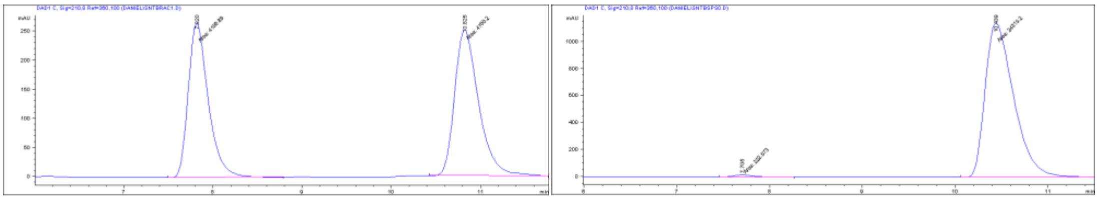

(*R*)-3-phenyl-1-(trimethylstannyl)propyl 2,4,6-triisopropylbenzoate (48)

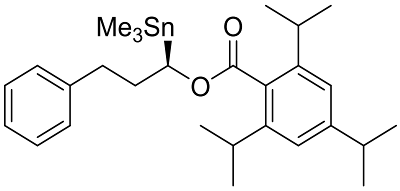

Using **GP3**, 3-phenylpropyl-2,4,6-triisopropylbenzoate[10] (2.31 g, 6.3 mmol) and trimethyltin chloride (6.0 mL, 9.5 mmol, 1.57 M in hexane) gave **48** (1.66 g, 50%, 95:5 *er*) after column chromatography (3% Et_2O/pentane).

^{1}H NMR (400 MHz, $CDCl_3$): 7.30 (m, 2H), 7.24–7.14 (m, 3H), 7.02 (s, 2H), 5.01 (m, 1H), 2.97–2.84 (m, 3H), 2.81 (ddd, *J* 13.4, 10.7, 5.9, 2H), 2.68 (ddd, *J* 13.4, 10.7, 5.9, 2H), 2.32–2.08 (m, 2H), 1.27 (12H, d, *J* 6.8), 1.26 (6H, d, *J* 6.9), 0.22 (s, d, *J* 53.9 and d, *J* 51.6, 9H).

^{13}C NMR (101 MHz, $CDCl_3$): 171.4, 150.0, 144.9 , 141.6, 130.6, 128.4, 128.3, 125.9, 120.9, 71.8, 36.0, 34.4, 34.4, 31.5, 24.4, 24.3, 23.9, −9.0.

$[\alpha]_D^{22} = -36$ (*c* 1, $CHCl_3$).

IR (film): υ (cm^{-1}) 2961, 2931, 2868, 1704, 1606, 1573, 1248, 1068, 766, 698.

HRMS (ESI) calc. $C_{28}H_{42}NaO_2Sn$ [M+Na]$^+$ 553.2104, found 553.2091.

Chiral HPLC (IA, 97.5:2.5 hexane:IPA, 1 mL/min, RT)

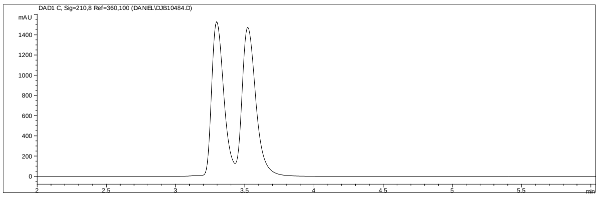

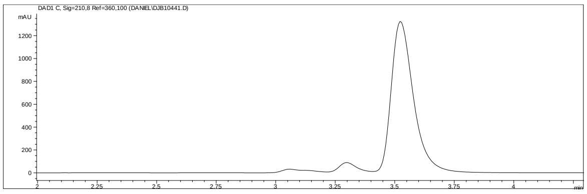

3-((*tert*-butyldimethylsilyl)oxy)-1-(trimethylstannyl)propyl 2,4,6 triisopropylbenzoate (49)

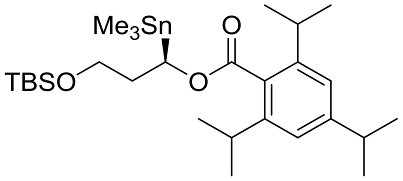

Using **GP3** and benzoate **45** (2.00 g, 4.75 mmol) gave stannyl benzoate **49** (2.40 g, 87%, 95:5 *er*) after purification by column chromatography (4% Et_2O/pentane) as a colourless oil.

^{1}H NMR (400 MHz, $CDCl_3$): 6.99 (s, 2H), 5.07 (dd, *J* 9.4, 4.2, 1H), 3.64-3.75 (m, 2H), 2.88 (hept, *J* 6.8, 1H), 2.81 (hept, *J* 6.8, 2H), 2.15 (m, 1H), 2.03 (dtd, *J* 14.3, 7.10, 7.10, 4.3, 1H), 1.24 (d, *J* 7.00, 6H), 1.23 (d *J* 7.00, 12H), 0.89 (s, 9H), 0.20 (s and d, *J* 54 and d, *J* 51, 9H), 0.04 (s, 6H).

^{13}C NMR (101 MHz, $CDCl_3$): 171.4, 150.0, 145.0, 130.8, 121.0, 68.7, 61.1, 37.0, 34.5, 31.6, 26.1, 24.5, 24.1, 18.5, −5.2, −8.8.

$[\alpha]_D^{22}$ = – 34 (c = 1.0, $CHCl_3$)

IR (film): υ (cm^{-1}) 2959, 2928, 2859, 1705, 1251, 1099, 835.

HRMS (ESI) calc. $C_{28}H_{52}NaO_3SiSn$ [M+Na]$^+$ 607.2605, found 607.2603.

CHIRAL HPLC (Chiralpak IB, hexane, 1 ml/min, RT) t_R: 5.63 min (minor), 6.54 min (major), *er* 95:5.

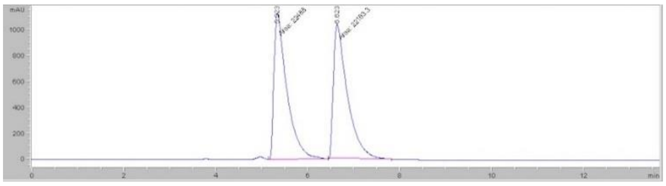

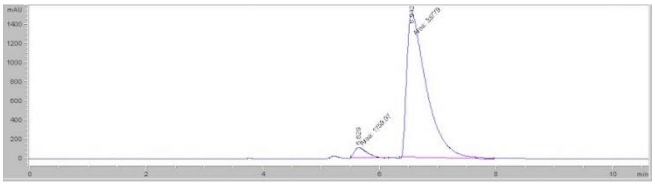

(*R*)-1-(trimethylstannyl)pent-4-en-1-yl 2,4,6-triisopropylbenzoate (50)

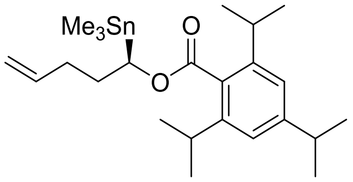

Using **GP3**, benzoate **42** (2.31 g, 6.3 mmol) and trimethyltin chloride (6.0 mL, 9.5 mmol, 1.57 M in hexane) gave **50** (1.56 g, 51%, 95:5 *er*) after column chromatography (1% Et_2O/pentane).

^{1}H NMR (400 MHz, $CDCl_3$): 7.00 (s, 2H), 5.81 (ddt, *J* 16.9, 10.2, 6.5, 1H), 5.07–4.93 (m, 2H), 2.95–2.76 (m, 3H), 2.28–2.09 (m, 2H), 2.04 (dtd, *J* 14.4, 8.8, 5.5, 1H), 1.93 (dddd, *J* 14.4, 9.9, 6.1, 4.9, 1H), 1.26–1.22 (d, *J* 6.9, 18H), 0.20 (s, d *J* 53.9 and d, *J* 51.6, 9H).

^{13}C NMR (101 MHz, $CDCl_3$): 171.3, 149.9, 144.8, 137.7, 130.7, 120.8, 115.1, 71.6, 34.4, 33.2, 31.9, 31.4, 24.4, 24.2, 23.9, –9.1.

IR (film): υ (cm^{-1}) 2962, 2928, 2872, 1705, 1462, 1249, 1075, 769

HRMS (ESI) calcd. for $C_{24}H_{40}NaO_2Sn$ $[M+Na]^+$ 503.1947, found 503.1927.

$[\alpha]_D^{22} = -41$ (*c* 1, $CHCl_3$).

The enantiomeric ratio of **50** was determined through tin–lithium exchange and reaction with 4,4,5,5-tetramethyl-2-phenethyl-1,3,2-dioxaborolane as outlined below. Subsequent oxidation and chiral HPLC separation as previously described[11] showed the *er* to be 95:5.

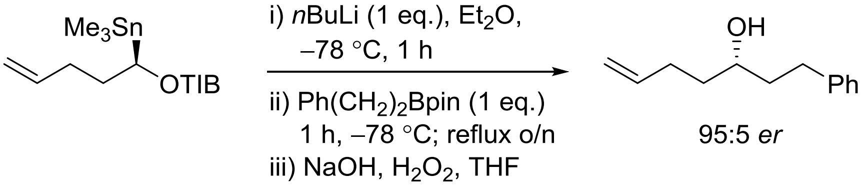

Chiral HPLC (Chiralpak IB, 95:5 hexane:IPA, 0.5 ml/min, RT) t_R: 15.09 min (minor), 20.14 min (major), *er* 95:5.

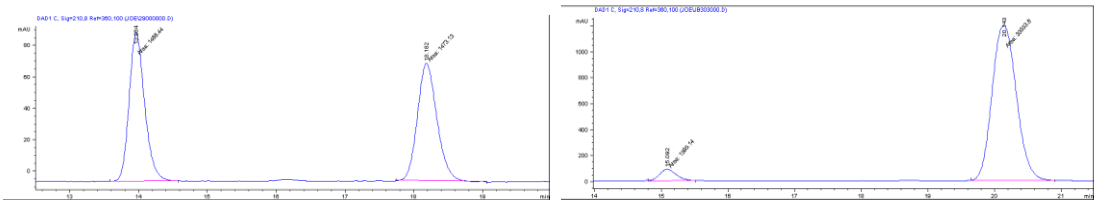

(*R*)-2-methyl-1-(trimethylstannyl)propyl 2,4,6-triisopropylbenzoate (51)

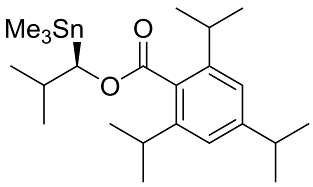

Using **GP3**, isobutyl 2,4,6-triisopropylbenzoate[10] (1.9 g, 6.3 mmol) and trimethyltin chloride (6.0 mL, 9.5 mmol, 1.57 M in hexanes) gave **51** (1.25 g, 42%, ≥96:4 *er*) after column chromatography (1% Et_2O/pentane).

^{1}H NMR (400MHz, $CDCl_3$): 7.00 (s, 2H), 4.97 (m, 1H), 2.93–2.79 (m, 3H), 2.25 (m, 1H), 1.25 (d, *J* 6.9, 6H), 1.24 (d, *J* 6.9, 6H), 1.23 (d, *J* 7.0, 6H), 1.04 (d, *J* 6.7, 3H), 1.00 (d, *J* 6.8, 3H), 0.21 (s, d, *J* 53.6 and d, *J* 51.4, 9H).

^{13}C NMR (101 MHz, $CDCl_3$): 171.4, 149.8, 144.9, 130.8, 120.8, 79.4, 34.3, 32.6, 31.4, 24.4, 24.2, 23.9, 21.3, 19.8, −8.0.

IR (film): υ (cm^{-1}) 2960, 2928, 2872, 1703, 1609, 1576, 1250, 1138, 1075, 1065, 876, 766.

HRMS (ESI) calc. $C_{23}H_{40}NaO_2Sn$ $[M+Na]^+$ 491.1947, found 491.1650.

$[\alpha]_D^{22}$ = −27 (*c* 1, $CHCl_3$).

Chiral HPLC (Chiralpak IB, hexane, 1 ml/min, RT) t_R: 4.24 min (minor), 5.38 min (major), *er* 96:4.

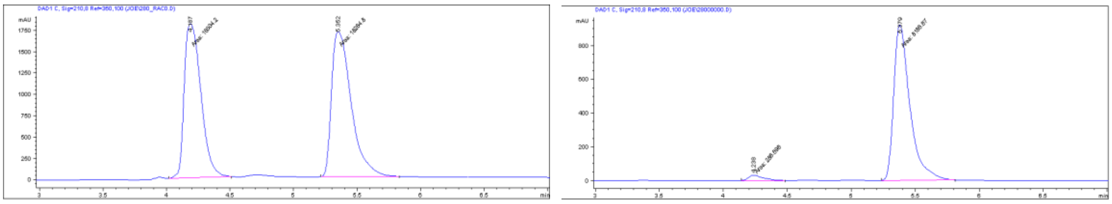

Synthesis of Secondary Benzylic Carbamates

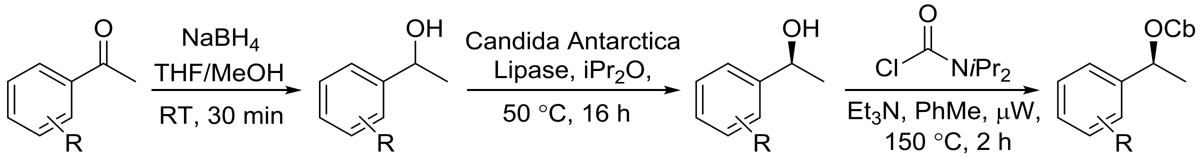

$NaBH_4$ (567 mg, 15.0 mmol, 1.5 eq.) was added portion-wise to a solution of ketone (10.0 mmol, 1 eq.) in MeOH (7 mL) and THF (10 mL) at 0 °C over 5 minutes with vigorous stirring. The reaction was warmed to RT and stirred for 30 min at which point TLC indicated complete consumption of starting material. The reaction was quenched by addition of saturated aq. NH_4Cl (5 mL) and diluted with H_2O (10 mL) and EtOAc (100 mL). The layers were separated and the organic layer was washed sequentially with H_2O (15 mL) and brine (2 × 15 mL). The organic layer was dried over $MgSO_4$, filtered, concentrated *in vacuo* and used without further purification. To a solution of racemic benzylic alcohol (9.8 mmol, 1 eq.) in diisopropyl ether (4 mL) was added acrylic-resin-bound lipase from *Candida Antarctica* (59 mg, 6 mg per mmol of alcohol) followed by vinyl acetate (4.3 mL, 49 mmol 5 eq.). The suspension was then heated to 50 °C, stirred for 16 h at which point ^{1}H NMR analysis of a filtered aliquot indicated ≥50% conversion. The reaction mixture was filtered through a plug of SiO_2, which was washed with EtOAc. The filtrate was concentred *in vacuo* and purified by flash column chromatography (20% EtOAc:petrol) to give the enantioenriched (*S*)-alcohol and (*R*)-acetate products. To a solution of the enantioenriched benzylic alcohol (3.47 mmol, 1.00 eq.) in PhMe (3.5 mL) in a sealable microwave vial under N_2 was added *N*,*N*-diisopropylcarbamoyl chloride (681 mg, 4.16 mmol, 1.20 eq.) followed by Et_3N (0.63 mL, 4.51 mmol, 1.30 eq.). The vial was then sealed and heated under microwave irradiation at 150 °C for 2 h.* The reaction mixture was then cooled to room temperature, filtered through a plug of SiO_2 with Et_2O, concentrated *in vacuo* and purified by column chromatography to afford secondary carbamates in >99:1 *er*. *** The reaction can be performed under standard reflux by replacing the PhMe with DCM (0.5 M) for 24–48h.** Analytical data for the carbamates below can be found in the supporting information of previous work.[12–15]

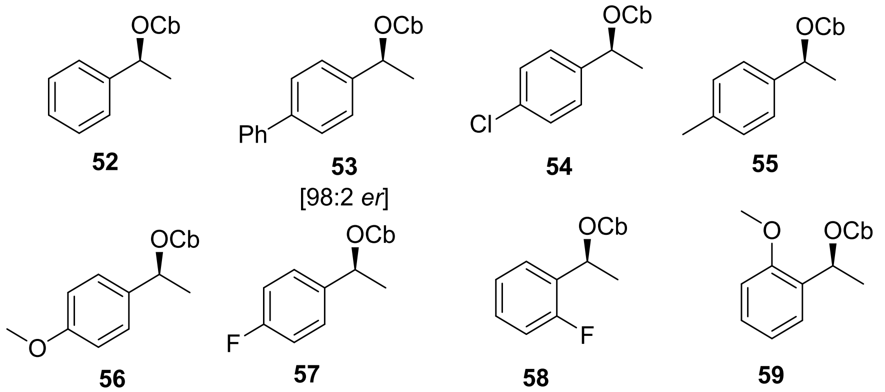

52 53 [98:2 *er*] 54 55

56 57 58 59

Synthesis of 1,2-bis(boronic esters)

General Procedures

General procedure for single homologation of diborylmethane 1 using primary benzoates (GP4)

A solution of alkyl 2,4,6-triisopropylbenzoate (1.0 eq.) and (+)-sparteine (1.2 eq.) in anhydrous diethyl ether (0.33 M) was cooled to −78 °C. *s*BuLi (1.3 M in hexanes,1.2 eq.) was added dropwise and the reaction mixture was stirred at this temperature for 3–5 h. A solution of bis(4,4,5,5-tetramethyl-1,3,2-dioxaborolan-2-yl)methane (1.5 eq.) in anhydrous diethyl ether (0.75 M) was added dropwise and the mixture was stirred for 1 h at −78 °C. Afterwards, the cooling bath was removed and the reaction mixture was heated at 40 °C overnight (16 h). The reaction mixture was cooled to room temperature, diluted with water and extracted with Et_2O. The combined organic phases were washed with 1 M HCl solution, dried over $MgSO_4$, filtered and concentrated *in vacuo*. The crude material purified by flash chromatography on silica gel to give the 1,2-bis(boronic ester). *Racemic samples were prepared using TMEDA or nBu-bispidine in place of (+)-sparteine.*

(*R*)-2,2'-(4-(4-methoxyphenyl)butane-1,2-diyl)bis(4,4,5,5-tetramethyl-1,3,2-dioxaborolane) (3)

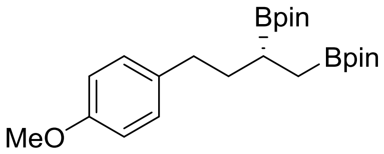

Following **GP4** with 3-(4-methoxyphenyl)propyl 2,4,6-triisopropylbenzoate[8] (0.63 mmol, 250 mg), gave **3** as a colourless oil (71%, 242 mg, 97:3 *er*).

^{1}H NMR (400 MHz, $CDCl_3$): 7.09 (app. d, *J* 8.8, 2H), 6.80 (app. d, *J* 8.8, 2H), 3.77 (s, 3H), 2.55 (t, *J* 8.2, 2H), 1.60 (m, 1H), 1.75 (m, 1H), 1.25 (s, 12H), 1.23 (s, 12H), 1.18 (m, 1H), 0.93 (dd, *J* 15.7, 9.4, 1H), 0.86 (dd, *J* 15.7, 5.9, 1H).

^{13}C NMR (100 MHz, $CDCl_3$): 157.5, 135.4, 129.2, 113.6, 82.8, 55.2, 36.2, 34.4, 24.9, 24.9, 24.8, 18.3, 12.4.

IR (film): υ (cm^{-1}) 2977, 2929, 1614, 1512, 1370, 1311, 1244, 1140, 967, 845, 824, 671.

HRMS (ESI) calcd. for $C_{23}H_{38}B_2NaO_5$ $[M+Na]^+$439.2806, found 439.2802.

$[\alpha]_D^{22} = -7$ (*c* 1, $CHCl_3$).

Chiral SFC: Chiralcel IB , 125 bar, 42°C, 4 mL/min, 10% co-solvent (50% IPA/Hexane); t_R: 13.57 min (minor), 14.17 min (major).

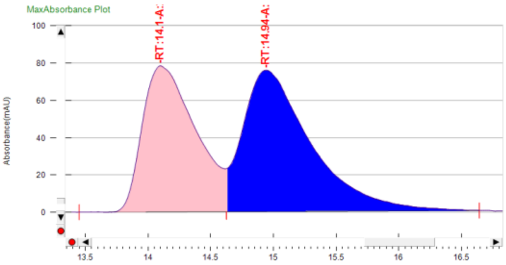

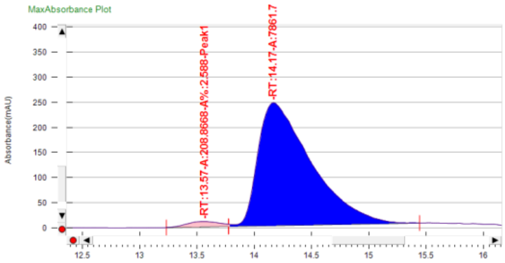

(*R*)-1-(3,4-bis(4,4,5,5-tetramethyl-1,3,2-dioxaborolan-2-yl)butyl)-2,5-dimethyl-1*H*-pyrrole (5)

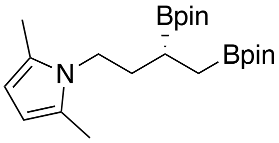

Using **GP4** and benzoate **41** (1.50 g, 3.91 mmol) gave 1,2-bis(boronic ester) **5** (0.987 g, 63 %, 97:3 *er*) after purification by column chromatography (10 % Et_2O/pentane) as a pale yellow oil.

^{1}H NMR (400 MHz, $CDCl_3$): 5.73 (s, 2H), 3.81 – 3.64 (ABddd, 2H), 2.22 (s, 6H), 1.78 – 1.68 (m, 1H), 1.68 – 1.59 (m, 1H), 1.24 (d, J = 6.3 Hz), 1.23 – 1.71 (m, 1H), 0.97 (dd, J = 15.9, 8.9 Hz, 1H), 0.86 (dd, J = 15.9, 6.1 Hz, 1H)

^{13}C NMR (101 MHz, $CDCl_3$): 127.4 (C), 104.8 (CH), 83.3 (C), 83.2 (C), 43.4 (CH_2), 34.8 (CH_2), 25.0 (CH_3), 24.9 (CH_3), 12.5 (CH_3) *carbons attached to boron not observed.*

$[\alpha]_D^{22}$ = +1 (c = 1.0, $CHCl_3$)

IR (film): υ (cm^{-1}) 2976, 2929, 1519, 1409, 1369, 1314, 1140.

HRMS (ESI) calc. $C_{22}H_{39}B_2NaNO_4$ $[M+Na]^+$ 426.2965, found 426.2966.

CHIRAL HPLC: (Chiralpak IB, 50:50 hexane:IPA, 1 ml/min, RT) t_R: 4.23 min (minor), 5.48 min (major), *er* 97:3.

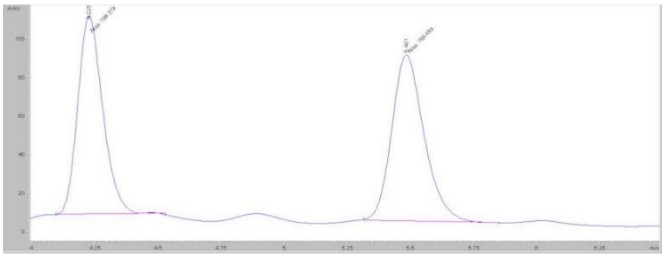

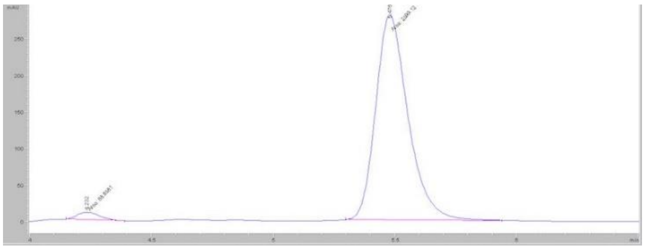

(*R*)-2,2'-(hex-5-ene-1,2-diyl)bis(4,4,5,5-tetramethyl-1,3,2-dioxaborolane) (6)

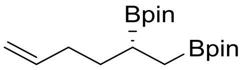

Following **GP4** with benzoate **42** (0.25 mmol, 79 mg), gave **6** as a colourless oil (74%, 62 mg, 97:3 *er*). The *ee* value was determined through oxidation and formation of the corresponding dimethyl acetal.

^{1}H NMR (400 MHz, $CDCl_3$): 5.81 (ddt, *J* 17.1, 10.2, 6.7, 1H), 4.98 (dq, *J* 17.1, 1.5, 1H), 4.89 (d, *J* 10.2, 1H), 2.06 (app. q, *J* 7.2, 2H), 1.56 (m, 1H), 1.40 (m, 1H), 1.23 (s, 12H), 1.22 (s, 12H), 1.14 (p, *J* 6.7, 1H), 0.85 (m, 2H).

^{13}C NMR (101 MHz, $CDCl_3$): 139.4, 114.0, 82.8, 82.8, 33.1, 33.1, 24.9, 24.8, 24.8, 24.7, *carbons attached to boron not observed.*

IR (film): υ (cm^{-1}) 3076, 2978, 2926, 1640, 1369, 1312, 1142, 968, 848, 671.

HRMS (ESI) calcd. for $C_{18}H_{34}B_2NaO_4$ $[M+Na]^+$ 359.2542, found 359.2545.

$[\alpha]_D^{22} = -1$ (*c* 1, $CHCl_3$).

Chiral GC: β-Dextrose column, 50 °C isocratic, 0.1 mL/min; t_R: 37.01 min (minor), 37.14 min (major), er 97:3.

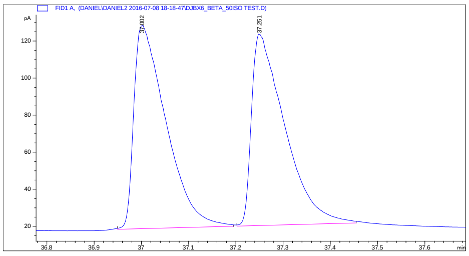

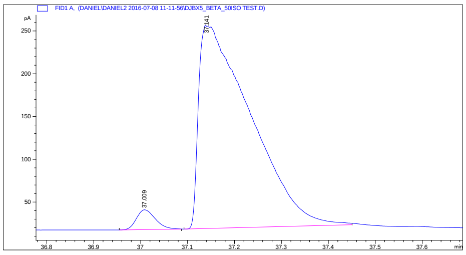

(((3*R*,5*R*,8*R*,9*S*,10*S*,13*R*,14*S*,17*R*)-17-((2*R*,5*S*)-5,6-bis(4,4,5,5-tetramethyl-1,3,2-dioxaborolan-2-yl)hexan-2-yl)-10,13-dimethylhexadecahydro-1H-cyclopenta[a]phenanthren-3-yl)oxy)(*tert*-butyl)dimethylsilane (7)

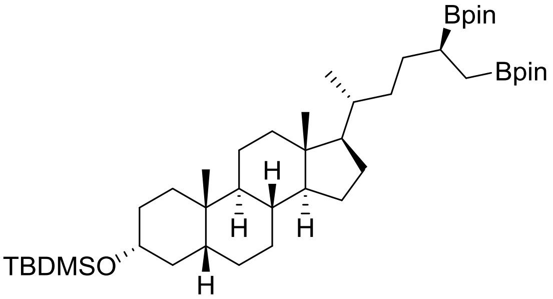

Following **GP4** with benzoate **43** (0.33 mmol, 233 mg) gave **7** as a colourless oil (73%, 228 mg, >20:1 dr).

^{1}H NMR (400 MHz, $CDCl_3$): 3.59 (m, 1H), 1.94 (app. d, *J* 12.0, 1H), 1.73–1.86 (m, 4H), 1.49–1.57 (m, 2H), 1.28–1.43 (m, 11H), 1.27–1.17 (m, 27H), 1.01–1.15 (m, 8H), 0.91–0.85 (m, 16H), 0.80 (dd, *J* 15.7, 6.1, 1H), 0.61 (s, 3H), 0.06 (s, 6H).

^{13}C NMR (100 MHz, $CDCl_3$): 82.7, 82.7, 72.8, 56.4, 56.1, 42.6, 42.3, 40.2, 40.1, 36.9, 36.0, 35.9, 35.6, 35.1, 34.6, 31.0, 30.2, 28.2, 27.3, 26.4, 26.0, 25.0, 24.9, 24.8, 24.7, 24.2, 23.4, 20.8, 18.8, 18.7, 18.3, 12.6, 12.0, −4.6.

IR (film): υ (cm^{-1}) 2975, 2927, 2863, 1463, 1449, 1370, 1311, 1250, 1142, 1094, 1079, 968, 870, 835, 774, 758, 668.

HRMS (ESI) calcd. for $C_{43}H_{84}B_2NO_5Si$ [M+NH_4]$^+$ 744.6314, found 744.6330.

$[\alpha]_D^{26} = -15$ (c 1, $CHCl_3$).

2,2'-((2*S*,3*R*,5*S*)-6-(methoxymethoxy)-3,5-dimethylhexane-1,2-diyl)bis(4,4,5,5-tetramethyl-1,3,2-dioxaborolane) (8)

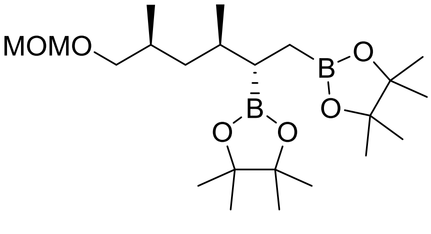

Following **GP4** with (2*R*,4*S*)-5-(methoxymethoxy)-2,4-dimethylpentyl diisopropylcarbamate[16] (0.33 mmol, 100 mg), (–)-sparteine (0.36 mmol, 85 mg), and bis(4,4,5,5-tetramethyl-1,3,2-dioxaborolan-2-yl)methane (0.43 mmol, 115 mg) gave 2,2'-((2*S*,3*R*,5*S*)-6-(methoxymethoxy)-3,5-dimethylhexane-1,2-diyl)bis(4,4,5,5-tetramethyl-1,3,2-dioxaborolane) as a colourless oil (61%, 111 mg, >20:1 dr).

^{1}**H NMR** (400 MHz, $CDCl_3$): 4.60 (s, 2H), 3.44 (dd, *J* 9.3, 4.7, 1H), 3.34 (s, 3H), 3.21 (dd, *J* 9.3, 7.4, 1H), 1.71–1.78 (m, 2H), 1.38 (m, 1H), 1.23 (s, 12H), 1.22 (s, 12H), 1.13 (dt, *J* 11.9, 4.4, 1H), 1.00–0.90 (m, 4H), 0.86 (d, *J* 6.8, 3H), 0.81 (m, 1H), 0.67 (dd, *J* 16.0, 4.6, 1H).

^{13}**C NMR** (100 MHz, $CDCl_3$): 96.5, 82.8, 82.7, 73.2, 55.0, 39.3, 32.5, 31.1, 25.0, 24.9, 24.7, 24.7, 18.6, 18.4, *carbons attached to boron not observed*.

IR (film): υ (cm^{-1}) 2975, 2901, 1406, 1379, 1310, 1142, 1066, 1050, 892.

HRMS (ESI) calcd. for $C_{22}H_{44}B_2NaO_6$ $[M+Na]^+$ 449.3224, found 449.3229.

$[\alpha]_D^{22}$ = +12 (*c* 1, $CHCl_3$).

2,2'-((2*R*,3*R*,5*S*)-6-(methoxymethoxy)-3,5-dimethylhexane-1,2-diyl)bis(4,4,5,5-tetramethyl-1,3,2-dioxaborolane) (9)

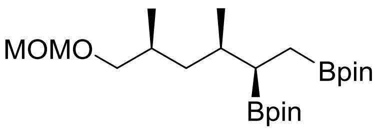

Following **GP4** with (2*R*,4*S*)-5-(methoxymethoxy)-2,4-dimethylpentyl diisopropylcarbamate[16] (0.33 mmol, 100 mg), gave **9** as a colourless oil (69%, 126 mg, >20:1 dr).

^{1}**H NMR** (400 MHz, $CDCl_3$): 4.59 (s, 2H), 3.44 (dd, *J* 9.3, 4.6, 1H), 3.34 (s, 3H), 3.19 (dd, *J* 9.3, 7.5, 1H), 1.80 (m, 1H), 1.62 (m, 1H), 1.38 (m, 1H), 1.23–1.20 (m, 25H), 1.15 (dt, *J* 9.9, 5.1, 1H), 1.01 (m, 1H), 0.93 (d, *J* 6.7, 3H), 0.89 (d, *J* 6.8, 3H), 0.70 (dd, *J* 15.7, 9.4, 1H).

^{13}**C NMR** (100 MHz, $CDCl_3$): 96.4, 82.7, 82.7, 73.2, 55.0 (CH_3), 40.5, 33.9, 30.9, 34.4, 24.9, 24.8, 24.7, 24.1, 18.6, 18.2, *carbons attached to boron not observed*.

IR (film): υ (cm^{-1}) 2977, 2928, 2878, 1370, 1309, 1141, 1047, 968, 846.

HRMS (ESI) calcd. for $C_{22}H_{44}B_2NaO_6$ $[M+Na]^+$ 449.3224, found 449.3231.

$[\alpha]_D^{22}$ = +3 (*c* 1, $CHCl_3$).

(*R*)-2,2'-(hex-5-yne-1,2-diyl)bis(4,4,5,5-tetramethyl-1,3,2-dioxaborolane) (10)

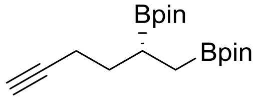

Following a modified version of **GP4** with benzoate **44** (0.25 mmol, 79 mg), *s*BuLi (0.42 mL, 1.3 M in hexanes, 2.2 eq.) and (+)-sparteine (0.13

mL, 0.55 mmol, 2.2 eq.) gave **10** as a colourless oil (50%, 41 mg (corrected for small amount of diethyl ether in NMR sample), ≥95:5 *er*). Enantiomeric ratio determined after oxidation and formation of the corresponding dimethyl acetal.

^{1}H NMR (400 MHz, $CHCl_3$): 2.19 (m, 2H), 1.90 (t, *J* 2.6, 1H), 1.72 (m, 1H), 1.56 (ddt, *J* 13.4, 8.5, 6.8, 1H), 1.22 (app. s, 24H), 0.92–0.73 (m, 3H).

^{13}C NMR (101 MHz, $CDCl_3$): 85.1, 82.9, 82.9, 67.8, 32.5, 24.9, 24.8, 24.8, 24.7, 17.7, *carbons attached to boron not observed.*

IR (film): υ (cm^{-1}) 3312, 2978, 2929, 2117, 1370, 1312, 1140, 968, 846, 626.

HRMS (ESI) calcd. for $C_{18}H_{32}B_2NaO_4$ $[M+Na]^+$ 357.2385, found 357.2393.

$[\alpha]_D^{22} = -1$ (*c* 1, $CHCl_3$).

Chiral GC: β-Dextrose column, 50 °C isocratic, 0.1 mL/min; t_R: 39.01 min (minor), 39.06 min (major), er ≥95:5.

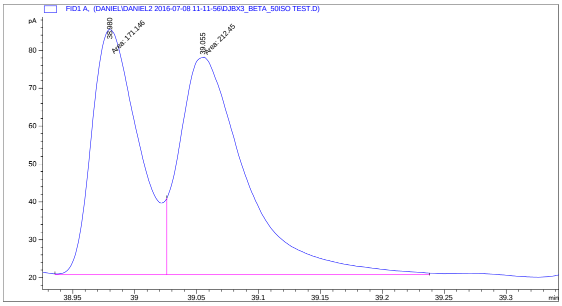

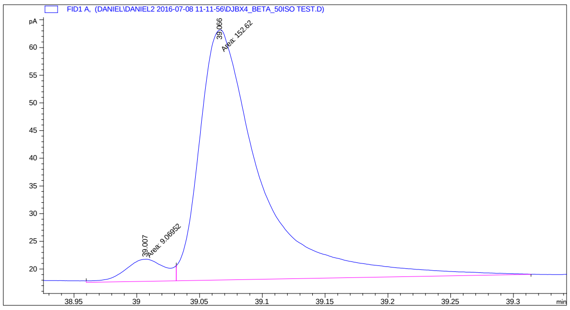

General procedure for single homologation of diborylmethane (1) using secondary benzylic carbamates (GP5)

To a vigorously stirred solution of secondary carbamate (1.5 eq.) and TMEDA (1.5 eq.) in anhydrous diethyl ether (0.33 M) at −78 °C under a nitrogen atmosphere, was added *s*BuLi (1.3 M in hexane, 1.45 eq.) dropwise. After 15 min, a solution of bis(4,4,5,5-tetramethyl-1,3,2-dioxaborolan-2-yl)methane (1.0 eq.) in diethyl ether (0.5 M) was added dropwise. The reaction mixture was stirred at −78 °C for 1 h and then a 1.0 M solution of $MgBr_2$ in anhydrous MeOH (1.7 eq.) was added slowly at –78 °C. After 5 min, the cooling bath was removed and stirring was continued at room temperature overnight (16 h). Afterwards the reaction mixture was diluted with water and extracted with Et_2O. The combined organic phases were washed with

brine and dried over $MgSO_4$. The solvent was removed *in vacuo* and the crude material purified by flash chromatography.

(*S*)-2,2'-(2-Phenylpropane-1,2-diyl)bis(4,4,5,5-tetramethyl-1,3,2-dioxaborolane) (11)

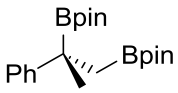

Following **GP5** using carbamate **52** (6.77 mmol, 1.69 g) without TMEDA gave **11** as a white solid (3.84 mmol, 1.43 g, 85%, 98:2 *er*).

m.p. = 88–91 °C (EtOH)

^{1}H NMR ($CDCl_3$, 400 MHz): 7.39 (2H, m), 7.25 (2H, m), 7.10 (1H, m), 1.49 (1H, d, *J* 15.7), 1.41 (3H, s), 1.21 (6H, s), 1.21 (6H, s), 1.20 (6H, s), 1.18 (6H, s), 1.15 (1H, d, *J* 15.7).

^{13}C NMR ($CDCl_3$, 101 MHz): 149.1, 127.9, 126.4, 124.8, 83.2, 82.9, 29.7, 25.1, 24.7, 24.5, 24.4, *carbons attached to boron not observed*.

IR (film): υ (cm^{-1}) 2981, 2928, 2872, 1471, 1379, 1353, 1313, 1137, 1111, 969, 837, 698.

HRMS (ESI): calcd. for $C_{21}H_{34}O_2BNa$ [M+Na]$^+$ 395.2541, found 395.2535.

Chiral HPLC: Chiralpak AS-H column with guard, 4% iPrOH in hexane, flow rate: 0.7 mL/min, 20 °C; t_R = 20.2 min (major), 26.2 min (minor). 98:2 er.

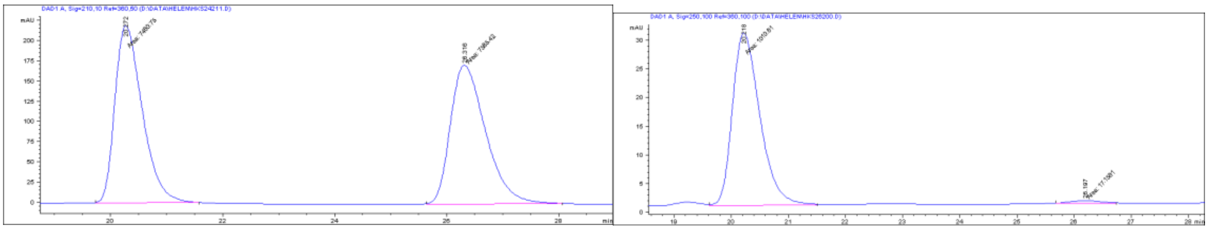

Data were consistent with those previously reported.[17]

(*S*)-2,2'-(2-((1,1'-biphenyl)-4-yl)propane-1,2-diyl)bis(4,4,5,5-tetramethyl-1,3,2 dioxaborolane) (12)

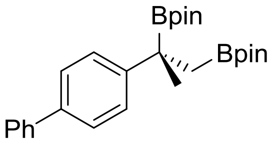

Following a modified version of **GP4** with MTBE as solvent, without TMEDA and performing the reaction at −96 °C using carbamate **53** (0.63 mmol, 205 mg, 98:2 er), **12** was obtained as a white solid (67%, 126 mg, 95:5 er).

^{1}H NMR (400 MHz, $CDCl_3$): 7.60 (ap d, *J* 8.3, 2H), 7.51–7.53 (m, 2H), 7.46–7.48 (m, 2H), 7.42 (ap t, *J* 7.6, 2H), 7.31 (ap t, *J* 7.6, 1H), 1.53 (d, *J* 15.7, 1H), 1.46 (s, 3H), 1.18–1.23 (m, 25H).

^{13}C NMR (100 MHz, $CDCl_3$): 148.4, 141.2, 137.5, 128.6, 126.9, 126.9, 126.7, 126.5, 83.3, 83.0, 25.1, 24.7, 24.7, 24.5, 24.4, *carbons attached to boron not observed*.

IR (film): υ (cm^{-1}) 2977, 2928, 1487, 1466, 1358, 1347, 1311, 1143

HRMS (ESI) calcd. for $C_{27}H_{38}B_2NaO_4$ $[M+Na]^+$ 471.2858, found 471.2846.

$[\alpha]_D^{22}$ = −22 (*c* 1, $CHCl_3$).

Chiral SFC: Whelk-01, 125 bar, 40°C, 4 mL/min, 10% co-solvent (50% IPA/Hexane); t_R: 11.87 min (minor), 17.54 min (major), *er* 95:5.

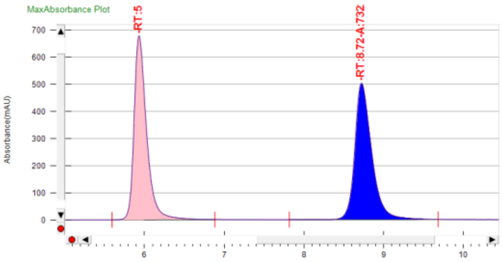

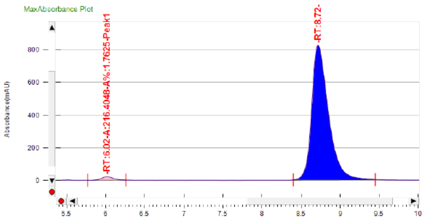

(*S*)-2,2'-(2-(4-chlorophenyl)propane-1,2-diyl)bis(4,4,5,5-tetramethyl-1,3,2-dioxaborolane) (13)

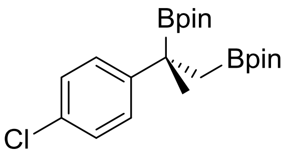

Following **GP5** with carbamate **54** (0.63 mmol, 179 mg, 99:1 er) and no TMEDA gave **13** as a white solid (73%, 125 mg, 98:2 er).

^{1}H NMR (400 MHz, $CDCl_3$): 7.31 (ap d, *J* 8.6, 2H), 7.21 (ap d, *J* 8.6, 2H), 1.42 (d, *J* 15.6, 1H), 1.37 (s, 3H), 1.17–1.24 (m, 24H), 1.11 (d, *J* 15.6, 1H).

^{13}C NMR (100 MHz, $CDCl_3$): 147.7, 130.5, 127.9, 127.8, 83.4, 83.0, 27.0, 25.0, 24.7, 24.6, 24.5, 24.4, 22.1.

IR (film): υ (cm^{-1}) 2978, 2928, 1490, 1471, 1379, 1342, 1313, 1136, 1008, 968, 838, 713.

HRMS (ESI) calcd. for $C_{21}H_{34}B_2ClO_4$ $[M+H]^+$, 407.2334, found 407.2329

$[\alpha]_D^{22}$ = −6 (*c* 1, $CHCl_3$).

Chiral SFC: Whelk-01, 125 bar, 40°C, 4 mL/min, 10% co-solvent (50% IPA/Hexane); t_R: 4.40 min (minor), 6.12 min (major), er 98:2.

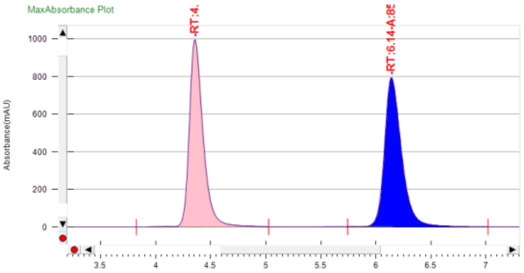

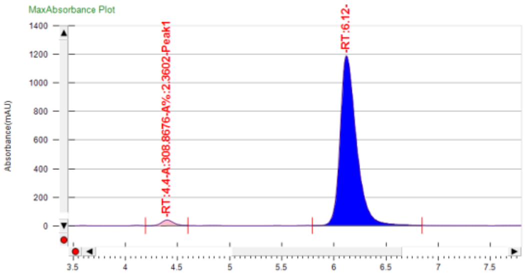

(*S*)-2,2'-(2-(*p*-tolyl)propane-1,2-diyl)bis(4,4,5,5-tetramethyl-1,3,2-dioxaborolane) (14)

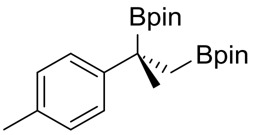

Following **GP5** with carbamate **55** (0.63 mmol, 166 mg, 99:1 *er*) gave **14** as an amorphous white solid (68%, 110 mg, 98:2 *er*).

^{1}H NMR (300 MHz, $CDCl_3$): 7.28 (d, *J* 8.2, 2H), 7.07 (d, *J* 8.2, 2H), 2.30 (s, 3H), 1.48 (d, *J* 15.6, 1H), 1.36 (s, 3H), 1.23 (s, 12H), 1.21 (s, 6H), 1.19 (s, 6H), 1.11 (d, *J* 15.6, 1H).

^{13}C NMR (76 MHz, $CDCl_3$): 146.3, 134.1, 128.7, 126.4, 83.3, 83.0, 26.8, 25.2, 24.9, 24.8, 24.6, 24.5, 22.0, 20.9.

IR (film): υ (cm^{-1}) 2977, 2927, 1511, 1462, 1348, 1308, 1142, 969, 845, 672.

HRMS (CI): calcd. for $C_{22}H_{36}B_2NaO_4$ $[M+Na]^+$ 409.2700, found 409.2713.

$[\alpha]_D^{22} = -13$ (*c* 1, $CHCl_3$).

Chiral SFC: Whelk-01, 125 bar, 42°C, 4 mL/min, 10% co-solvent (50% IPA/Hexane); t_R: 3.65 min (minor), 4.78 min (major), *er* 98:2.

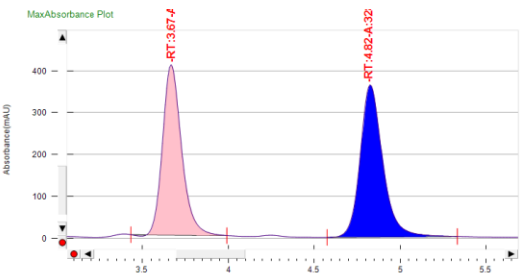

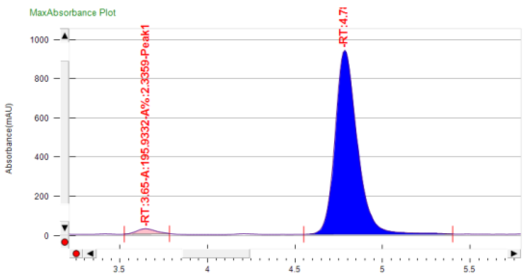

(*S*)-2,2'-(2-(4-methoxyphenyl)propane-1,2-diyl)bis(4,4,5,5-tetramethyl-1,3,2-dioxaborolane) (15)

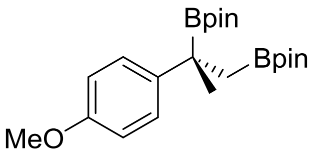

Following **GP5** with carbamate **56** (0.63 mmol, 176 mg, >99:1 er) gave **15** as a white solid (81%, 137 mg, 98:2 *er*).

^{1}H NMR (400 MHz, $CDCl_3$): 7.30 (app. d, *J* 8.8, 2H), 6.81 (app. d, *J* 8.8, 2H), 3.77 (s, 3H), 1.45 (d, *J* 15.5, 1H), 1.37 (s, 3H), 1.21 (s, 12H), 1.19 (s, 6H), 1.18 (s, 6H), 1.10 (d, *J* 15.5, 1H).

^{13}C NMR (100 MHz, $CDCl_3$): 156.9, 141.3, 127.3, 113.2, 83.2, 82.9, 55.1, 25.1, 24.9, 24.7, 24.5, 24.4, *carbons attached to boron not observed*.

IR (film): υ (cm^{-1}) 2977, 2931, 1614, 1510, 1378, 1349, 1308, 1247, 1142.

HRMS (ESI) calcd. for $C_{22}H_{36}B_2NaO_5$ $[M+Na]^+$ 425.2649, found 425.2646.

$[\alpha]_D^{22} = -9$ (*c* 1, $CHCl_3$).

Chiral SFC: Whelk-01, 125 bar, 42°C, 4 mL/min, 10% co-solvent (50% IPA/Hexane); t_R: 6.02 min (minor), 8.72 min (major), er 98:2.

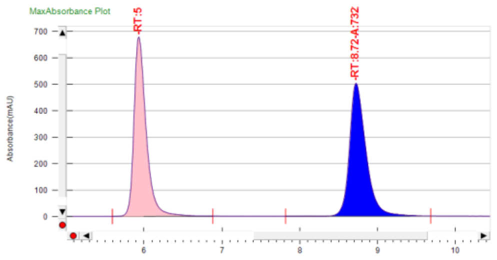

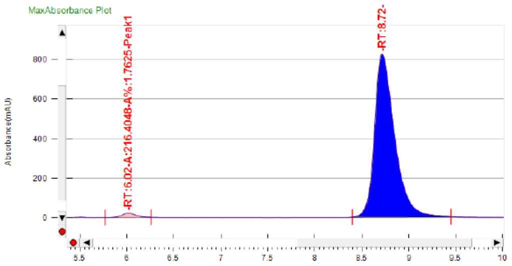

(*S*)-2,2'-(2-(4-fluorophenyl)propane-1,2-diyl)bis(4,4,5,5-tetramethyl-1,3,2-dioxaborolane) (16)

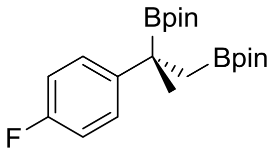

Following **GP5** with carbamate **57** (0.63 mmol, 168 mg, >99:1 er) and no TMEDA gave **16** as a white solid (75%, 123 mg, >99:1 er).

^{1}H NMR (400 MHz, $CDCl_3$): 7.33 (app. dd, *J* 8.8, 5.6, 2H), 6.93 (app. t, *J* 8.8, 2H), 1.45 (d, *J* 15.6, 1H), 1.38 (s, 3H), 1.19 (s, 18H), 1.17 (s, 6H), 1.13 (d, *J* 15.6, 1H).

^{13}C NMR (100 MHz, $CDCl_3$): 159.6 (d, $^{1}J_{C\text{-}F}$ 242.4), 143.7, 126.9 (d, $^{3}J_{C\text{-}F}$ 7.6), 113.4 (d, $^{2}J_{C\text{-}F}$ 20.7), 82.3, 82.0, 24.0, 23.8, 23.7, 23.5, 23.4 *carbons attached to boron not observed.*

^{19}F NMR (376 MHz; $CDCl_3$): 119.5 (m)

IR (film): υ (cm^{-1}) 2978, 2931, 1605, 1508, 1371, 1314, 1143, 968, 846.

HRMS (ESI) calcd. for $C_{21}H_{34}B_2FO_4$ [M+H]$^+$ 391.2629, found 391.2632.

$[\alpha]_D^{22} = -6$ (c 1, $CHCl_3$).

Chiral SFC: Whelk-01, 125 bar, 40°C, 4 mL/min, 10% co-solvent (50% IPA/Hexane); t_R: 3.02 min (major), 3.68 min (minor), er > 99:1.

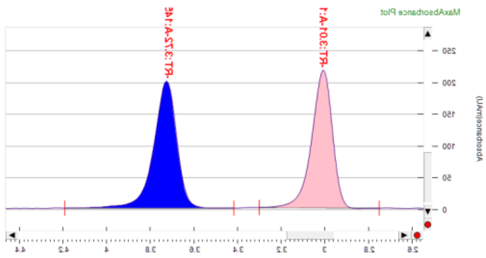

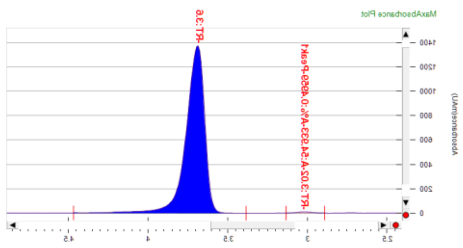

(*S*)-2,2'-(2-(2-fluorophenyl)propane-1,2-diyl)bis(4,4,5,5-tetramethyl-1,3,2-dioxaborolane) (17)

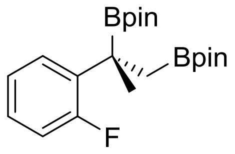

Following **GP5** with carbamate **58** (0.63 mmol, 168 mg, 99:1 er) and no TMEDA gave **17** as a white solid (46%, 75 mg, 99:1 er).

^{1}H NMR (400 MHz, $CDCl_3$): 7.34 (ap td, J 7.9, 1.9, 1H), 7.03–7.13 (m, 2H), 6.94 (m, 1H), 1.42 (s, 3H), 1.29–1.26 (m, 2H), 1.24 (s, 12H), 1.17 (bs, 6H), 1.15 (s, 6H).

^{13}C NMR (100 MHz, $CDCl_3$): 161.1 (d, $^{1}J_{C\text{-}F}$ 243.9), 136.6 (d, $^{2}J_{C\text{-}F}$ 14.1), 127.1 (d, $J_{C\text{-}F}$ 5.4), 126.6 (d, $J_{C\text{-}F}$ 8.6), 123.8 (d, $J_{C\text{-}F}$ 3.2), 114.8 (d, $J_{C\text{-}F}$ 23.1), 83.4, 82.8, 24.9, 24.7, 24.7, 24.6, 23.2, *carbons attached to boron not observed*.

IR (film): υ (cm^{-1}) 2977, 2931, 1449, 1371, 1352, 1144, 968, 845, 754

^{19}F NMR (376 MHz; $CDCl_3$): 112.0 (dt, J 11.4, 5.6)

HRMS (ESI) calcd. for $C_{21}H_{33}B_2FNaO_4$ [M+Na]$^+$ 413.2449, found 413.2451.

$[\alpha]_D^{22} = -11$ (c 1, $CHCl_3$).

Chiral SFC: Chiralcel IA , 125 bar, 42°C, 4 mL/min, 10% co-solvent (50% IPA/Hexane); t_R: 5.73 min (major), 8.00 min (minor), er 99:1.

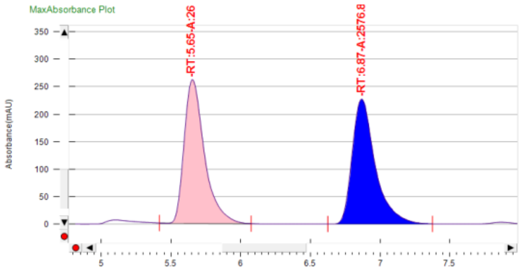

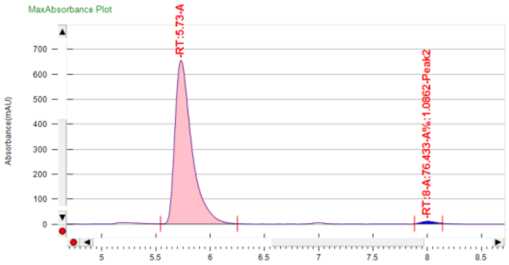

(*S*)-2,2'-(2-(2-methoxyphenyl)propane-1,2-diyl)bis(4,4,5,5-tetramethyl-1,3,2-dioxaborolane) (18)

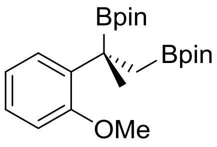

Following **GP5** with carbamate **59** (0.63 mmol, 176 mg, >99:1 er) gave **18** as a white solid (45%, 76 mg, >99:1 er).

^{1}H NMR (400 MHz, $CDCl_3$): 7.29 (dd, *J* 7.7, 1.6, 1H), 7.11 (m, 1H), 6.89 (m, 1H), 6.77 (dd, *J* 8.1, 1.2, 1H), 3.79 (s, 3H), 1.41 (s, 3H), 1.29 (d, *J* 14.7, 1H), 1.24–1.18 (s, 13H), 1.16 (s, 6H), 1.13 (s, 6H).

^{13}C NMR (100 MHz, $CDCl_3$): 156.6, 138.0, 126.1, 125.9, 120.5, 109.6, 82.8, 82.5, 54.8, 24.9, 24.8, 24.8, 24.7, 22.6, *carbons attached to boron not observed*.

IR (film): υ (cm^{-1}) 2976, 2931, 1607, 1615, 1488, 1463, 1347, 1323, 1239, 1144.

HRMS (ESI) calcd. for $C_{22}H_{37}B_2O_5$ $[M+H]^+$ 403.2830, found 403.2844.

$[\alpha]_D^{22}$ = –27 (*c* 1, $CHCl_3$).

Chiral SFC: Whelk-01, 125 bar, 40°C, 4 mL/min, 10% co-solvent (50% IPA/Hexane); t_R: 4.37 min (minor), 5.41 min (major), er >99:1.

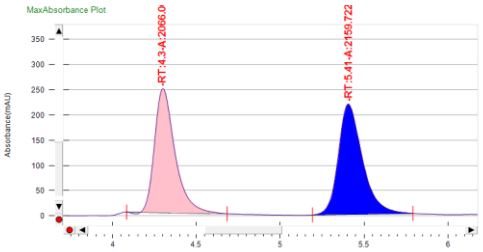

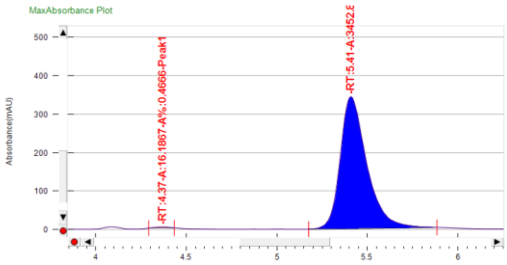

Homologation of a dialkyl carbenoid

(*R*)-2,2'-(2-methyl-4-phenylbutane-1,2-diyl)bis(4,4,5,5-tetramethyl-1,3,2-dioxaborolane) (20)

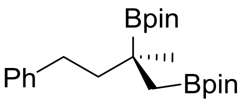

*n*BuLi (0.18 mL, 0.28 mmol, 1.1 eq.) was added dropwise to a solution of (*S*)-4-phenyl-2-(trimethylstannyl)butan-2-yl 2,4,6-triisopropylbenzoate (**19**)[18] (135 mg, 0.25 mmol, 1 eq.) and TMEDA (0.04 mL, 0.28 mmol, 1.1 eq.) in Et_2O (1 mL) at –78 °C. After 2 h at this temperature an ethereal solution of bis(4,4,5,5-tetramethyl-1,3,2-dioxaborolan-2-yl)methane (101 mg, 1.5 mmol, 1.5 eq.) was added. After 2 h the reaction mixture was warmed to room temperature then heated at reflux overnight. The reaction mixture was quenched through addition of water (5 mL) and the organic layer separated. The aqueous phase was washed with Et_2O (2 × 5 mL), the organics were combined, dried over $MgSO_4$, filtered and concentrated *in vacuo*. Purification of the residue by column chromatography gave **20** (62 mg, 62%).

^{1}H NMR (400 MHz, $CDCl_3$): 7.26 (m, 2H), 7.19–7.11 (m, 3H), 2.56 (m, 2H), 1.63 (m, 2H), 1.27 (s, 12H), 1.24 (s, 12H), 1.06 (s, 3H), 1.04 (d, *J* 10.6, 1H), 0.77 (d, *J* 15.6, 1H).

^{13}C NMR (100 MHz, $CDCl_3$): 143.8, 128.4, 128.2, 125.3, 83.0, 82.8, 44.2, 32.4, 25.0, 24.9, 24.8, 24.7, 23.9, *carbons attached to boron not observed.*

IR (film): υ (cm^{-1}) 2977, 2928, 2862, 1470, 1370, 1309, 1143, 969, 846, 698.

HRMS (ESI) calcd. for $C_{23}H_{38}B_2NaO_4$ $[M+Na]^+$ 423.2857, found 423.2869.

$[\alpha]_D^{22} = -3$ (*c* 1, $CHCl_3$).

Chiral HPLC (Chiralpak IB, 90:10 hexane:IPA, 1 ml/min, RT) t_R: 10.18 min (major), 14.99 min (minor), *er* 95:5

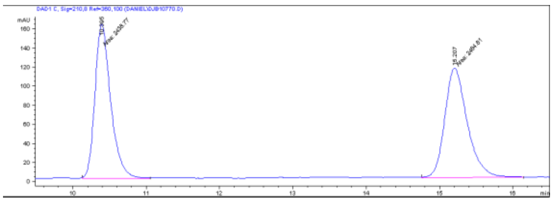

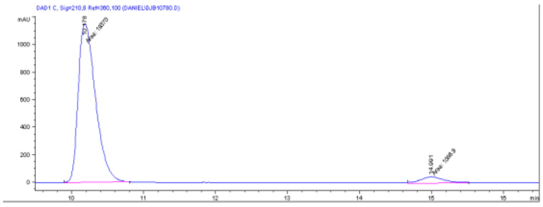

General Procedure for Symmetrical Double Homologation of Diborylmethane (GP6)

*n*BuLi (0.25 mL, 0.40 mmol, 2.00 eq., 1.6 M in hexanes) was added dropwise to a solution of stannane (0.41 mmol, 2.05 eq.) in Et_2O (2.05 mL) at −78 °C. After 1 h at this temperature a solution of bis(4,4,5,5-tetramethyl-1,3,2-dioxaborolan-2-yl)methane (0.2 mmol, 1.0 eq.) in diethyl ether (0.4 mL) was added dropwise. The reaction mixture was warmed to RT after 2 h and then stirred overnight. The reaction mixture was quenched with water (10 mL) and the organic layer separated. The aqueous phase was washed with Et_2O (3 x 10 mL), the organics were combined, dried ($MgSO_4$), filtered and concentrated *in vacuo*. Purification of the residue by column chromatography (3–10% Et_2O/pentane) afford C_2-symmetric 1,3-bis(boronic ester).

2,2'-((3*S*,5*S*)-1,7-bis(4-methoxyphenyl)heptane-3,5-diyl)bis(4,4,5,5-tetramethyl-1,3,2-dioxaborolane) (4)

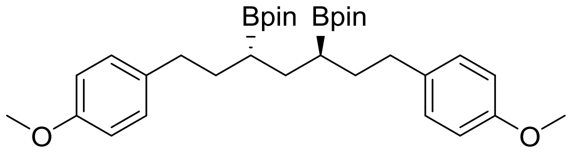

Following modified version of **GP6** using stannane **46** (185 mg, 0.27 mmol, 1.1 eq.), *n*BuLi (0.16 mL, 0.25 mmol, 1 eq.) and diborylmethane **1** (100 mg, 0.37 mmol, 1.5 eq.) gave 1,3-bis(boronic ester) **4** (92 %, 65 mg).

^{1}H NMR (400 MHz, $CDCl_3$): 7.11–7.04 (m, 4H), 6.82–6.77 (m, 4H), 3.77 (s, 6H), 2.65–2.43 (m, 4H), 1.70–1.57 (m, 4H), 1.52 (t, *J* 8.0, 2H), 1.24–1.21 (m, 24H), 1.05 (m, 2H).

^{13}C NMR (100 MHz, $CDCl_3$): 155.6, 133.3, 127.4, 111.7, 80.9, 53.3, 32.7, 32.4, 31.1, 22.9 (overlapping peaks), carbons attached to boron not observed.

IR (film): υ (cm^{-1}) 2981, 2928, 2855, 1612, 1586, 1511, 1379, 1370, 1241, 1141, 1040, 824.

HRMS (ESI) calcd. for $C_{33}H_{50}B_2NaO_6$ $[M+Na]^+$ 587.3697, found 587.3686.

$[\alpha]_D^{22} = -8$ (*c* 1, $CHCl_3$).

2,2'-((2*S*,4*S*)-pentane-2,4-diyl)bis(4,4,5,5-tetramethyl-1,3,2-dioxaborolane) (21)

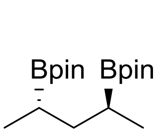

According to **GP6** using stannane *ent*-**47** (180 mg, 0.41 mmol) gave 1,3-bis(boronic ester) **21** (71%, 46 mg)

^{1}H NMR (300 MHz, $CDCl_3$): 1.41 (t, *J* 7.7, 2H), 1.22 (s, 24H), 1.06 (sextet, *J* 7.4, 2H), 0.92 (d, *J* 7.2, 2H).

^{13}C NMR (100 MHz, $CDCl_3$): 82.7, 36.0, 24.7, 15.4 *carbon attached to boron not observed.*

IR (film): υ (cm^{-1}) 2978, 2951, 2931, 2871, 1460, 1379, 1370, 1310, 1142, 968, 861, 685.

HRMS (ESI) calcd. for $C_{17}H_{34}B_2NaO_4$ [*M*+Na]$^+$ 347.2542, found 347.2549.

$[\alpha]_D^{22}$ = +13 (*c* 2, $CHCl_3$).

2,2'-((3*R*,5*R*)-2,6-dimethylheptane-3,5-diyl)bis(4,4,5,5-tetramethyl-1,3,2-dioxaborolane) (22)

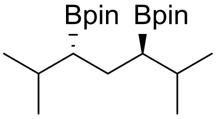

According to **GP6** using stannane **51** (192 mg, 0.41 mmol) gave 1,3-bis(boronic ester) **22** (88%, 68 mg).

^{1}H NMR (400 MHz, $CDCl_3$): 1.68 (app. octet, *J* 6.7, 1H), 1.47 (dd, *J* 9.1, 6.6, 2H), 1.24 (s, 12H), 1.23 (s, 12H), 0.92 (d, *J* 6.3, 6H), 0.91 (d, *J* 6.5, 6H), 0.76 (dt, *J* 8.8, 6.3, 2H).

^{13}C NMR (100 MHz, $CDCl_3$): 80.8, 28.6, 26.7, 23.2, 23.2, 22.9, 22.9, 20.5, 19.8.

IR (film): υ (cm^{-1}) 2977, 2955, 2935, 1470, 1378, 1371, 1307, 1143, 973.

HRMS (ESI) calcd. for $C_{21}H_{42}B_2NaO_4$ [*M*+Na]$^+$ 403.3169, found 403.3168.

$[\alpha]_D^{22}$ = +19 (*c* 1, $CHCl_3$).

2,2'-((5*S*,7*S*)-undeca-1,10-diene-5,7-diyl)bis(4,4,5,5-tetramethyl-1,3,2-dioxaborolane) (23)

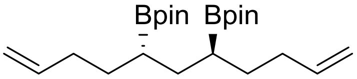

According to **GP6** using stannane **50** (197 mg, 0.41 mmol) gave 1,3-bis(boronic ester) **23** (64%, 52 mg).

^{1}H NMR (400 MHz, $CDCl_3$): 5.80 (ddt, *J* 16.9, 10.2, 6.6, 2H), 4.98 (dq, *J* 17.1, 1.8, 2H), 4.90 (ddt, *J* 10.2, 2.3, 1.2, 2H), 2.10–1.96 (m, 4H), 1.52–1.36 (m, 4H), 1.24–1.21 (s, 24H), 1.01 (app. p, *J* 7.9, 2H).

^{13}C NMR (100 MHz, $CDCl_3$): 139.3, 114.1, 82.8, 33.4, 32.9, 31.3, 24.8, 24.8.

IR (film): υ (cm^{-1}) 2978, 2922, 2852, 1646, 1379, 1371, 1312, 1142, 967, 907.

HRMS (ESI) calcd. for $C_{23}H_{42}B_2NaO_4$ $[M+Na]^+$ 427.3170, found 427.3166.

$[\alpha]_D^{22}$ = +5 (*c* 1, $CHCl_3$).

(6*R*,8*R*)-2,2,12,12-tetramethyl-6,8-bis(4,4,5,5-tetramethyl-1,3,2-dioxaborolan-2-yl)-3,11-dioxa-2,12-disilatridecane (24)

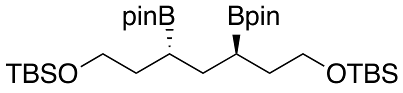

According to **GP6** using stannane **49** (150 mg, 0.26 mmol) gave 1,3-bis(boronic ester) **24** (55 %, 42.1 mg).

^{1}H NMR (400 MHz, $CDCl_3$): 3.63 – 3.51 (m, 4H), 1.62 – 1.56 (m, 4H), 1.47 – 1.43 (m, 2H), 1.22 (s, 24H), 1.02 – 0.98 (m, 2H) 0.88 (s, 18H), 0.03 (s, 12H)

^{13}C NMR (101 MHz, $CDCl_3$): 82.8, 63.2, 35.0, 33.0, 26.0, 24.8, 18.4, −5.2 *carbon attached to boron not observed*.

$[\alpha]_D^{22}$ = – 2 (*c* = 1.0, $CHCl_3$)

IR (film): υ (cm^{-1}) 2928, 2857, 1463, 1371, 1311, 1251, 1142, 1092, 834.

HRMS (ESI) calc. $C_{31}H_{66}B_2NaO_6Si_2$$[M+Na]^+$ 635.4488, found 635.4466.

2,2'-((2*S*,4*S*)-2,4-bis(4-methoxyphenyl)pentane-2,4-diyl)bis(4,4,5,5-tetramethyl-1,3,2-dioxaborolane) (25)

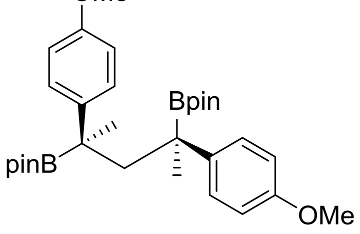

Carbamate **56** (112 mg, 0.4 mmol, 2.0 eq., >99:1 *er*) and TMEDA (2.0 eq.) were dissolved in anhydrous TBME (0.33 M) and cooled to −78 °C. *s*BuLi (1.3 M in hexane, 2.2 eq.) was added dropwise and after 15 min, a solution of **15** (80 mg, 0.2 mmol, 1.0 eq., 98:2 *er*) in TBME (0.5 M) was added dropwise. The reaction mixture was stirred at −78 °C for 1 h and then stirred overnight at room temperature. The reaction mixture was then diluted with water and extracted with Et_2O. The combined organic phases were washed with brine and dried over $MgSO_4$. The solvent was removed in vacuo and the

crude material purified by flash chromatography affording to **25** (32 %, 34 mg, >20:1 *dr*) as a white solid.

^{1}H NMR (400 MHz, $CDCl_3$): 7.36 (app. d, *J* 8.7, 4H), 6.80 (app. d, *J* 8.7, 4H), 3.77 (s, 6H), 2.34 (s, 2H), 1.43 (s, 6H), 1.10 (s, 12H), 1.06 (s, 12H).

^{13}C NMR (100 MHz, $CDCl_3$): 157.0, 140.0, 127.8, 113.2, 83.3, 55.2, 47.9, 24.7, 24.3, 20.5, *carbon attached to boron not observed*.

IR (film): υ (cm^{-1}) 2973, 2903, 1509, 1467, 1379, 1307, 1248, 1186, 1129, 1057, 840.

HRMS (ESI) calcd. for $C_{31}H_{46}B_2NaO_6$ $[M+Na]^+$ 559.3383, found 559.3369.

$[\alpha]_D^{22} = -2$ (*c* 1, $CHCl_3$).

2,2'-((2*S*,4*R*)-2-(4-methoxyphenyl)pentane-2,4-diyl)bis(4,4,5,5-tetramethyl-1,3,2-dioxaborolane) (26)

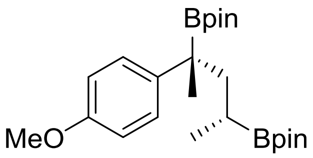

Stannane *ent*-**47** (79 mg, 0.18 mmol, 1.0 eq.) was dissolved in anhydrous Et_2O (0.2 M) under an atmosphere of nitrogen in the receiving section of the Fawcett Flask.[19] The reaction mixture was cooled to –78 °C and *n*BuLi (1.6 M in hexanes, 1.0 eq.) was added dropwise. After 1 h at –78 °C **15** (80 mg, 0.2 mmol, 98:2 er, 0.5 M in anhydrous Et_2O, 1.1 eq.) was tipped from the delivering side of the Fawcett Flask to the solution of carbenoid and the resulting mixture was stirred at –78 °C for 30 min. The reaction mixture was stirred for a further 4 h at room temperature and was then diluted with water and extracted with Et_2O. The combined organic phases were washed with brine and dried over $MgSO_4$. The solvent was removed in *vacuo* and the crude material purified by flash chromatography to afford **26** (59 %, 46 mg, >20:1 *dr*) as a white solid.

^{1}H NMR (400 MHz, $CDCl_3$): 7.22 (app. d, *J* 8.8, 2H), 6.79 (app. d, *J* 8.8, 2H), 3.76 (s, 3H), 2.01 (dd, *J* 13.8, 8.0, 1H), 1.79 (dd, *J* 13.8, 3.6, 1H), 1.30 (s, 3H), 1.19 (s, 12H), 1.17 (s, 6H), 1.16 (s, 6H), 0.85 (d, *J* 7.2, 3H), 0.77 (m, 1H).

^{13}C NMR (100 MHz, $CDCl_3$): 156.9, 138.3, 128.1, 113.3, 83.0, 82.6, 55.1, 42.0, 24.7, 24.6, 24.6, 24.5, 21.4, 17.8, *carbons attached to boron not observed*.

IR (film): υ (cm^{-1}) 2976, 2901, 1510, 1406, 1380, 1308, 1249, 1145, 1066, 1057.

HRMS (ESI) calcd. for $C_{24}H_{40}B_2NaO_5$ [*M*+Na]$^+$ 453.2963, found 453.2952.

$[\alpha]_D^{22} = -12$ (*c* 1, $CHCl_3$).

2,2'-((2*R*,3*S*,5*R*)-3-(4-methoxyphenyl)-3-methylhexane-2,5-diyl)bis(4,4,5,5-tetramethyl-1,3,2-dioxaborolane) (27)

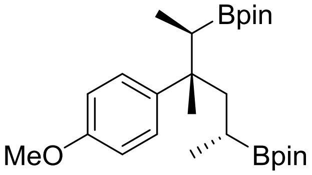

Stannane *ent*-**47** (194 mg, 0.44 mmol, 2.2 eq.) was dissolved in anhydrous Et_2O (0.2 M) under an atmosphere of nitrogen. The reaction mixture was cooled to –78 °C and *n*BuLi (1.6 M in hexanes, 2.1 eq.) was added dropwise. After 1 h, **15** (80 mg, 0.2 mmol, 98:2 er, 0.5 M in anhydrous Et_2O, 1.0 eq.) was added dropwise to the reaction mixture at –78 °C. The reaction mixture was stirred at – 78 °C for 30 min, then was removed from the cooling bath and stirred at room temperature for 4 h. The reaction mixture was diluted with water and extracted with Et_2O. The combined organic phases were washed with brine and dried over $MgSO_4$. The solvent was removed in vacuo and the crude material purified by flash chromatography to afford **27** (52 %, 48 mg, >20:1 dr).

^{1}H NMR (400 MHz, $CDCl_3$): 7.18 (app. d, *J* 8.8, 2H), 6.98 (app. d, *J* 8.8, 2H), 3.77 (s, 3H), 2.02 (dd, *J* 13.5, 8.8, 1H), 1.67 (dd, *J* 13.5, 2.6, 1H), 1.42 (q, *J* 7.4, 1H), 1.28 (s, 6H), 1.28 (s, 6H), 1.26 (s, 3H, partially overlapped with q, 1H), 1.19 (s, 6H), 1.18 (s, 6H), 0.71 (d, *J* 7.5, 3H), 0.58 (d, *J* 7.4, 3H).

^{13}C NMR (100 MHz, $CDCl_3$): 157.0, 139.9, 127.7, 112.9, 82.8, 82.6, 55.1, 47.4, 43.2, 24.9, 24.8, 24.7, 24.6, 19.9, 17.8, 11.0, *carbons attached to boron not observed*.

IR (film): υ (cm^{-1}) 2976, 2930, 1512, 1379, 1313, 1249, 1146, 1042.

HRMS (ESI) calcd. for $C_{26}H_{44}B_2NaO_5$ [*M*+Na]$^+$ 481.3276, found 481.3295.

$[\alpha]_D^{22} = -16$ (*c* 1, $CHCl_3$).

General Procedure for Mixed Double Homologation of Diborylmethane 1 (GP7)

*s*BuLi (0.23 mL, 0.3 mmol, 1 eq.) was added dropwise to a solution of 3-(4-methoxyphenyl)propyl *N*,*N*-diisopropylcarbamate[8] (**28**, 88 mg, 0.3 mmol, 1 eq.) and (+)-sparteine (0.07 ml, 0.3 mmol, 1 eq.) in Et_2O (1.5 mL) at −78 °C. After 3 h a solution of bis(4,4,5,5-tetramethyl-1,3,2-dioxaborolan-2-yl)methane (80 mg, 0.3 mmol, 1 eq.) in Et_2O (0.3 mL) was added dropwise and stirred for 2 h at −78 °C. A solution of stannane (0.45 mmol, 1.5 eq.) in Et_2O (2.25 mL) was added dropwise, followed by slow addition (~1 drop / 20 seconds) of *n*BuLi (0.26 mL, 1.6 M in hexanes, 1.45 eq.) and the reaction mixture stirred for 1 h at −78 °C. The reaction mixture was warmed to room temperature, heated at reflux overnight and quenched through addition of water. The organic layer was separated and the aqueous layer washed with Et_2O. The organics were combined, dried over $MgSO_4$, filtered and concentrated *in vacuo*. Purification of the residue by column chromatography (SiO_2) eluting with 10% Et_2O in pentane gave 1,3-bis(boronic ester).

2,2'-((2*S*,4*S*)-6-(4-methoxyphenyl)hexane-2,4-diyl)bis(4,4,5,5-tetramethyl-1,3,2-dioxaborolane) (31)

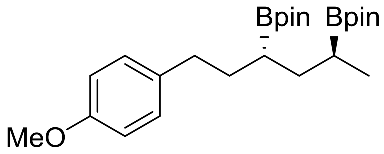

Following **GP7** using stannane *ent*-**47** (198 mg) gave 1,3-bis(boronic ester) **31** (60%, 80 mg).

^{1}H NMR (400 MHz, $CDCl_3$): 7.10 (app. d, *J* 8.6, 2H), 6.81 (app. d, *J* 8.6, 2H), 3.78 (s, 3H), 2.63–2.45 (m, 2H), 1.73–1.59 (m, 2H), 1.55 (ddd, *J* 13.1, 8.8, 6.7, 1H), 1.38 (m, 1H), 1.26 (s, 12H), 1.22 (s, 6H), 1.21 (s, 6H), 1.12–1.00 (m, 2H), 0.95 (d, *J* 7.2, 3H).

^{13}C NMR (100 MHz, $CDCl_3$): 157.5, 135.3, 129.2, 113.6, 82.8, 82.7, 55.2, 34.7, 34.6, 34.0, 24.9 (×2), 24.8 (×2), 24.7 (×4), 16.0, *carbons attached to boron not observed.*

IR (film): υ (cm^{-1}) 2977, 2928, 1512, 1380, 1371, 1314, 1246, 1143, 1039, 862.

HRMS (ESI) calcd. for $C_{25}H_{42}B_2NaO_5$ [*M*+Na]$^+$ 467.3119, found 467.3124.

$[\alpha]_D^{22}$ = −2 (*c* 1, $CHCl_3$).

2,2'-((3*S*,5*S*)-1-(4-methoxyphenyl)-7-phenylheptane-3,5-diyl)bis(4,4,5,5-tetramethyl-1,3,2-dioxaborolane) (32)

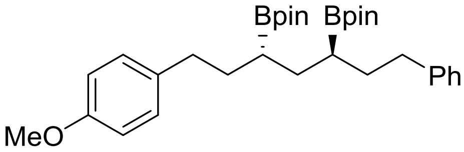

Following **GP7** using stannane **48** (239 mg) gave 1,3-bis(boronic ester) **32** (53%, 84 mg).

^{1}H NMR (400 MHz, $CDCl_3$): 7.28–7.21 (m, 2H), 7.19–7.13 (m, 3H), 7.11 – 7.06 (m, 2H), 6.83–6.77 (m, 2H), 3.77 (s, 3H), 2.68–2.45 (m, 4H), 1.77–1.57 (m, 4H), 1.53 (app. t, *J* 8.0, 2H), 1.24–1.22 (s, 24H), 1.12–0.99 (m, 2H).

^{13}C NMR (100 MHz, $CDCl_3$): 157.5, 143.1, 135.3, 129.3, 128.4, 128.1, 125.4, 113.6, 82.8, 82.8, 55.2, 35.6, 34.6, 34.3, 34.1, 33.0 , 24.8 (×4) *carbons attached to boron not observed.*

IR (film): υ (cm^{-1}) 2977, 2924, 2862, 1612, 1589, 1512, 1379, 1371, 1314, 1245, 1142, 699.

HRMS (ESI) calcd. for $C_{32}H_{48}B_2NaO_5$ $[M+Na]^+$ 557.3591, found 557.3583.

$[\alpha]_D^{21}$ = −7 (*c* 1, $CHCl_3$).

***tert*-butyl(((3*R*,5*S*)-7-(4-methoxyphenyl)-3,5-bis(4,4,5,5-tetramethyl-1,3,2-dioxaborolan-2-yl)heptyl)oxy)dimethylsilane (33)**

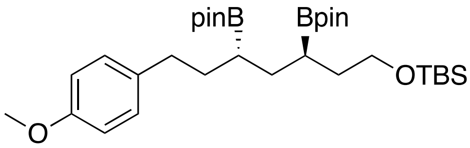

Using **GP7** and stannane **49** (0.263 g, 0.45 mmol) gave 1,3-bis(boronic ester) **33** (0.043 g, 24 %).

^{1}H NMR (400 MHz, $CDCl_3$): 7.09 (d, *J* 8.5, 2H), 6.79 (d, *J* 8.5, 2H), 3.77 (s, 3H), 3.58 (m, 2H), 2.57 – 2.49 (m, 2H), 1.64 – 1.56 (m, 4H), 1.48 (t, *J* 8, 2H), 1.24 (s, 12H), 1.2 (s, 12H), 1.10-0.95 (m, 2H), 0.88 (s, 9H), 0.03 (s, 6H).

^{13}C NMR (101 MHz, $CDCl_3$): 157.5, 135.3, 130.0, 113.6, 82.8, 82.7, 63.2, 55.2, 35.2, 34.6, 34.3, 33.1, 26.0, 24.9, 24.8, 18.4, −5.2, *carbons attached to boron not observed.*

$[\alpha]_D^{22}$ = – 5 (*c* = 1.0, $CHCl_3$)

IR (film): υ (cm^{-1}) 2977, 2928, 2856, 1371, 1312.

HRMS (ESI) calc. $C_{32}H_{58}B_2NaO_6Si$ $[M+Na]^+$ 611.4093, found 611.4080.

2,2'-((3*S*,5*S*)-1-(4-methoxyphenyl)non-8-ene-3,5-diyl)bis(4,4,5,5-tetramethyl-1,3,2-dioxaborolane) (34)

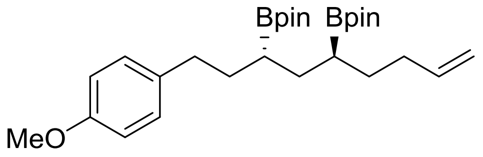

Following **GP7** using stannane **50** (216 mg) gave 1,3-bis(boronic ester) **34** (74 mg, 51%)

^{1}H NMR (400 MHz, $CDCl_3$): 7.09 (m, 2H), 6.79 (m, 2H), 5.80 (ddt, *J* 16.9, 10.1, 6.7, 1H), 4.98 (app. dq, *J* 17.1, 1.7, 1H), 4.90 (ddt, *J* 10.1, 2.2, 1.2, 1H), 3.77 (s, 3H), 2.53 (m, 2H), 2.04 (m, 2H), 1.72–1.54 (m, 3H), 1.53–1.35 (m, 3H), 1.26–1.23 (s, 12H), 1.21–1.19 (s, 12H), 1.10–0.96 (m, 2H).

^{13}C NMR (100 MHz, $CDCl_3$): 157.5, 139.3, 135.3, 129.3, 114.1, 113.6, 82.8, 82.8, 55.3, 34.6, 34.4, 33.4, 33.0, 31.3, 24.9, 24.8, *carbons attached to boron not observed*.

IR (film): υ (cm^{-1}) 2977, 2922, 2858, 16423, 1615, 1586, 1512, 1379, 1314, 1245, 1142, 1039, 967, 862.

HRMS (ESI) calcd. for $C_{28}H_{46}B_2O_5Na$ [*M*+Na]$^+$ 507.3433, found 507.3414.

$[\alpha]_D^{21} = -3$ (*c* 1, $CHCl_3$).

2,2'-((2*R*,4*S*)-6-(4-methoxyphenyl)hexane-2,4-diyl)bis(4,4,5,5-tetramethyl-1,3,2-dioxaborolane) (35)

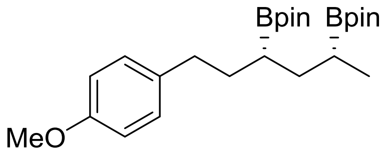

Following **GP7** using stannane **47** (198 mg) gave 1,3-bis(boronic ester) **35** (57%, 77 mg).

^{1}H NMR (400 MHz, $CDCl_3$): 7.09 (d, *J* 8.6, 2H), 6.80 (d, *J* 8.6, 2H), 3.77 (s, 3H), 2.64–2.41 (m, 2H), 1.75–1.55 (m, 3H), 1.36 (ddd, *J* 13.6, 8.6, 6.8, 1H), 1.25 (s, 12H), 1.20 (s, 12H), 1.16 (m, 1H), 1.07 (m, 1H), 0.95 (d, *J* 7.3, 3H).

^{13}C NMR (100 MHz, $CDCl_3$): 157.5, 135.3, 129.2, 113.6, 82.8, 82.7, 55.3, 34.7, 33.7, 33.6, 24.9, 24.8, 24.7, 24.7, 15.2.

IR (film): υ (cm^{-1}) 2977, 2931, 2858, 1512, 1379, 1370, 1313, 1245, 1143, 1038.

HRMS (ESI) calcd. for $C_{25}H_{42}B_2NaO_5$ $[M+Na]^+$ 467.3119, found 467.3101.

$[\alpha]_D^{22} = -1$ (*c* 1, $CHCl_3$).

C-B Functionalisation Reactions

(*R*)-2,2'-(2-(4-methoxyphenyl)-2-methylbutane-1,4-diyl)bis(4,4,5,5-tetramethyl-1,3,2-dioxaborolane) (36)

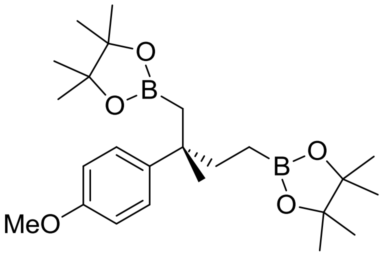

A solution of 1,2-bis(boronic ester) **15** (80 mg, 0.2 mmol, 1.0 equiv) and bromochloromethane (155 mg, 78 µL, 1.2 mmol, 6.0 equiv) was dissolved in anhydrous Et_2O (0.2 M) under an atmosphere of nitrogen. The reaction mixture was cooled to –78 °C. *n*BuLi (1.6 M in hexanes, 5 equiv) was added dropwise to the reaction mixture at –78 °C. The reaction mixture was stirred for 20 min at –78 °C. The reaction mixture was removed from the cooling bath and stirred at room temperature for 1 h. Afterwards, the reaction mixture was diluted with water and extracted with Et_2O (3 ×). The combined organic phases were washed with brine and dried over $MgSO_4$. The solvent was removed in vacuo and the crude material purified by flash chromatography to afford double homologation product **36** (83%, 71 mg).

$\boldsymbol{R_f}$ = 0.16 (Pentane/diethyl ether 8:1).

^{1}H NMR (400 MHz, $CDCl_3$): 7.22 (ap d, *J* 8.8, 2H), 6.78 (ap d, *J* 8.8, 2H), 3.76 (s, 3H), 1.75 (ddd, *J* 13.8, 12.0, 5.3, 1H), 1.67 (ddd, *J* 13.8, 12.0, 5.4, 1H), 1.38 (s, 3H), 1.24–1.32 (2H, m), 1.19 (s, 12H), 1.06 (s, 6H), 1.04 (s, 6H), 0.55 (ddd, *J* 15.7, 12.0, 5.4, 1H), 0.45 (ddd, *J* 15.7, 12.0, 5.3, 1H).

^{13}C NMR (100 MHz, $CDCl_3$): 157.1, 141.4, 127.4, 113.0, 82.7, 82.6, 55.2, 40.1, 39.5, 26.0, 24.8, 24.7, 24.6, 24.4, *carbons attached to boron not observed*.

^{11}B NMR (128 MHz; $CDCl_3$): 33.1

IR (film): υ (cm^{-1}) 2977, 2930, 1610, 1513, 1356, 1318, 1249, 1145, 1037, 969, 848, 830,

HRMS (ESI) calcd. for $C_{24}H_{40}B_2NaO_5$ $[M+Na]^+$ 453.2963, found 453.2963.

$[\alpha]_D^{26} = +15$ (*c* 1, $CHCl_3$).

(*S*)-1-methoxy-4-(3-methylhexa-1,5-dien-3-yl)benzene (37)

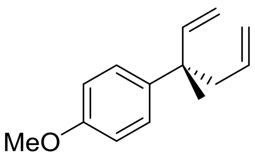

To stirred neat tetravinyltin (36 µl, 0.2 mmol) under an atmosphere of nitrogen at ambient temperature, was added *n*-BuLi (1.6 M in hexane, 0.5 ml, 0.8 mmol) dropwise and stirred for 30 min. Then the precipitate was washed with anhydrous hexane (~0.5 ml ×3). Anhydrous THF (0.5 mL) was added to dissolve the precipitate and the resulting homogenous solution was taken up into a syringe and was added dropwise to a stirred solution of boronic ester **15** (80 mg, 0.2 mmol) in anhydrous Et_2O (1 ml) under an atmosphere of nitrogen at −78 °C. The reaction mixture was stirred for 45 min at −78°C, then was warmed at ambient temperature and stirred for 20 min. The reaction mixture was cooled to −78 °C and a solution of iodine (203 mg, 0.8 mmol) in anhydrous THF (0.8 ml) was added dropwise. The reaction mixture was stirred at −78 °C for 30 min and then a suspension of NaOMe (86 mg, 1.6 mmol) in MeOH (2 ml) was added dropwise. The reaction mixture was stirred for 30 min at −78 °C and then allowed to reach ambient temperature and stirred for approximately 2 h. Sat. aq. $Na_2S_2O_3$ (5 ml) and water (2 ml) was added and the mixture stirred until the brown colour dissipated. The mixture was extracted with diethyl ether (3 × 10 ml), washed with aq. NaOH (5 mL, 1M solution), KF (5 mL) and brine. The organic phase was dried over $MgSO_4$ and the solvent was removed *in vacuo* to give **37** (77%, 32 mg) as a colourless oil.

$\boldsymbol{R_f}$ = 0.6 (Pentane/diethyl ether 20:1).

^{1}H NMR (400 MHz, $CDCl_3$): 7.24 (ap d, *J* 8.5, 2H), 6.85 (ap d, *J* 8.5, 2H), 6.02 (dd *J* 17.5, 10.7, 1H), 5.61 (ddt, *J* 17.3, 10.2, 7.4, 1H), 5.09 (dd, *J* 10.7, 1.3, 1H), 5.03 (dd, *J* 17.5, 1.3, 1H), 4.97–5.04 (m, 2H), 3.80 (s, 3H), 2.45–2.56 (m, 2H), 1.34 (s, 3H).

^{13}C NMR (100 MHz, $CDCl_3$): 157.6, 146.8, 139.0, 135.2, 127.7, 117.1, 113.4, 111.7, 55.2, 45.6, 43.4, 25.0.

IR (film): υ (cm^{-1}) 2972, 2901, 1625, 1597, 1512, 1410, 1249, 1183, 1066, 1038, 913, 828.

(*S*)-2-(4-methoxyphenyl)propane-1,2-diol (38)

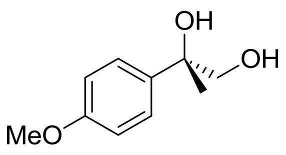

A premixed solution of 2M aq. NaOH/30% aq. H_2O_2 (2:1, 3 mL) was added dropwise to a solution of boronic ester **15** (80 mg, 0.2 mmol, 98:2 er) in THF (2 mL) at 0 °C. The reaction mixture was warmed to room temperature and stirred at this temperature for 4 h. The reaction mixture was diluted with H_2O (2 mL) and Et_2O (2 mL). The phases were separated and the aqueous phase washed with Et_2O (3 × 2 mL). The combined organic phases were washed with H_2O (5 mL), dried ($MgSO_4$) and concentrated under reduced pressure. Purification by flash chromatography gave diol **38** (98%, 36 mg, 98:2 er).

$\boldsymbol{R_f}$ = 0.29 (Ethyl acetate/Pentane 4:1).

^{1}H NMR (400 MHz, $CDCl_3$): 7.37 (ap d, *J* 8.8, 2H), 6.89 (ap d, *J* 8.8, 2H), 3.80 (s, 3H), 3.75 (d, *J* 11.1, 1H), 3.59 (d, *J* 11.1, 1H), 2.10 (bs, 2H), 1.51 (s, 3H).

^{13}C NMR (100 MHz, $CDCl_3$): 158.7, 137.0, 126.3, 113.7, 74.5, 71.1, 55.3, 26.0.

IR (film): υ (cm^{-1}) 3392, 2933, 1611, 1512, 1301, 1247, 1179, 1031, 831.

HRMS (ESI) calcd. for $C_{10}H_{14}NaO_3$ $[M+Na]^+$ 205.0835, found 205.0835.

$[\alpha]_D^{26}$ = +6 (*c* 1, $CHCl_3$).

Chiral SFC: Whelk-01, 125 bar, 42°C, 4 mL/min, 10% co-solvent (50% IPA/Hexane); t_R: 6.02 min (minor), 8.72 min (major), er 98:2.

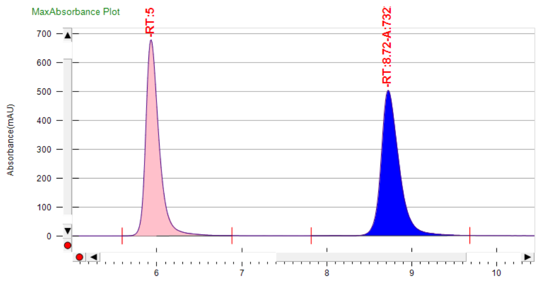

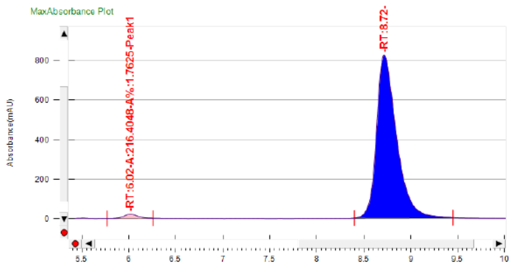

Peak No	% Area	Area	Ret. Time	Height	Cap. Factor
1	1.7625	216.4048	6.02 min	21.3935	6015.5833
2	98.2375	12062.114	8.72 min	825.1115	8715.5333

(*S*)-2-(2-(4-methoxyphenyl)propyl)-4,4,5,5-tetramethyl-1,3,2-dioxaborolane (39)

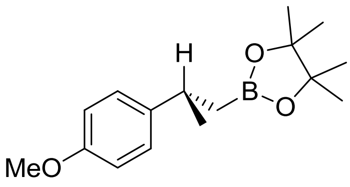

Boronic ester **15** (80 mg, 0.2 mmol, 1.0 equiv, 98:2 *er*) and tetra-*n*-butylammonium fluoride trihydrate (189 mg, 0.6 mmol, 3 equiv) were stirred in toluene at 90 °C for 2 h. Afterwards the mixture was filtered through a short silica column, concentrated in vacuo and the residue subjected to flash chromatography to afford primary boronic ester **39** (72%, 40 mg, 88:12 *er*).

***R*f** = 0.28 (Pentane/diethyl ether 8:1).

^{1}H NMR (400 MHz, $CDCl_3$): 7.15 (ap d, *J* 8.5, 2H), 6.81 (ap d, *J* 8.5, 2H), 3.78 (s, 3H), 3.03–2.94 (m, 1H), 1.24 (d, *J* 6.9, 3H), 1.16 (s, 12H), 1.14–1.10 (ap t, *J* 6.8, 2H).

^{13}C NMR (100 MHz, $CDCl_3$): 157.6, 141.5, 127.4, 113.5, 83.0, 55.3, 35.0, 25.1, 24.8, 24.7, *carbons attached to boron not observed.*

^{11}B NMR (128 MHz; $CDCl_3$): 33.0

IR (film): υ (cm^{-1}) 2977, 2956, 2926, 2835, 1735, 1612, 1513, 1367, 1322, 1246, 1144, 1038, 969, 829.

HRMS (ESI) calcd. for $C_{16}H_{25}BNaO_3$ $[M+Na]^+$ 299.1792, found 299.1780.

$[\alpha]_D^{26}$ = +18 (*c* 1, $CHCl_3$).

Chiral SFC: Whelk-01, 125 bar, 42°C, 2 mL/min, 10% co-solvent (10% IPA/Hexane); t_R: 19.98 min (minor), 20.82 min (major), *er* 88:12.

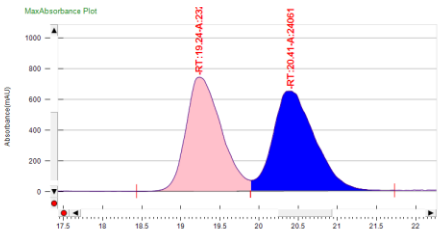

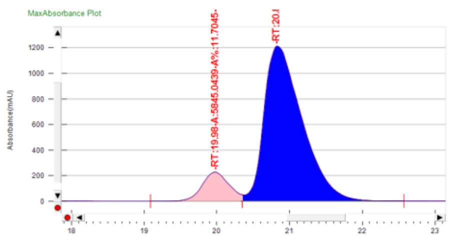

Peak No	% Area	Area	Ret. Time	Height	Cap. Factor
1	11.7045	5845.0439	19.98 min	227.5042	19975.6333
2	88.2955	44093.490 5	20.82 min	1211.6556	20822.4833

(*S*)-1-Methoxy-4-(1-phenylpropan-2-yl)benzene (40)

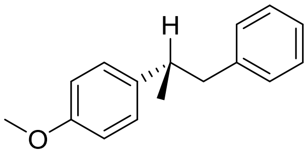

Following a reported procedure,[20] solid potassium hydroxide (33.6 mg, 0.6 mmol), $Pd(OAc)_2$/RuPhos (110 µL of a 1:1 solution in THF; 0.018 M) and bromobenzene (31.6 µL, 0.3 mmol) were added to a solution of boronic ester **15** (80 mg, 0.2 mmol) in 1.82 mL of THF in a vial with a magnetic stir bar. The vial was sealed and H_2O (sparged with N_2 for 30 min, 0.18 mL) was added through the teflon septum cap. The reaction mixture was then heated to 70 ºC and stirred for 12 h. Afterwards, the mixture was cooled to room temperature, diluted with diethyl ether (10 mL) and washed with H_2O and brine. The organic layer was dried over Na_2SO_4, filtered, and concentrated by rotary evaporation. Purification by flash chromatography of the crude material gave 1,2-diarylpropane **40** (75%, 34 mg). All spectroscopic data matched those previously reported.[21]

References

1. A. F. Burchat, J. M. Chong, N. Nielsen, *J. Organomet. Chem*. **1997**, *542*, 281–283.

2. N. A. Nikolic, P. Beak, *Org. Synth*. **1997**, *74,* 23

3. A. B. Pangborn, M. A. Giardello, R. H. Grubbs, R. K. Rosen, F. J. Timmers, *Organometallics* **1996**, *15*, 1518–1520.

4. P. Beak, L. G. Carter, *J. Org. Chem*. **1981**, *46*, 2363–2373.

5. R. Araya-Hermosilla, G.M.R. Lima, P. Raffa, G. Fortunato, A. Pucci, Mario E. Flores, I. Moreno-Villoslada, A.A. Broekhuis, F. Picchioni, *Eur. Polym. J.* **2016**, *81,* 186.

6. B. Eignerova, M. Dracinsky, M. Kotora, *Eur. J. Org. Chem*. **2008**, 4493.

7. C. Donner, *Org. Lett*. **2013**, *15*, 1258

8. R. Larouche-Gauthier, T. G. Elford, V. K. Aggarwal, *J. Am. Chem. Soc*., **2011**, *133*, 16794.

9. M. Burns, S. Essafi, J. R. Bame, S. P. Bull, M. P. Webster, S. Balieu, J. W. Dale, C. P. Butts, J. N. Harvey, V. K. Aggarwal, *Nature* **2014**, *513*, 183.

10. R. Larouche-Gauthier, C. J. Fletcher, I. Couto, V. K. Aggarwal, *Chem. Commun.*, **2011**, *47,* 12592.

11. C. Sandford, R. Rasappan, V. K. Aggarwal, *J. Am. Chem. Soc*. **2015**, *137,* 10100.

12. C. G. Watson, A. Balanta, T. G. Elford, S. Essafi, J. N. Harvey, V. K. Aggarwal, *J. Am. Chem. Soc*. **2014**, *136*, 17370

13. V. Bagutski, R. M. French, V. K. Aggarwal, *Angew. Chem. Int. Ed*. **2010**, *49*, 5142

14. J. L. Stymiest, V. Bagutski, R. M. French, V. K. Aggarwal, *Nature*, **2008**, *456*, 778
15. S. Nave, R. P. Sonawane, T. G. Elford, V. K. Aggarwal. *J. Am. Chem. Soc.*, **2010**, *132*, 17096.
16. R. Rasappan, V. K. Aggarwal, *Nature Chemistry* **2014**, *6*, 810
17. K. Toribatake, H. Nishiyama, *Angew. Chem. Int. Ed*. **2013**, *52*, 11011
18. C. G. Watson, A. Balanta, T. G. Elford, S. Essafi, J. N. Harvey, V. K. Aggarwal. *J. Am. Chem. Soc.*, **2014**, *136*, 17370
19. A. Fawcett, D. Nitsch, M. Ali, J. M. Bateman, E. L. Myers, V. K. Aggarwal. *Angew. Chem. Int. Ed.* **2016**, *55*, 14663.
20 S. N. Mlynarski, C. H. Schuster, J. P. Morken, Nature, **2014**, *505*, 386.
21 T. Bunlaksananusorn, K. Polborn, P. Knochel, *Angew. Chem. Int. Ed*. **2003**, *42*, 3941.

3-(2,5-dimethyl-1*H*-pyrrol-1-yl)propyl 2,4,6-triisopropylbenzoate (41)

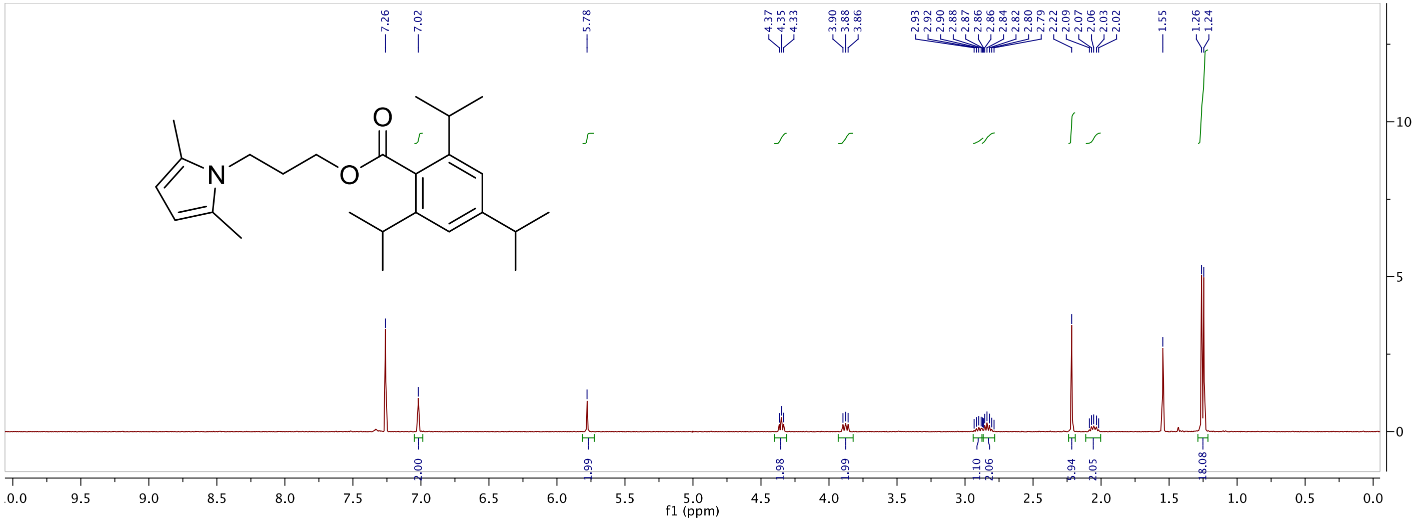

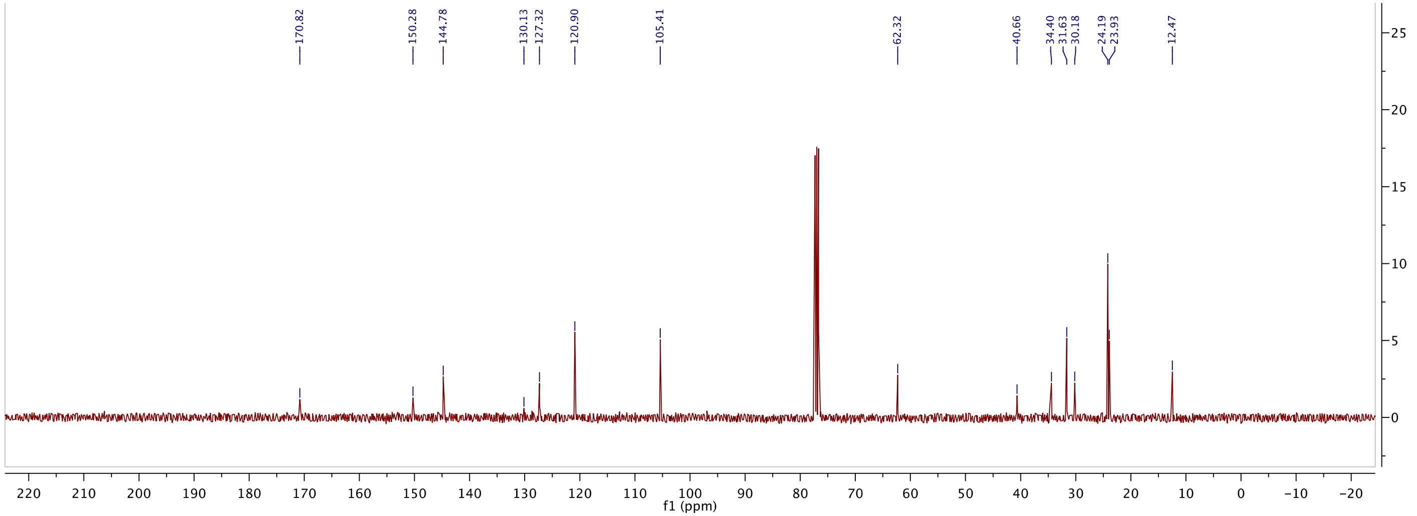

pent-4-en-1-yl 2,4,6-triisopropylbenzoate (42)

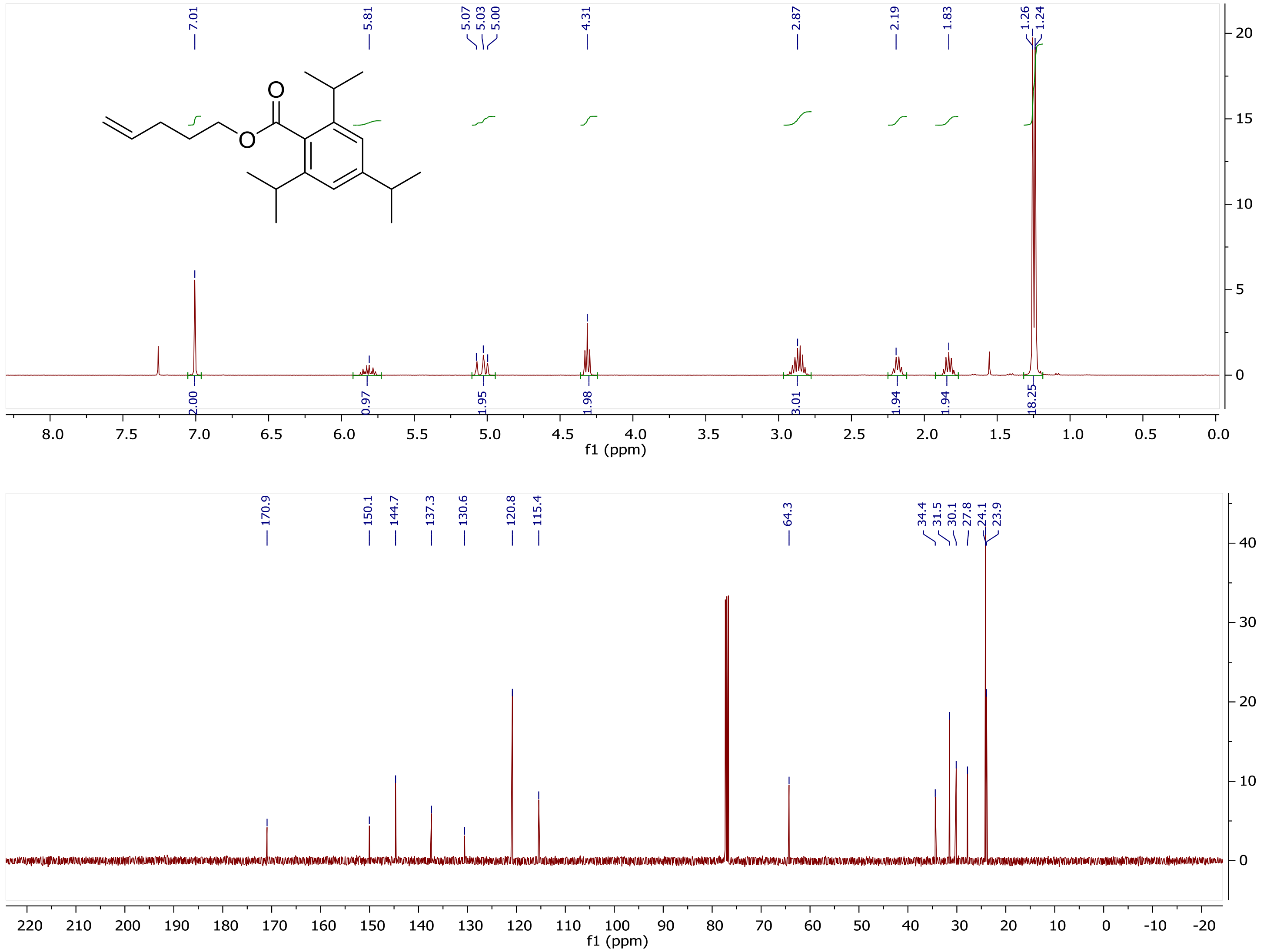

(4*R*)-4-((5*R*,8*R*,9*S*,10*S*,13*R*,14*S*,17*R*)-3-((tert-butyldimethylsilyl)oxy)-10,13-dimethylhexadecahydro-1H-cyclopenta[a]phenanthren-17-yl)pentyl 2,4,6-tritriisopropylbenzoate (43)

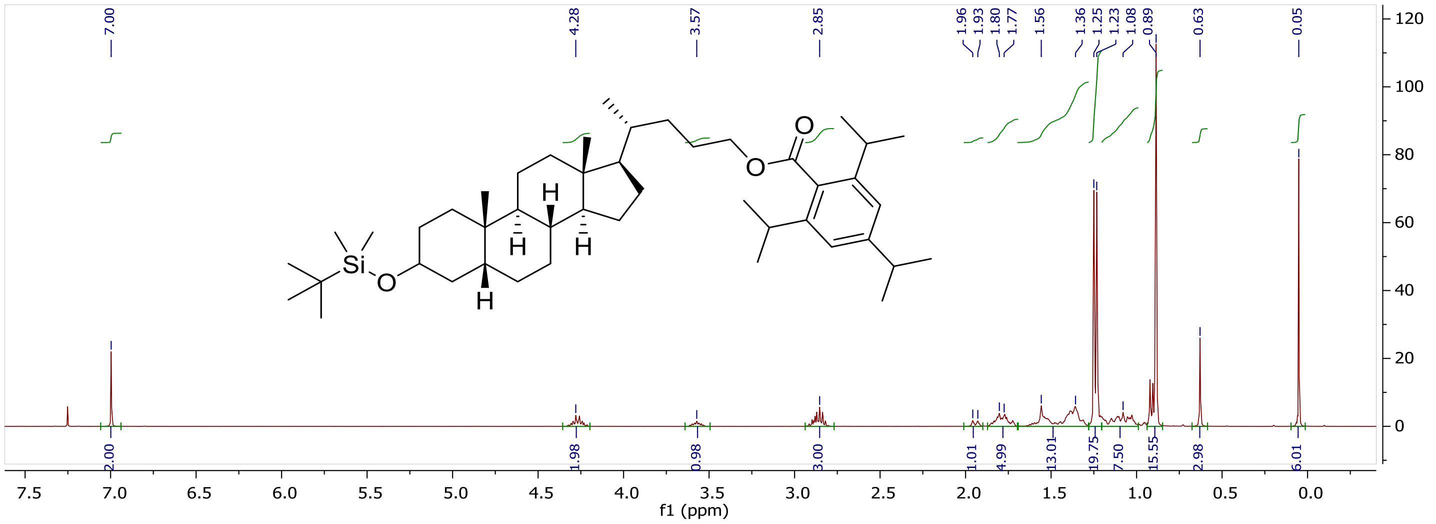

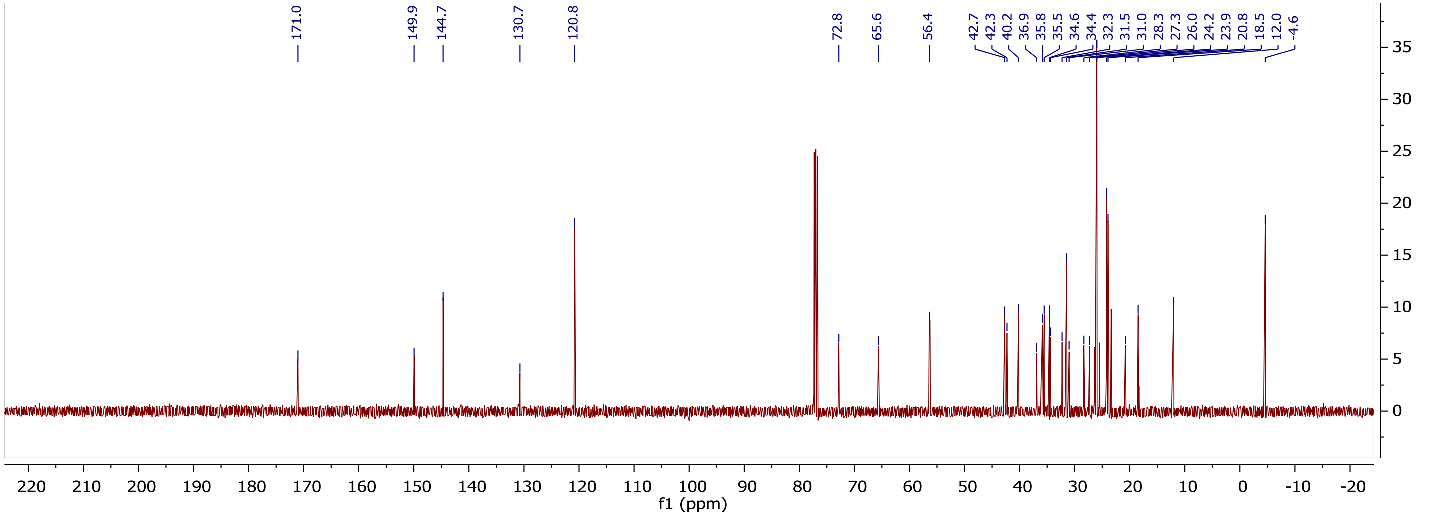

pent-4-yn-1-yl 2,4,6-triisopropylbenzoate (44)

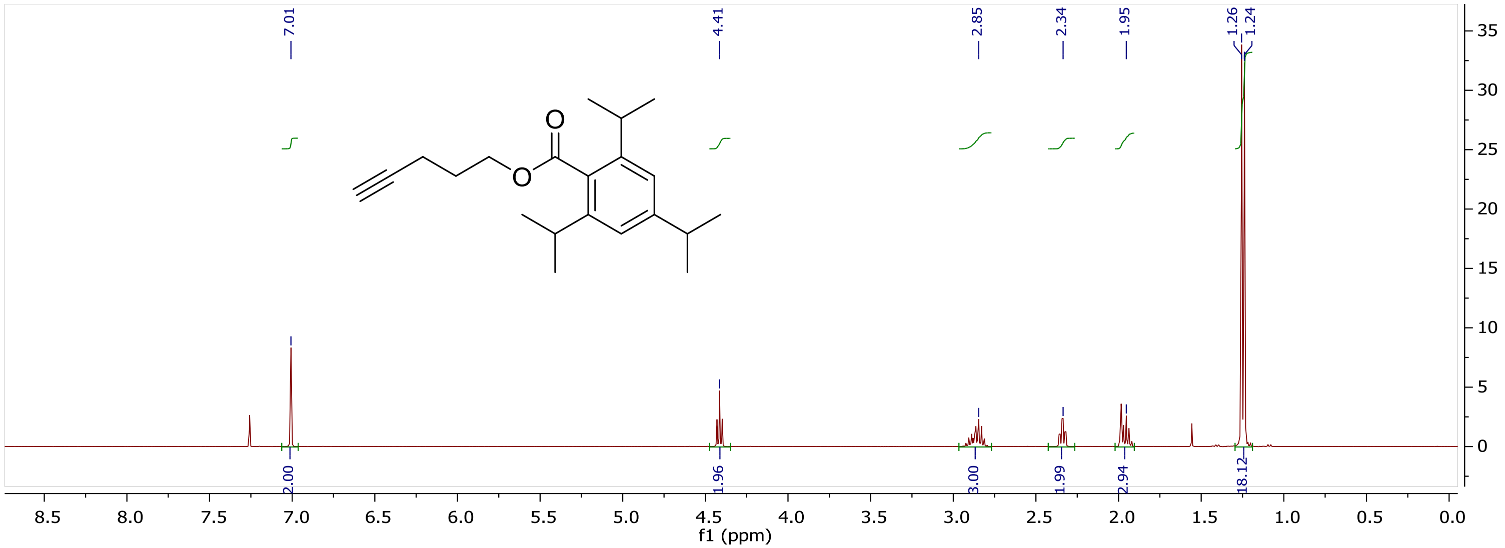

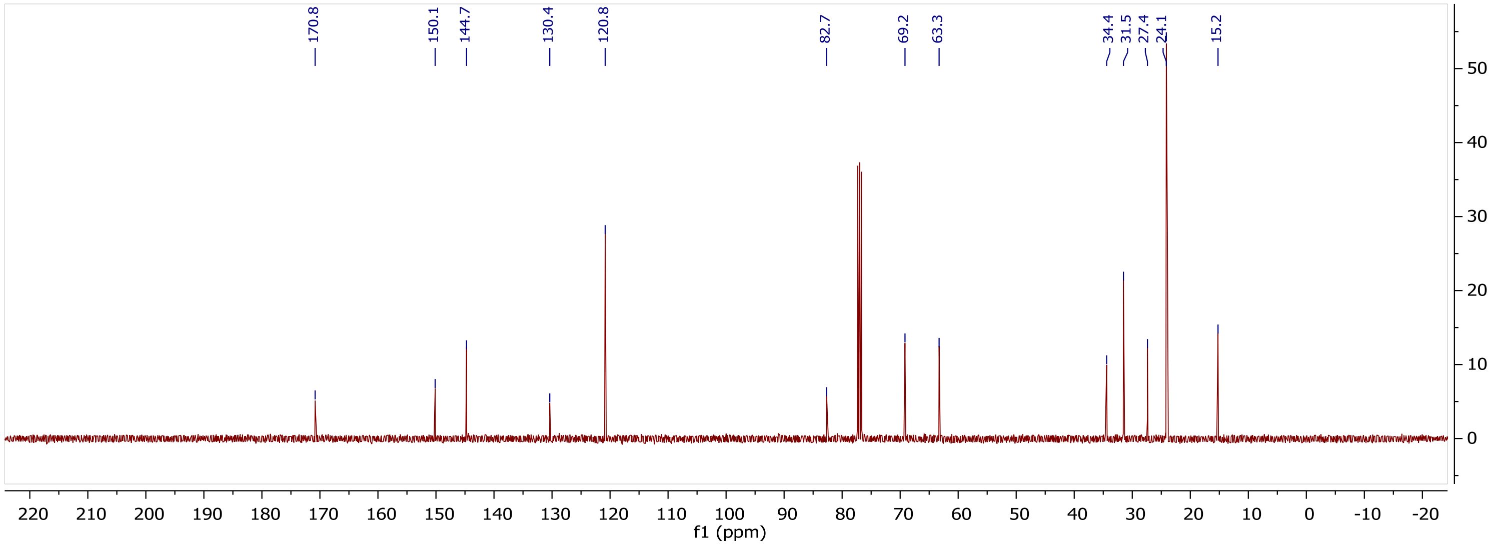

3-((*tert*-butyldimethylsilyl)oxy)propyl 2,4,6 triisopropylbenzoate (45)

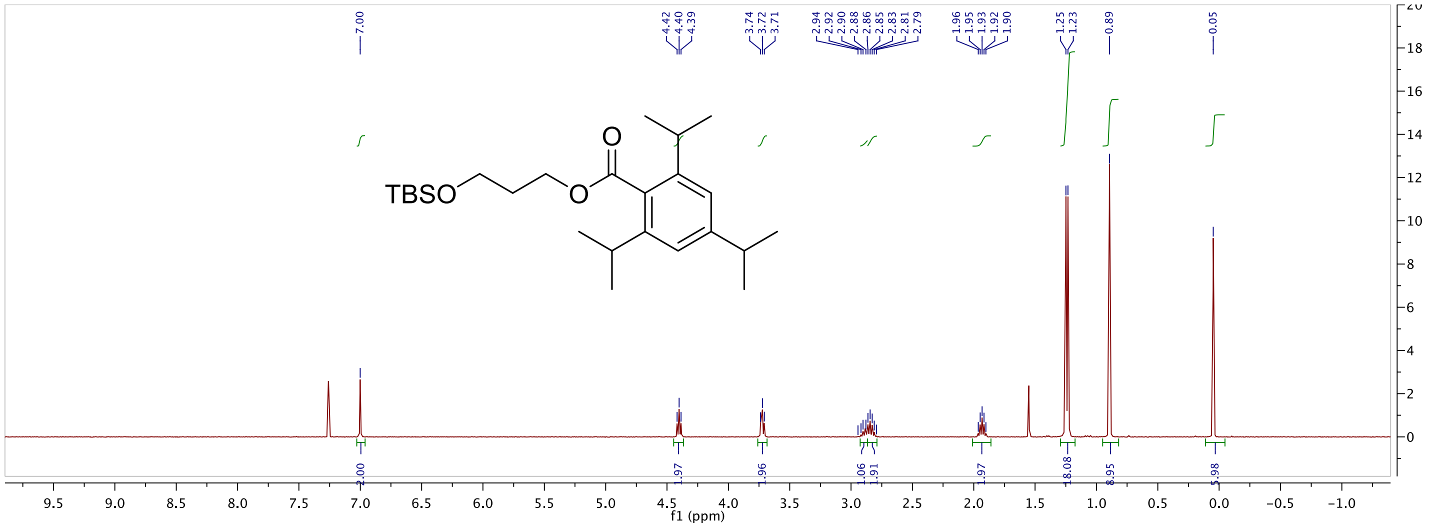

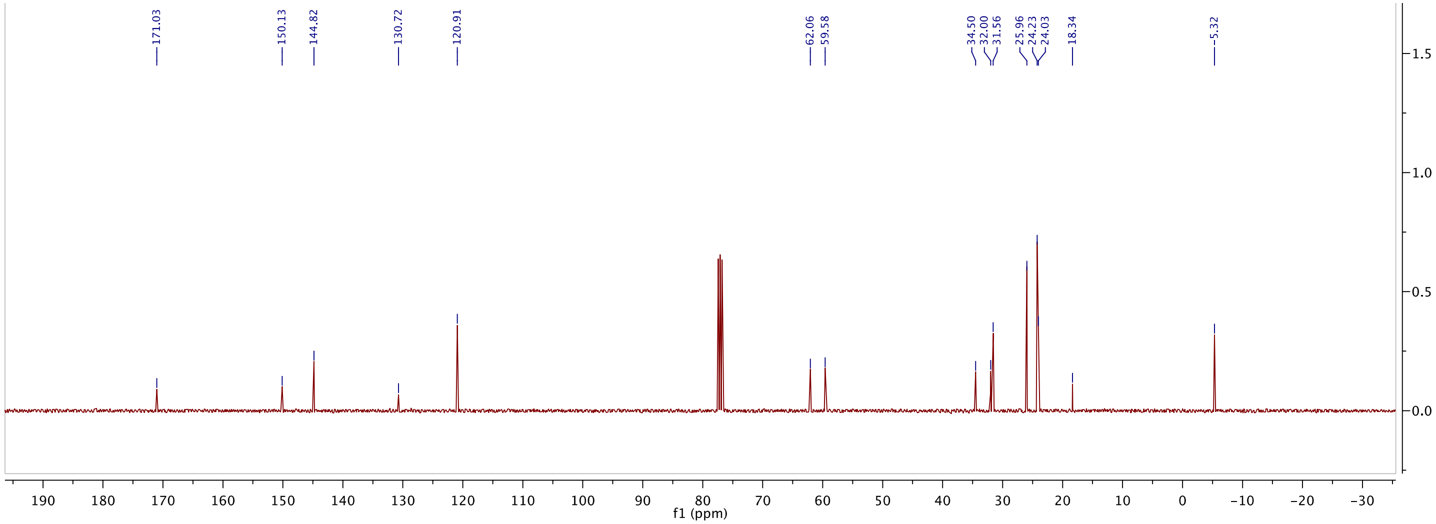

(*R*)-3-(4-methoxyphenyl)-1-(tributylstannyl)propyl 2,4,6-triisopropylbenzoate (46)

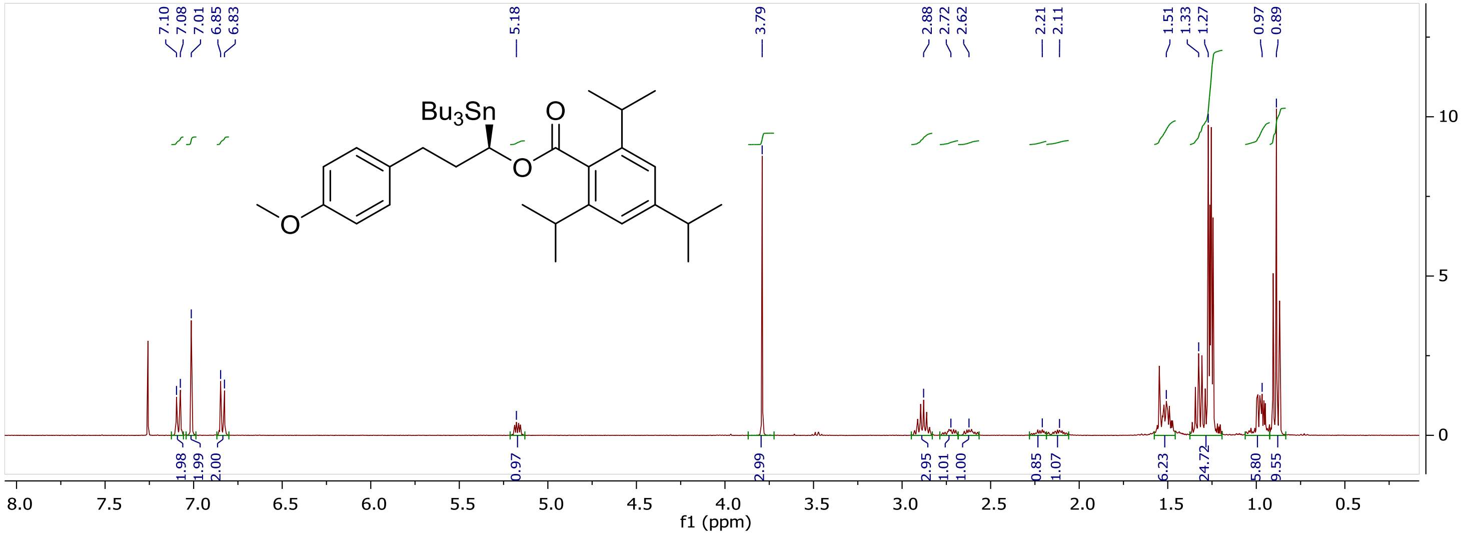

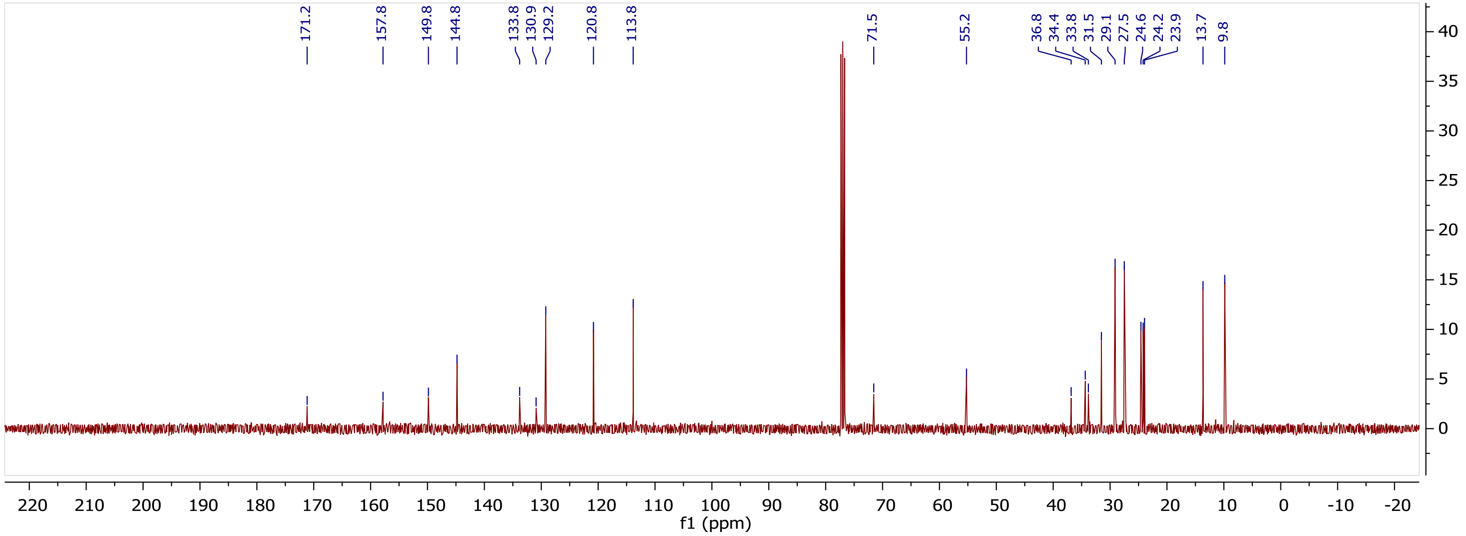

(*S*)-1-(Trimethylstannyl)ethyl 2,4,6-triisopropylbenzoate (47)

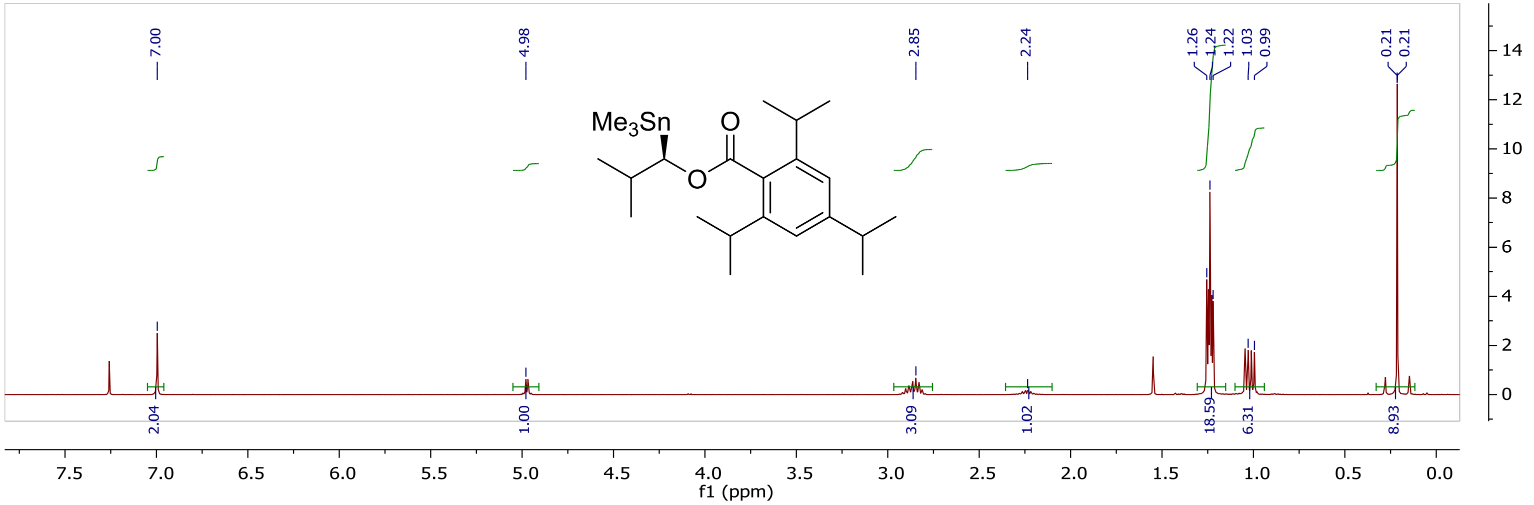

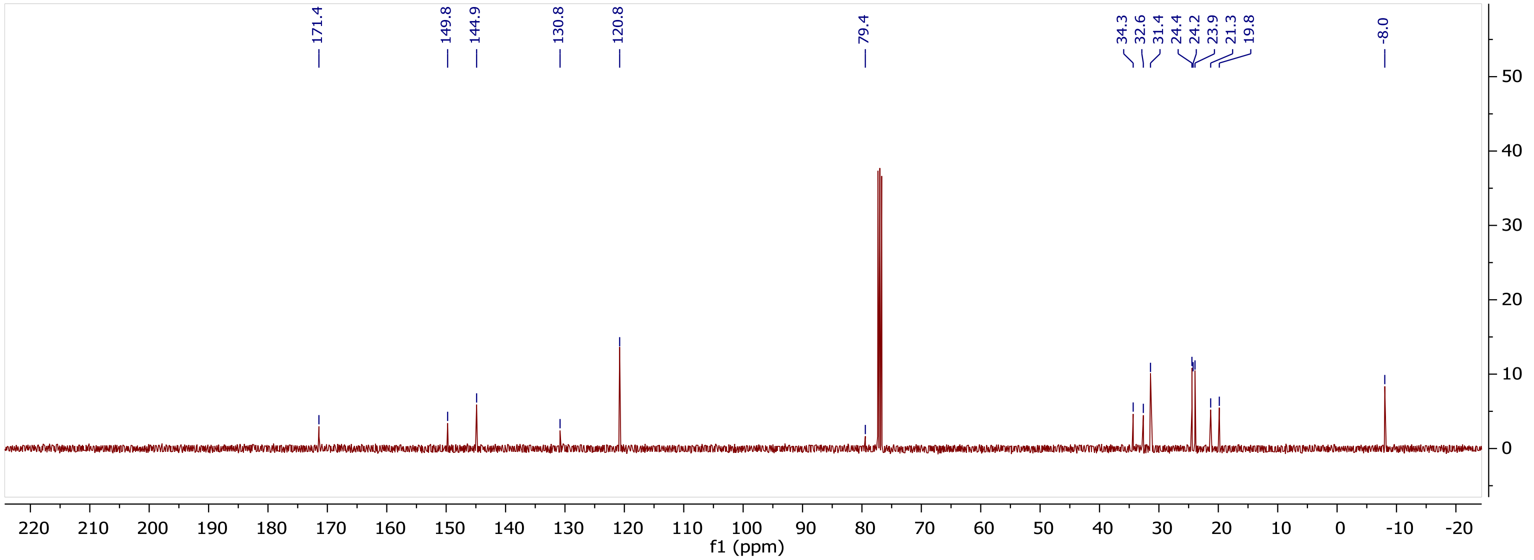

(*R*)-3-phenyl-1-(trimethylstannyl)propyl 2,4,6-triisopropylbenzoate (48)

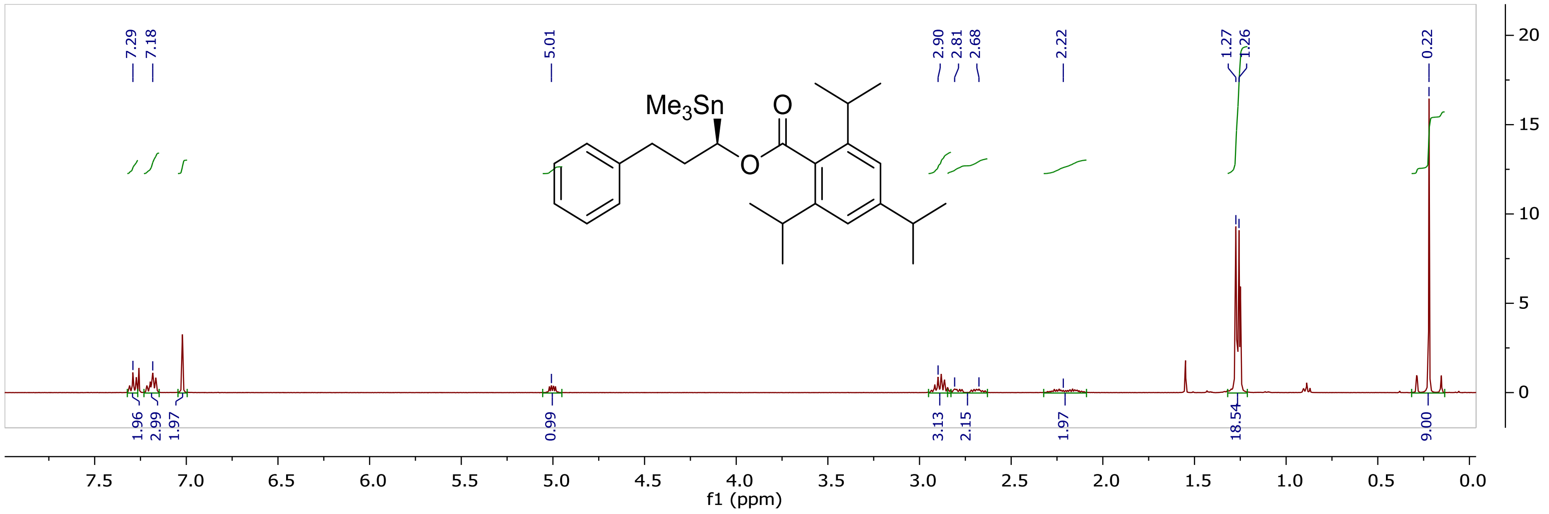

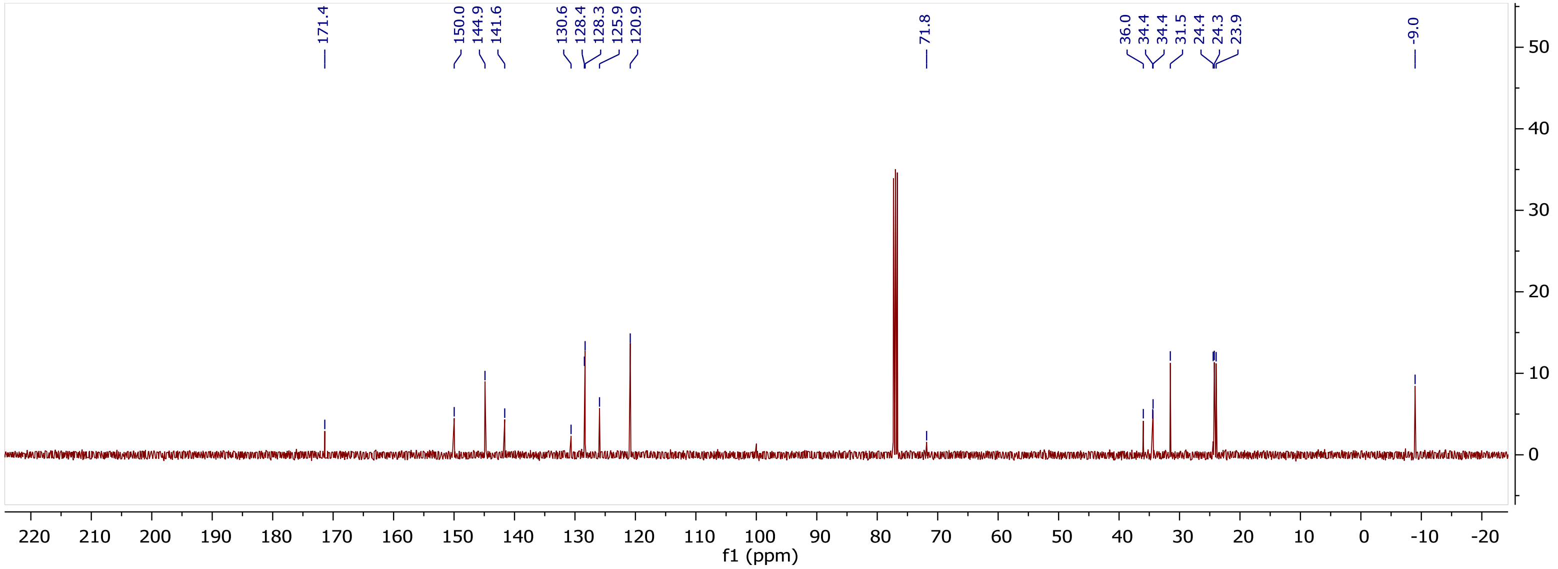

3-((*tert*-butyldimethylsilyl)oxy)-1-(trimethylstannyl)propyl 2,4,6 triisopropylbenzoate (49)

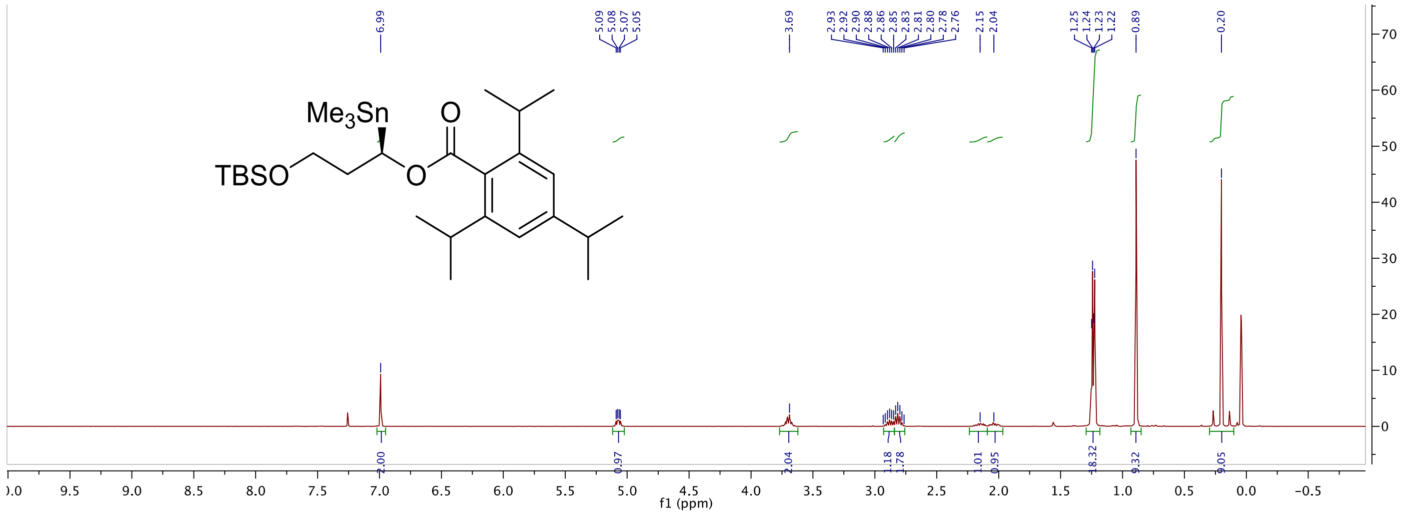

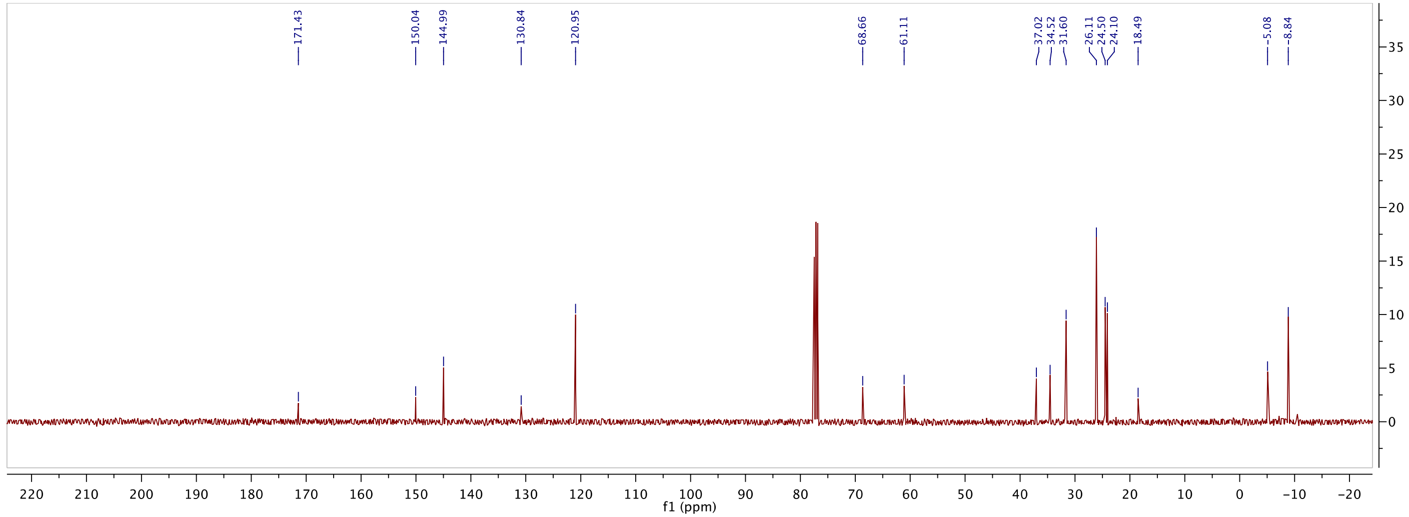

(*R*)-1-(trimethylstannyl)pent-4-en-1-yl 2,4,6-triisopropylbenzoate (50)

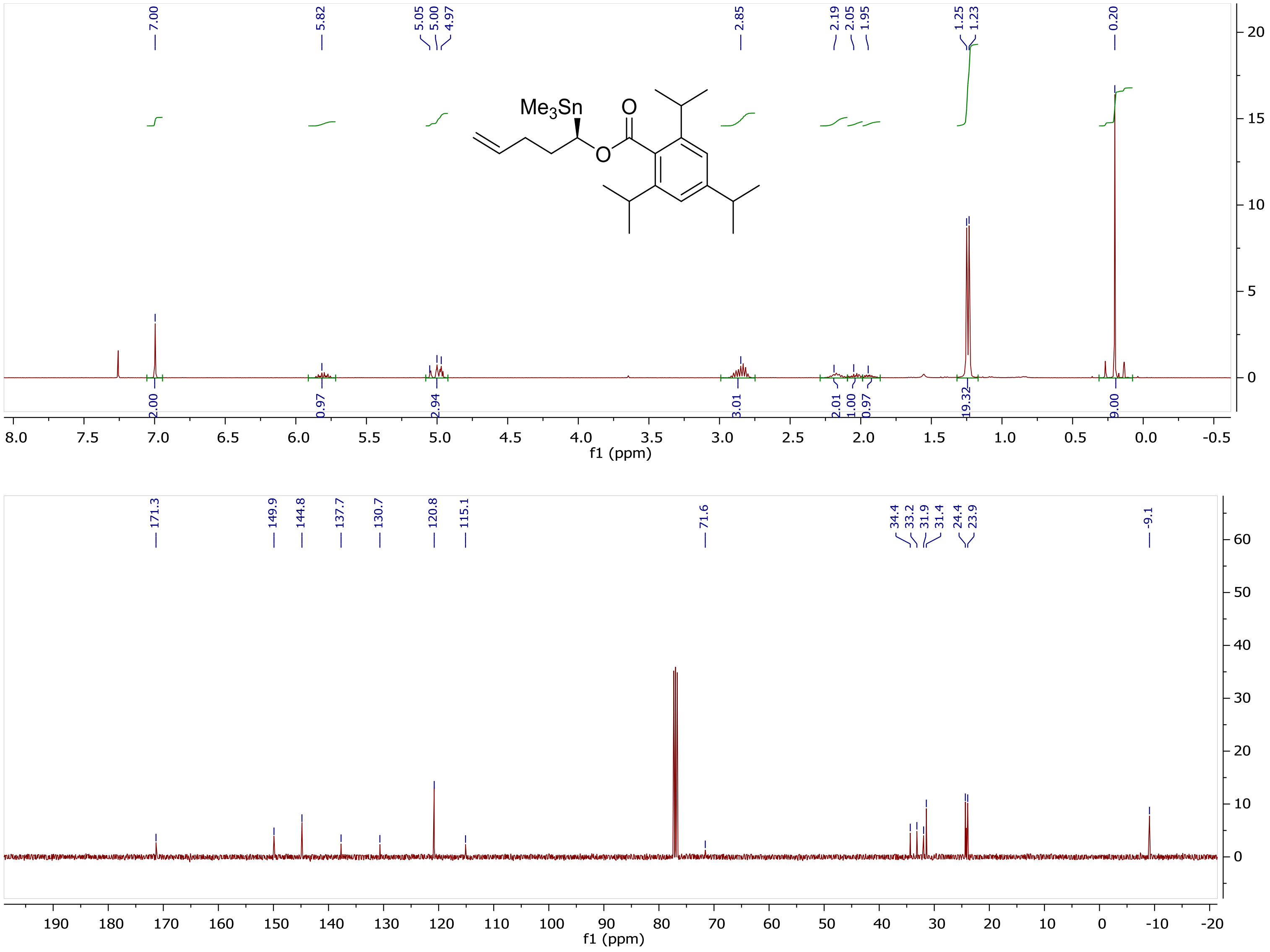

(*R*)-2-methyl-1-(trimethylstannyl)propyl 2,4,6-triisopropylbenzoate (51)

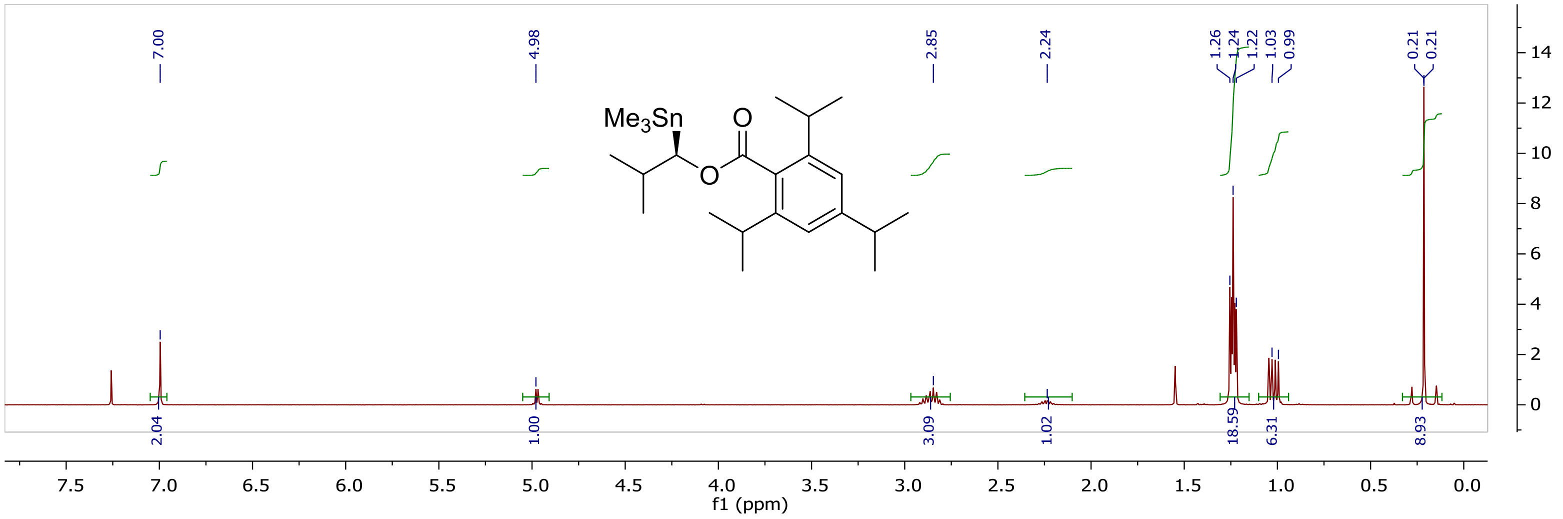

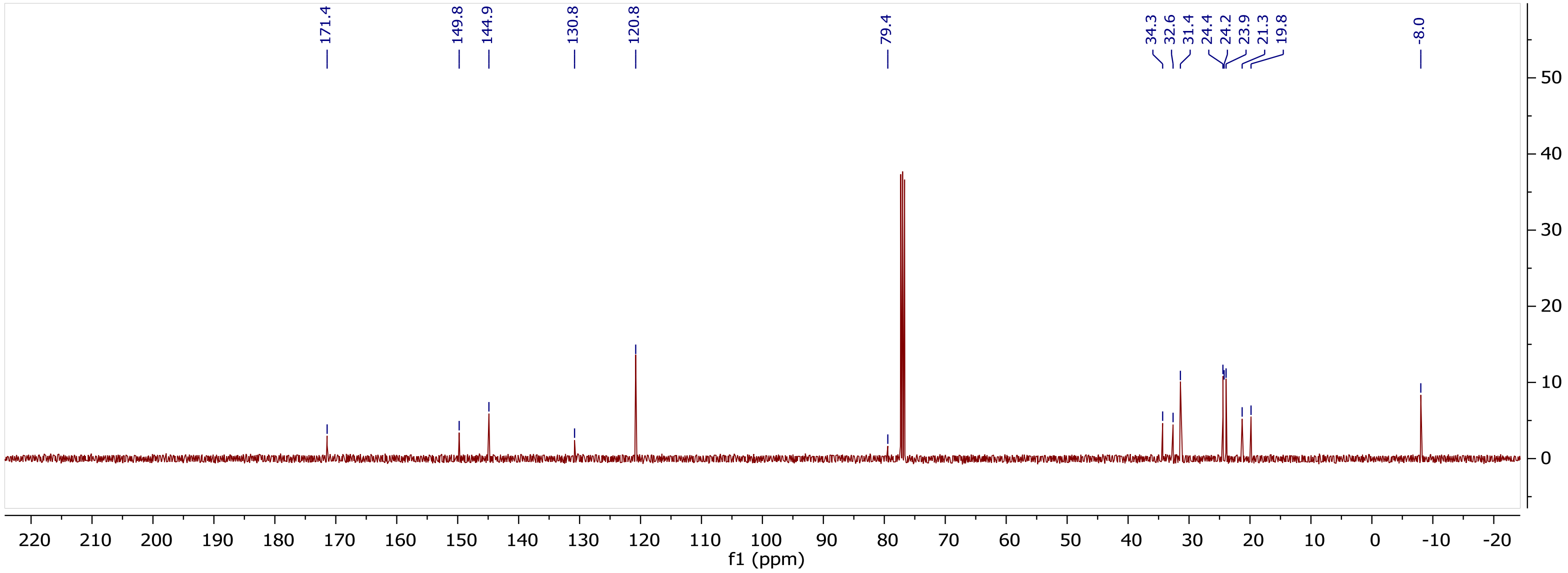

(*R*)-2,2'-(4-(4-methoxyphenyl)butane-1,2-diyl)bis(4,4,5,5-tetramethyl-1,3,2-dioxaborolane) (3)

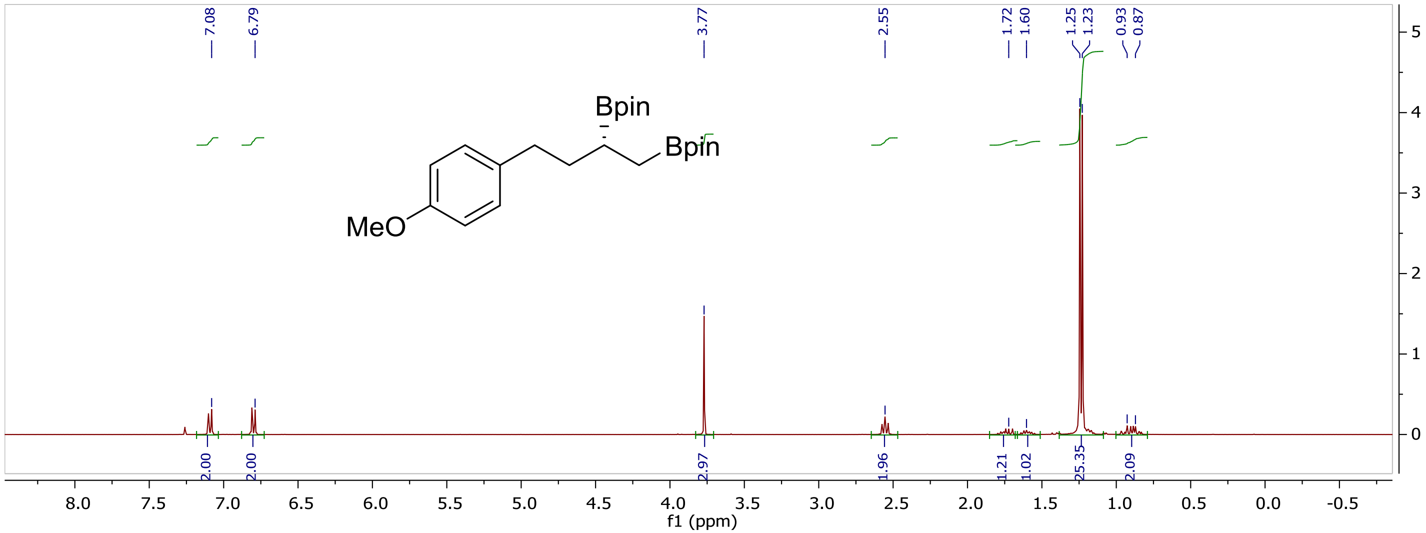

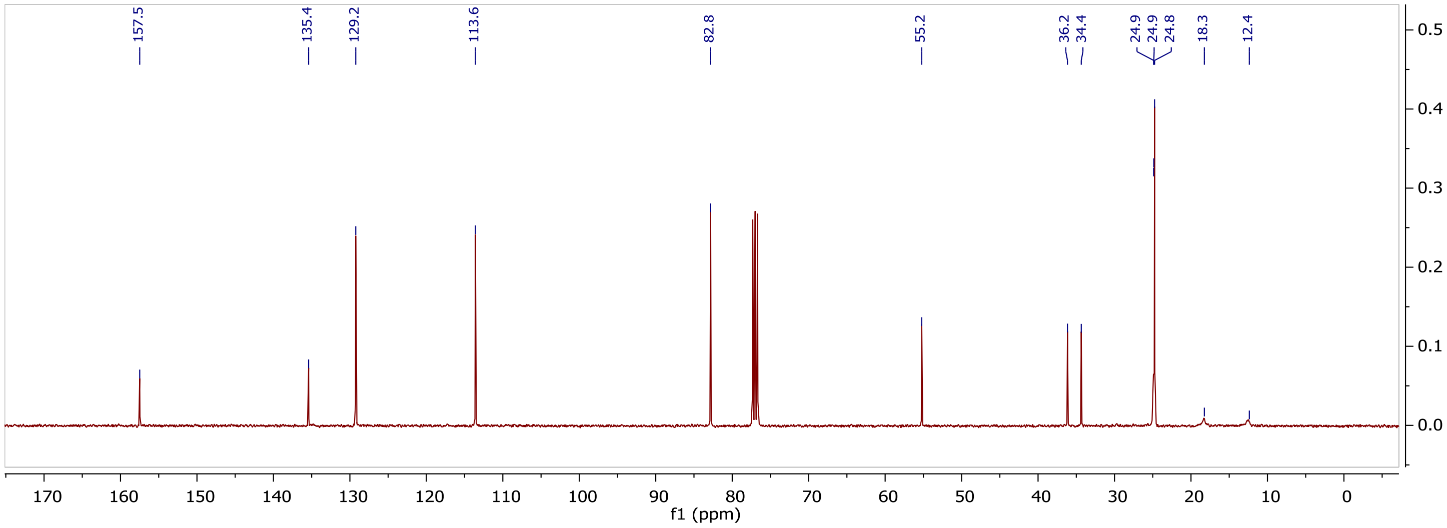

(*R*)-1-(3,4-bis(4,4,5,5-tetramethyl-1,3,2-dioxaborolan-2-yl)butyl)-2,5-dimethyl-1*H*-pyrrole (5)

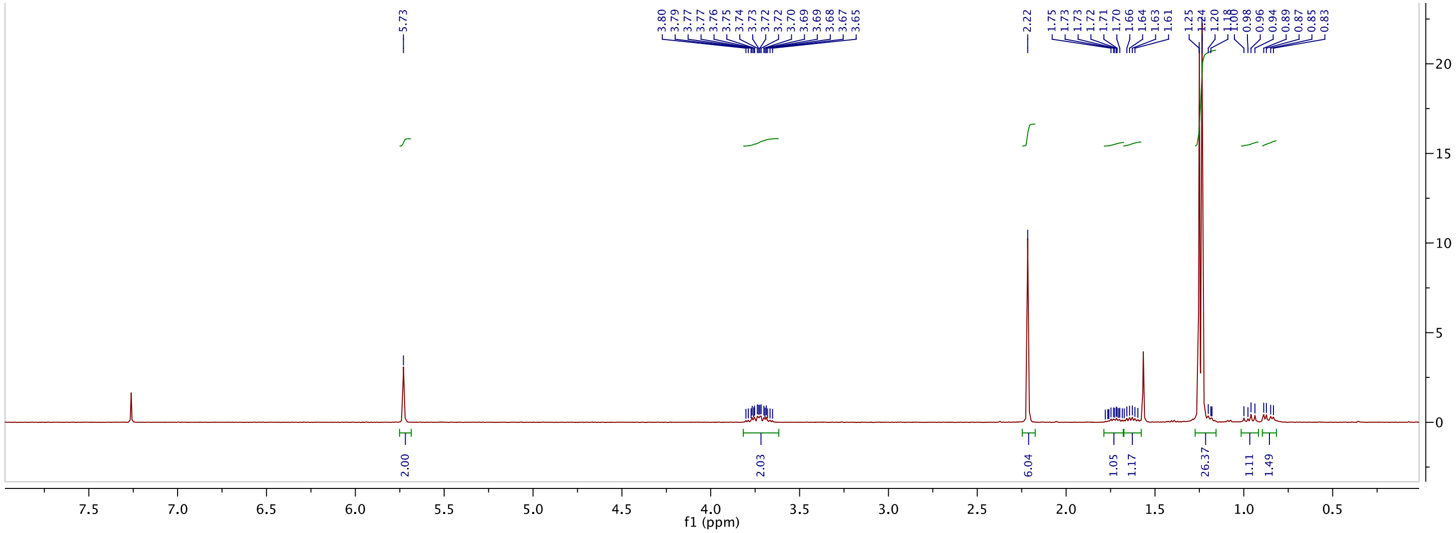

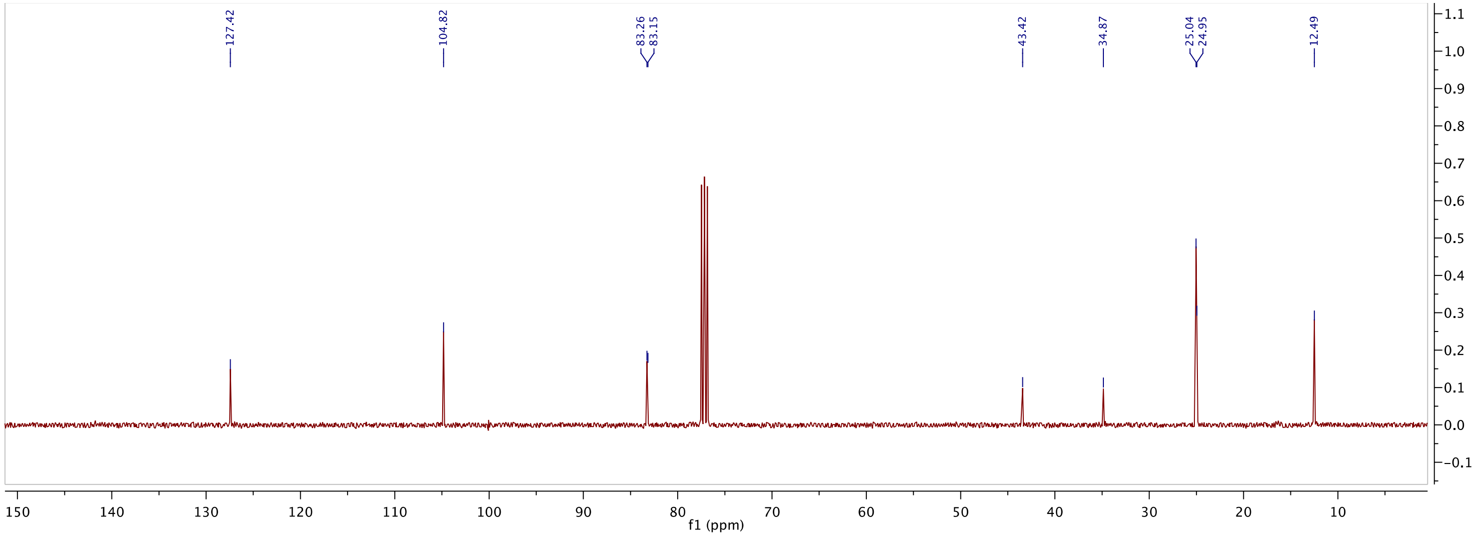

(*R*)-2,2'-(hex-5-ene-1,2-diyl)bis(4,4,5,5-tetramethyl-1,3,2-dioxaborolane) (6)

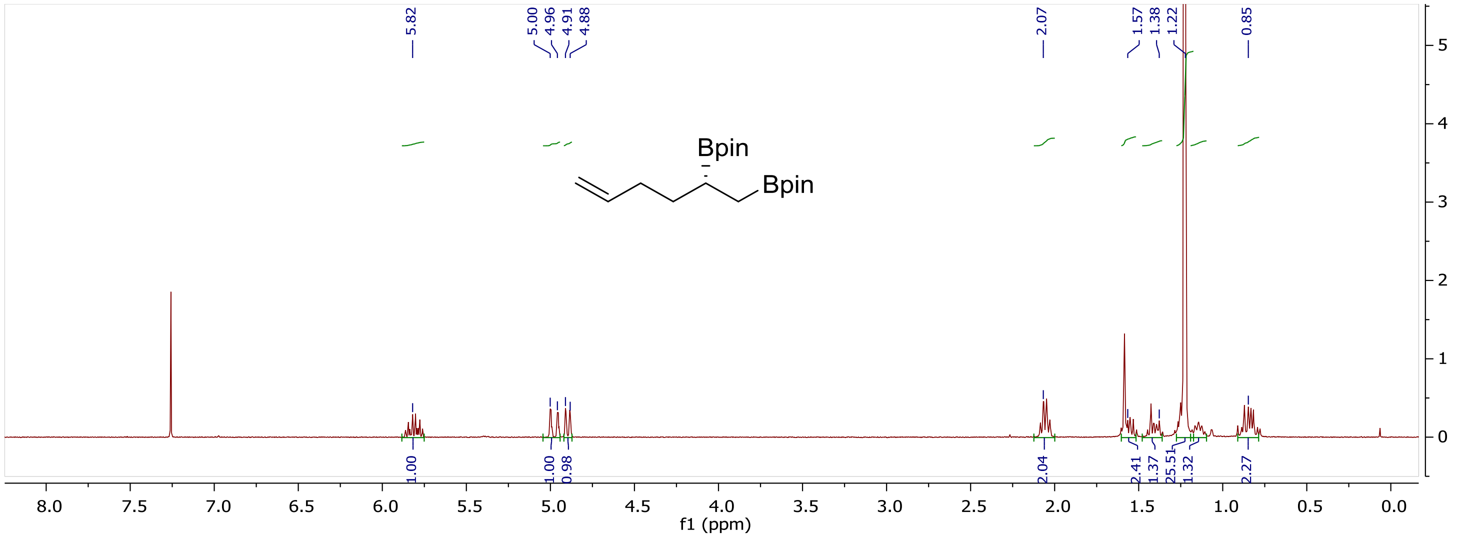

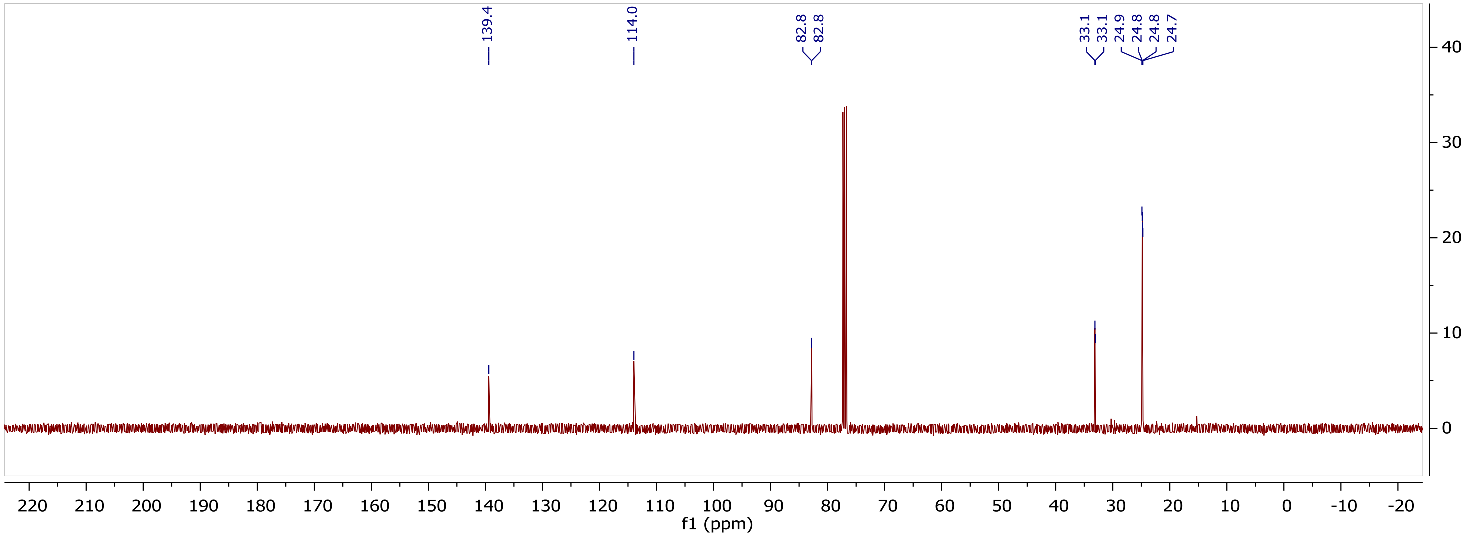

(((3*R*,5*R*,8*R*,9*S*,10*S*,13*R*,14*S*,17*R*)-17-((2*R*,5*S*)-5,6-bis(4,4,5,5-tetramethyl-1,3,2-dioxaborolan-2-yl)hexan-2-yl)-10,13-dimethylhexadecahydro-1H-cyclopenta[a]phenanthren-3-yl)oxy)(*tert*-butyl)dimethylsilane (7)

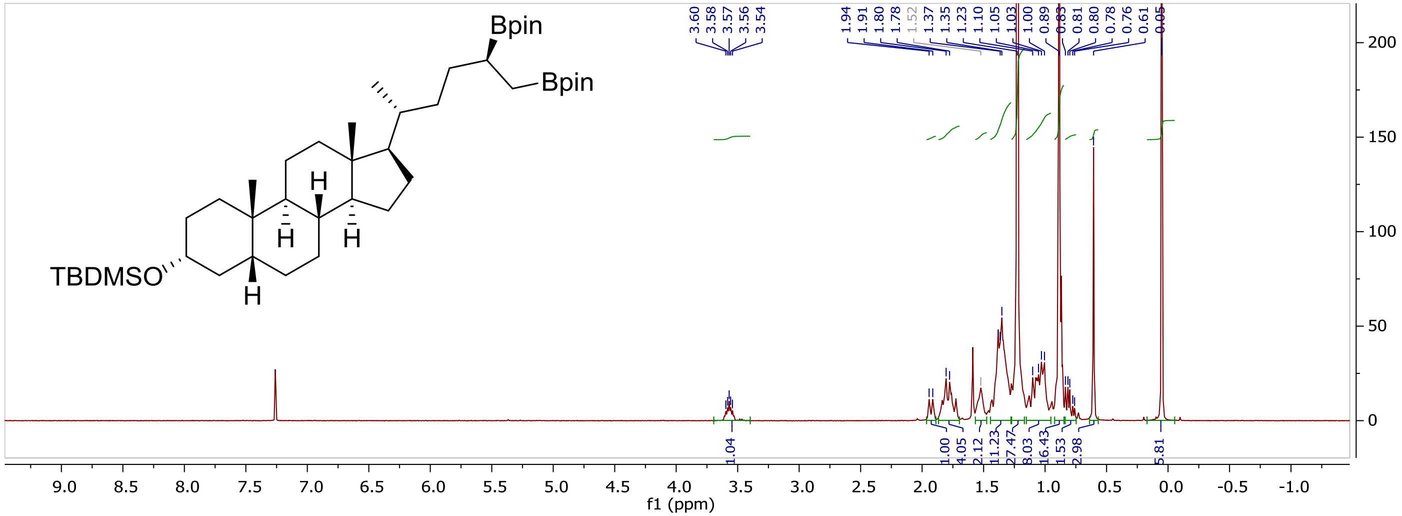

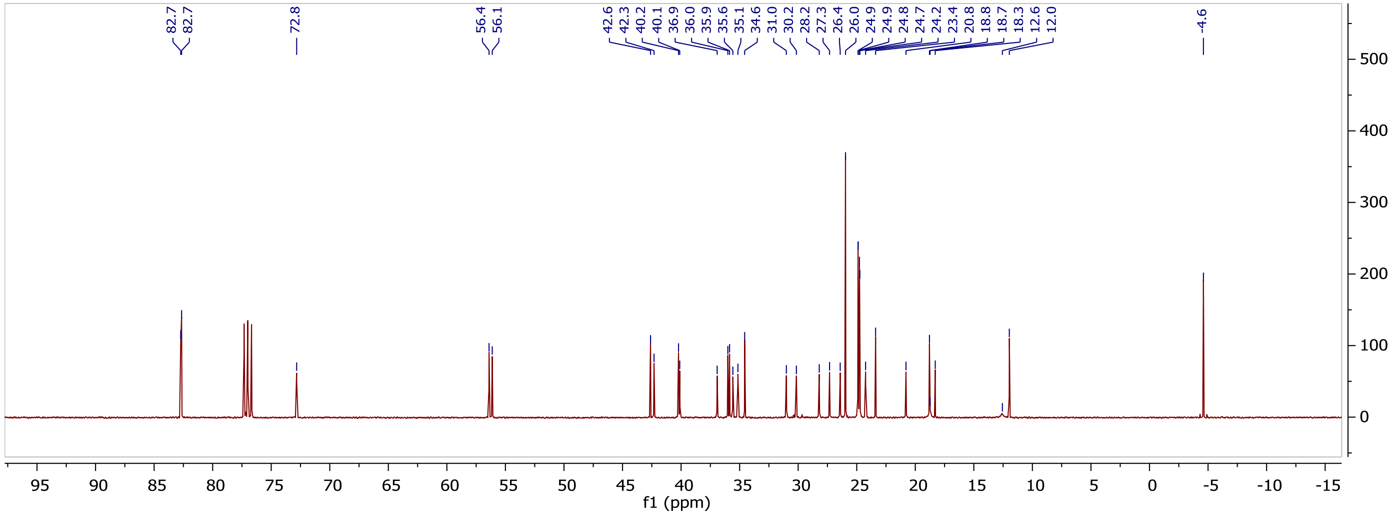

2,2'-((2*S*,3*R*,5*S*)-6-(methoxymethoxy)-3,5-dimethylhexane-1,2-diyl)bis(4,4,5,5-tetramethyl-1,3,2-dioxaborolane) (8)

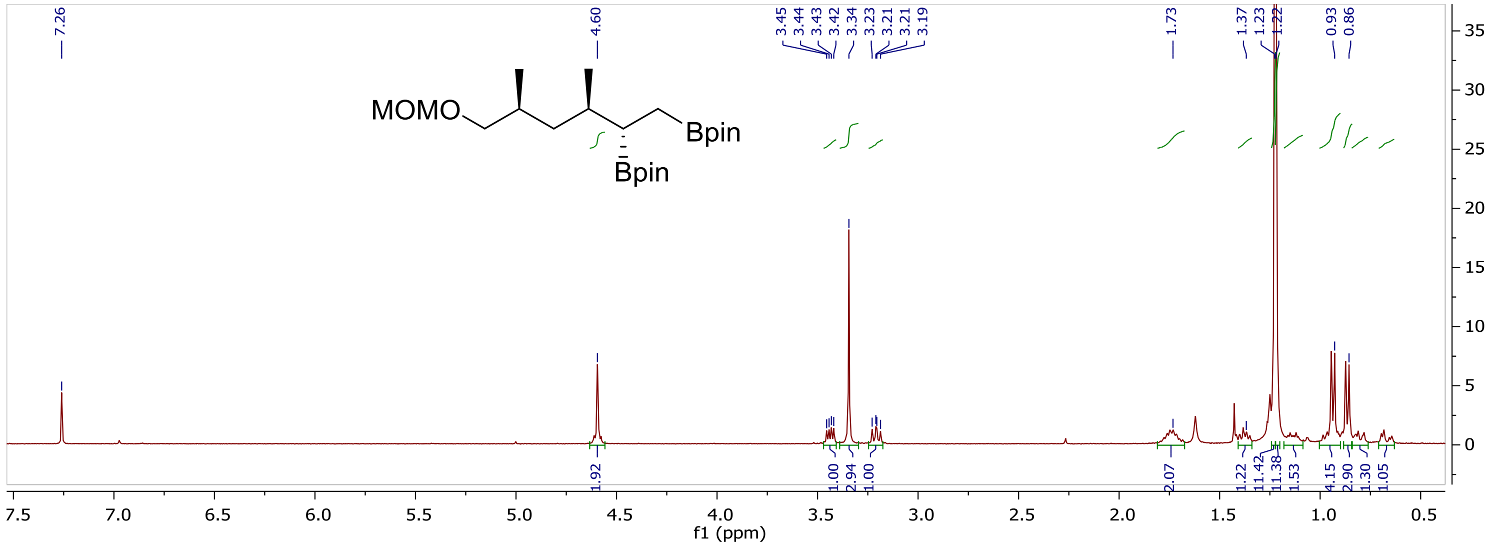

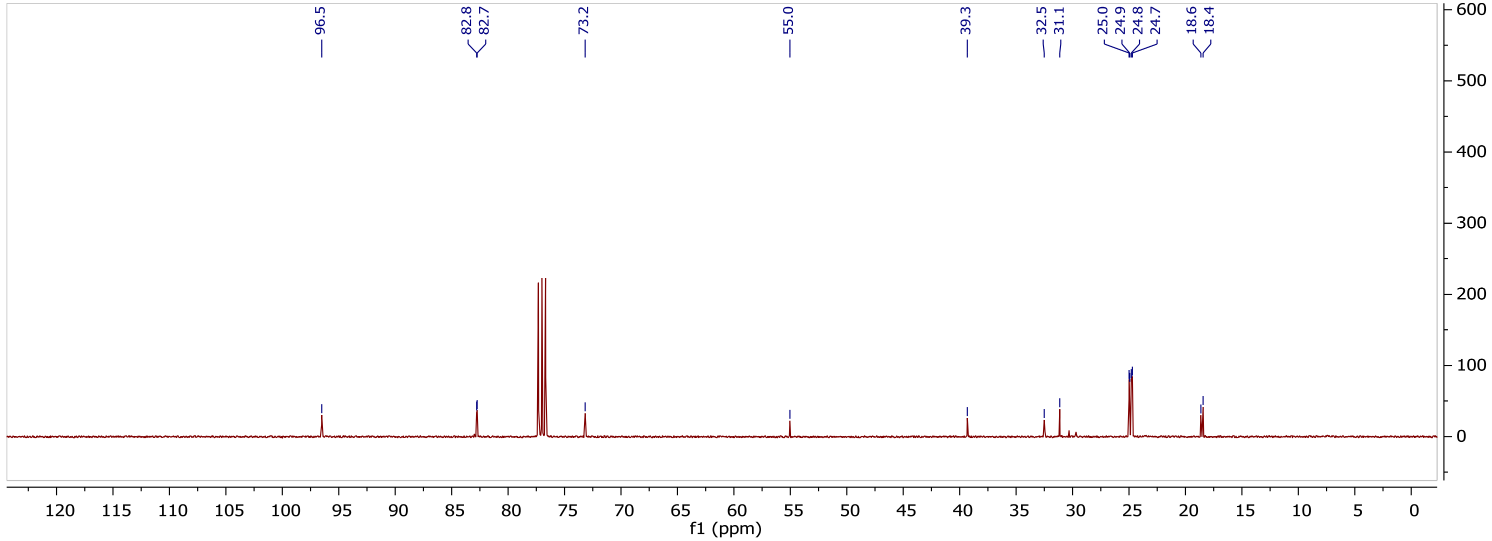

2,2'-((2*R*,3*R*,5*S*)-6-(methoxymethoxy)-3,5-dimethylhexane-1,2-diyl)bis(4,4,5,5-tetramethyl-1,3,2-dioxaborolane) (9)

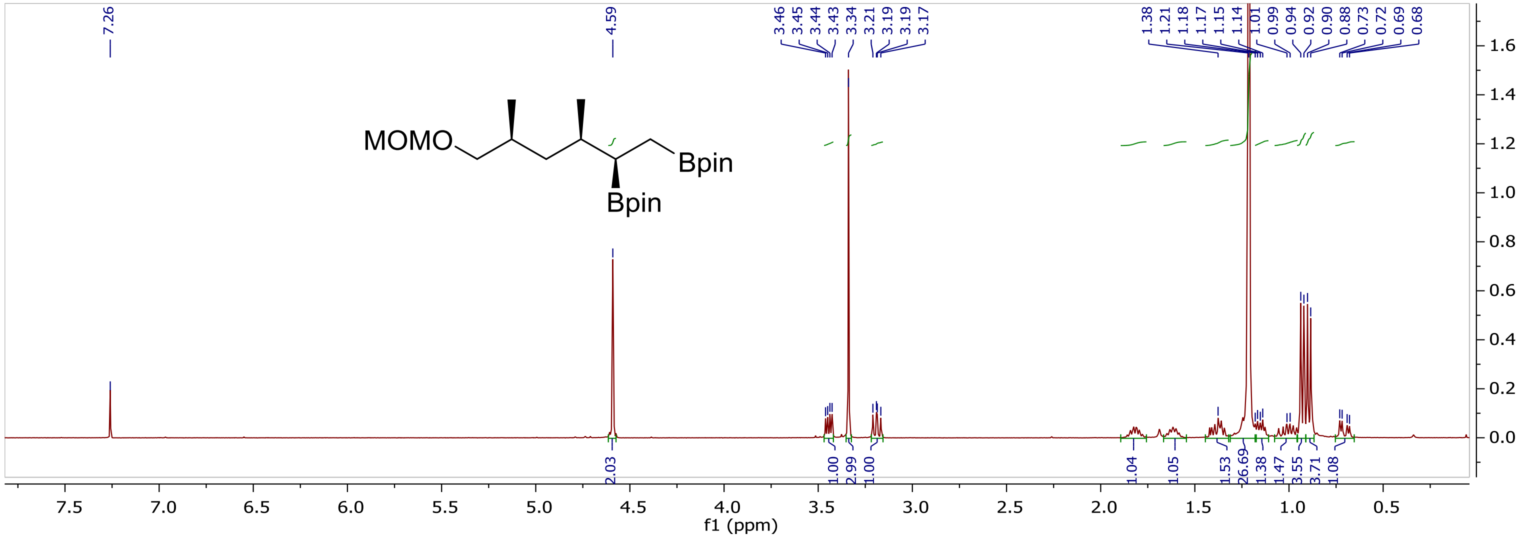

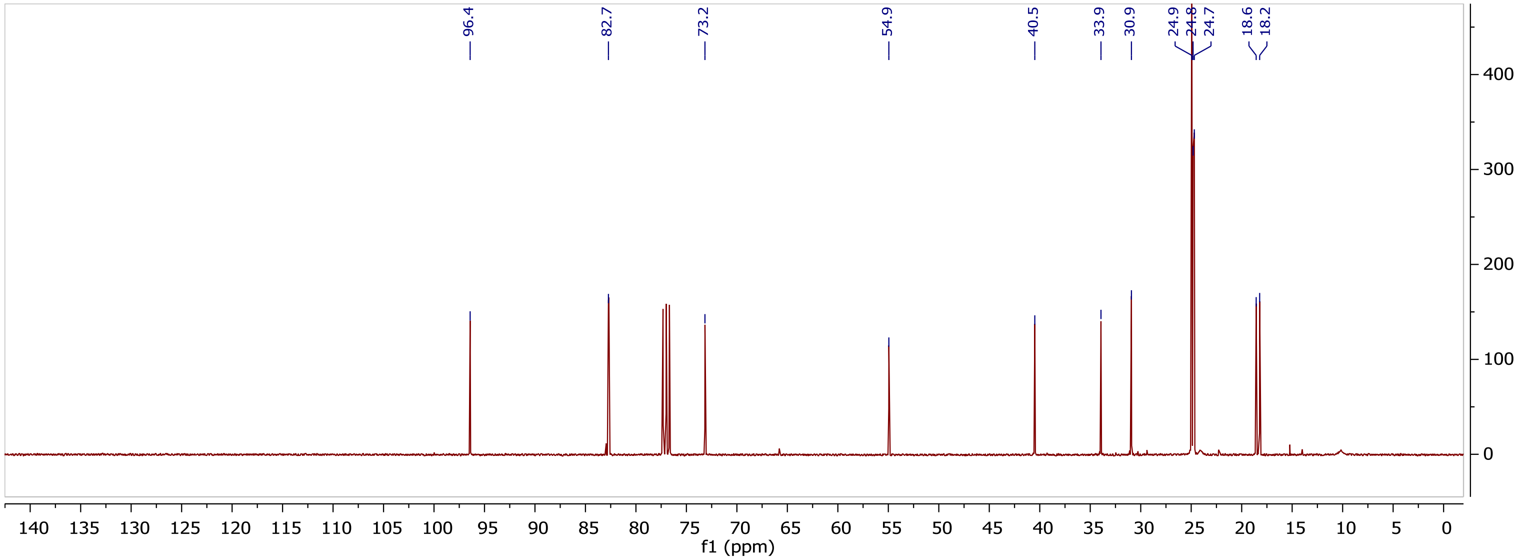

(*R*)-2,2'-(hex-5-yne-1,2-diyl)bis(4,4,5,5-tetramethyl-1,3,2-dioxaborolane) (10)

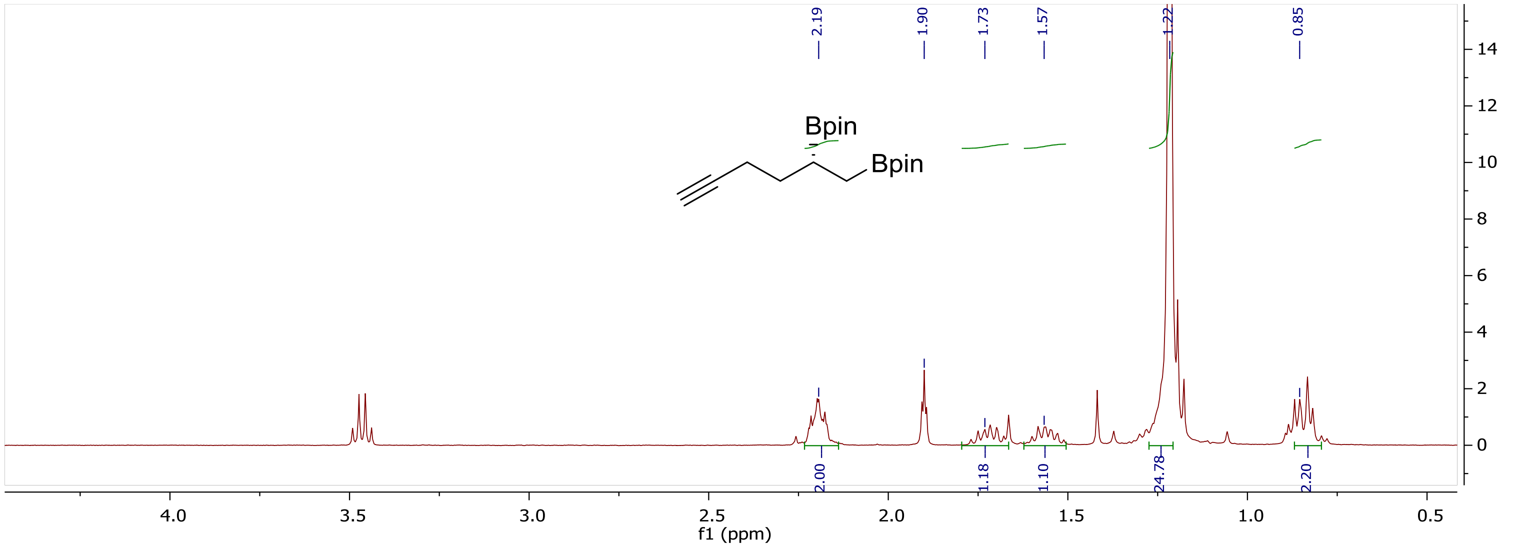

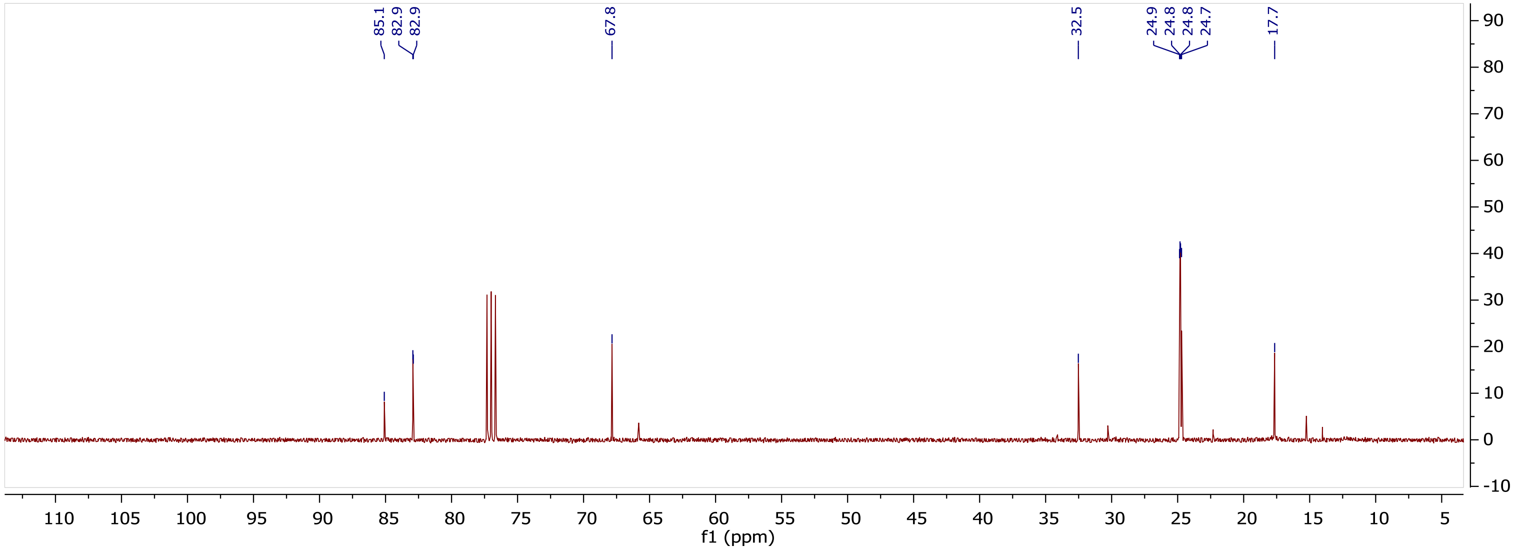

(*S*)-2,2'-(2-((1,1'-biphenyl)-4-yl)propane-1,2-diyl)bis(4,4,5,5-tetramethyl-1,3,2 dioxaborolane) (12)

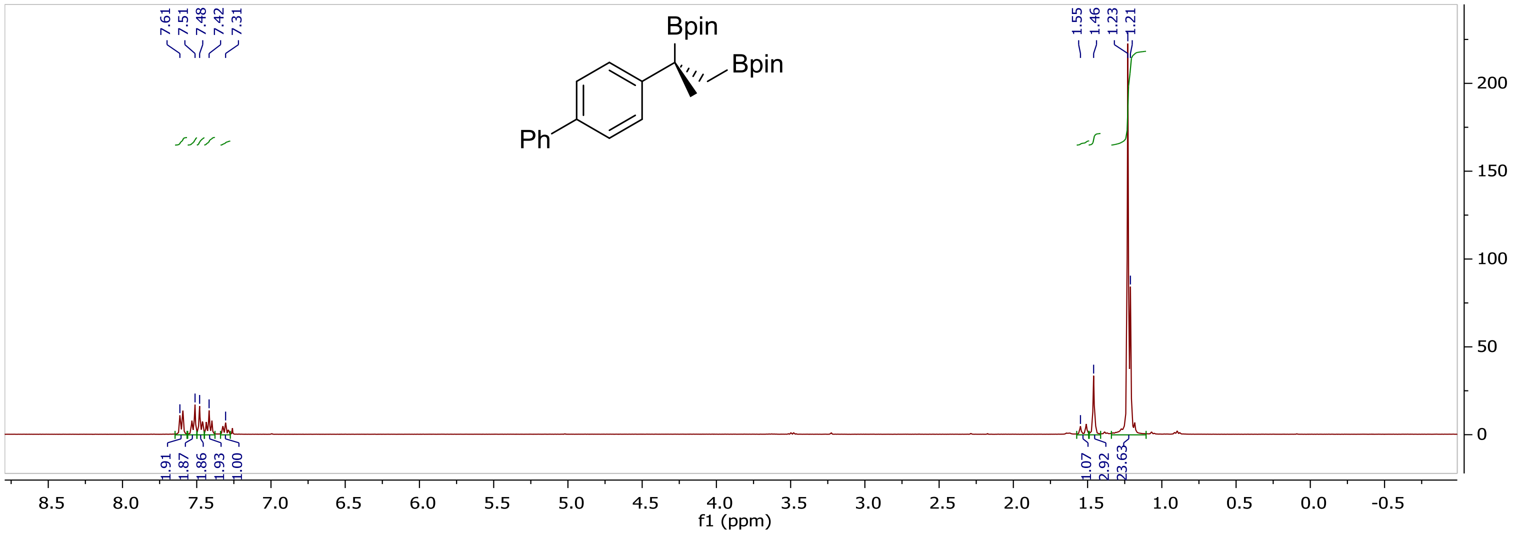

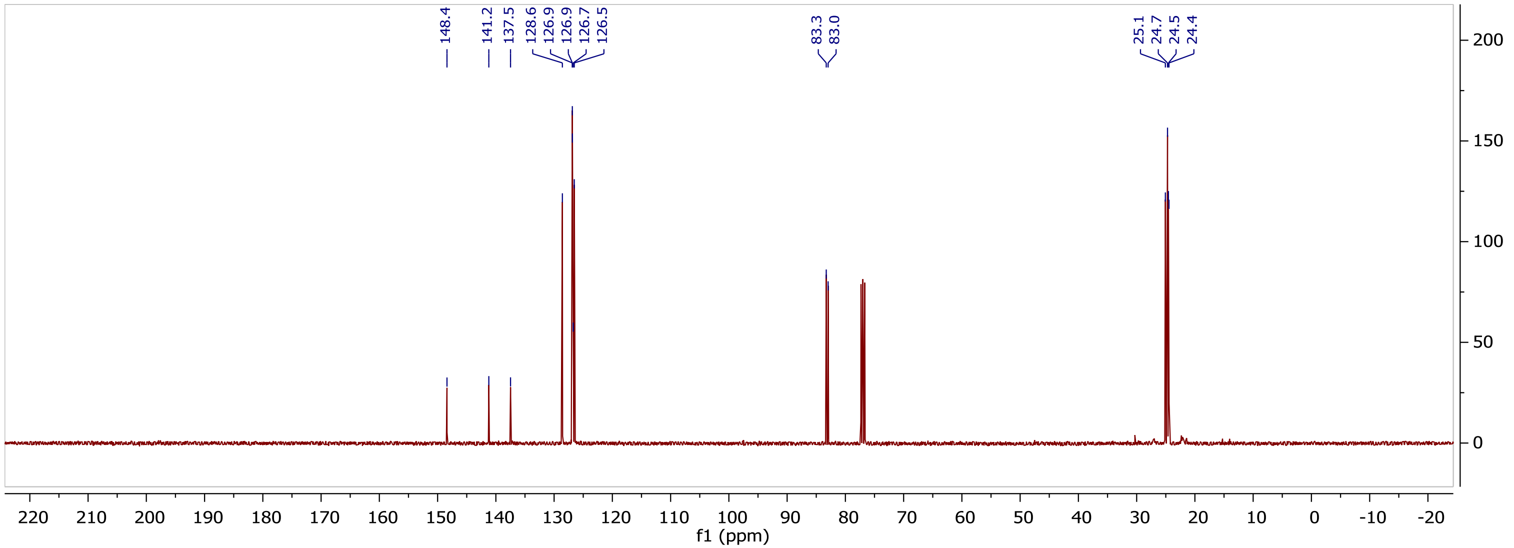

(*S*)-2,2'-(2-(4-chlorophenyl)propane-1,2-diyl)bis(4,4,5,5-tetramethyl-1,3,2-dioxaborolane) (13)

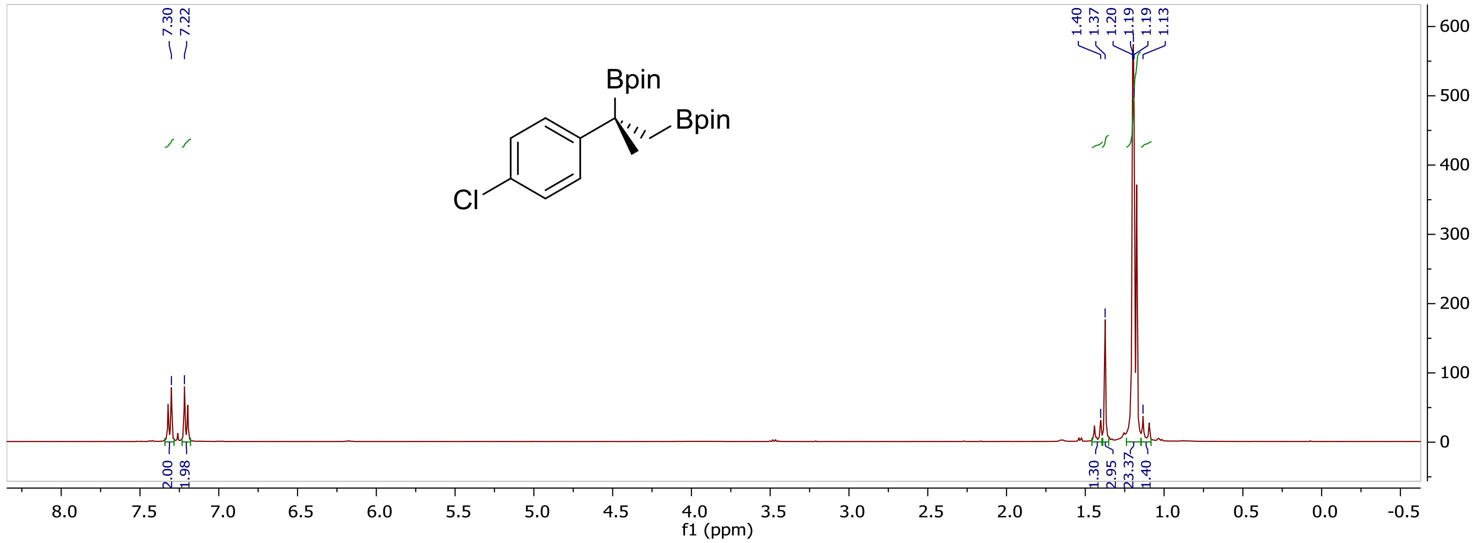

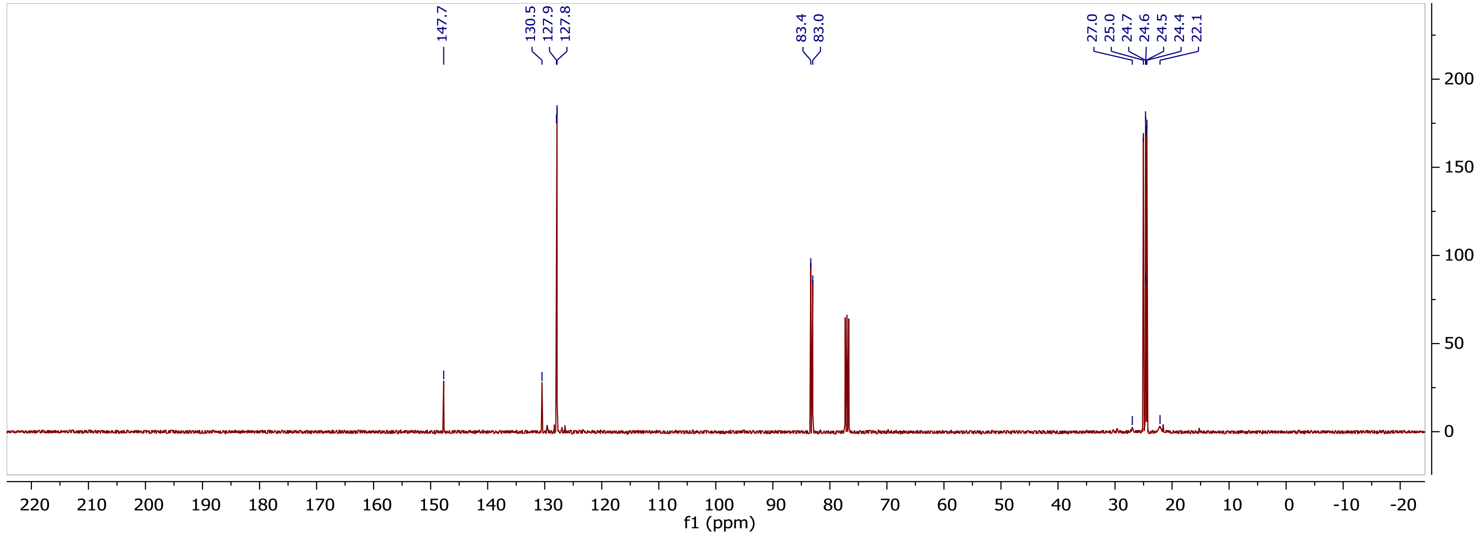

(*S*)-2,2'-(2-(*p*-tolyl)propane-1,2-diyl)bis(4,4,5,5-tetramethyl-1,3,2-dioxaborolane) (14)

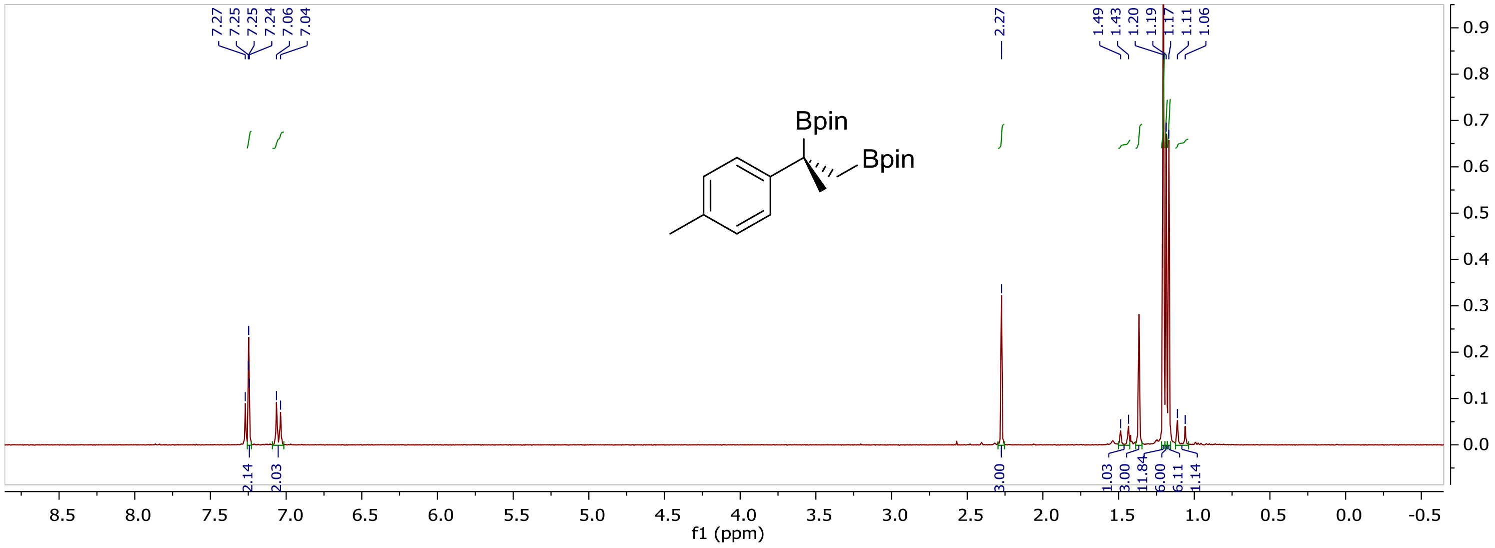

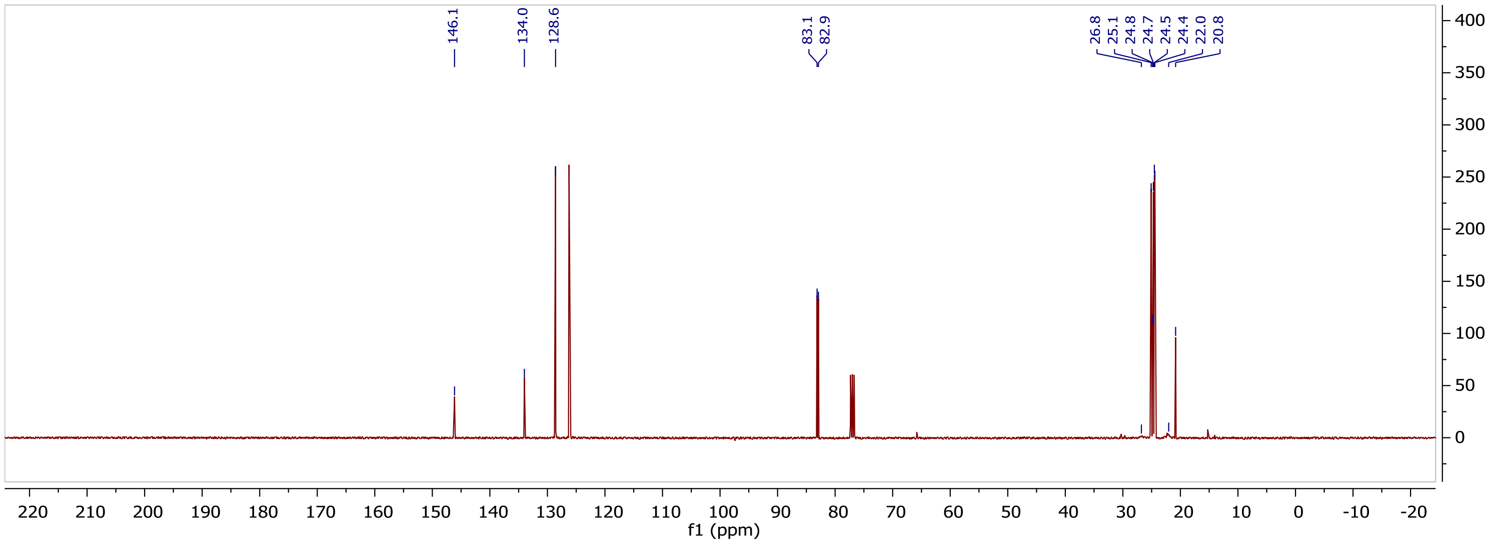

(*S*)-2,2'-(2-(4-methoxyphenyl)propane-1,2-diyl)bis(4,4,5,5-tetramethyl-1,3,2-dioxaborolane) (15)

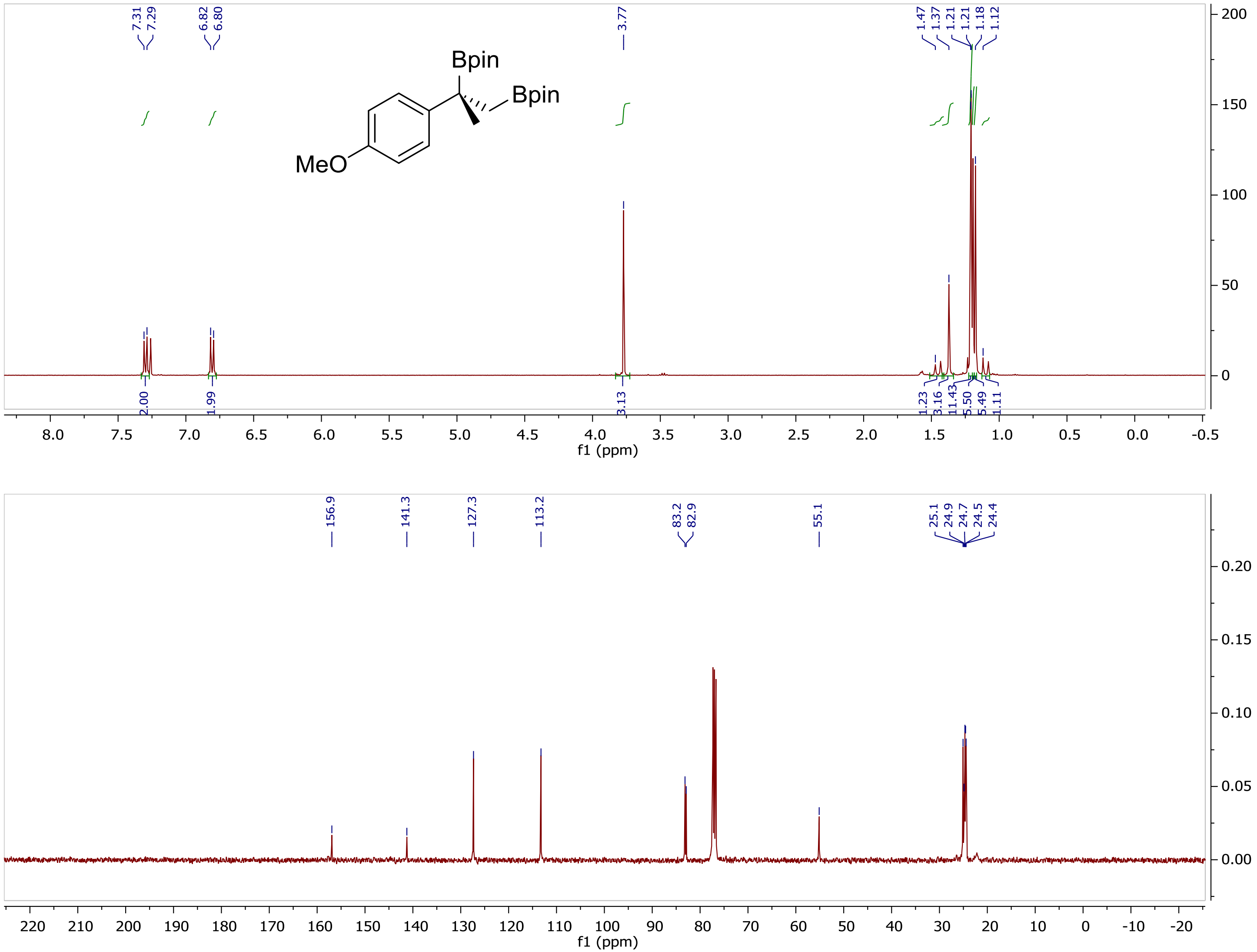

(*S*)-2,2'-(2-(4-fluorophenyl)propane-1,2-diyl)bis(4,4,5,5-tetramethyl-1,3,2-dioxaborolane) (16)

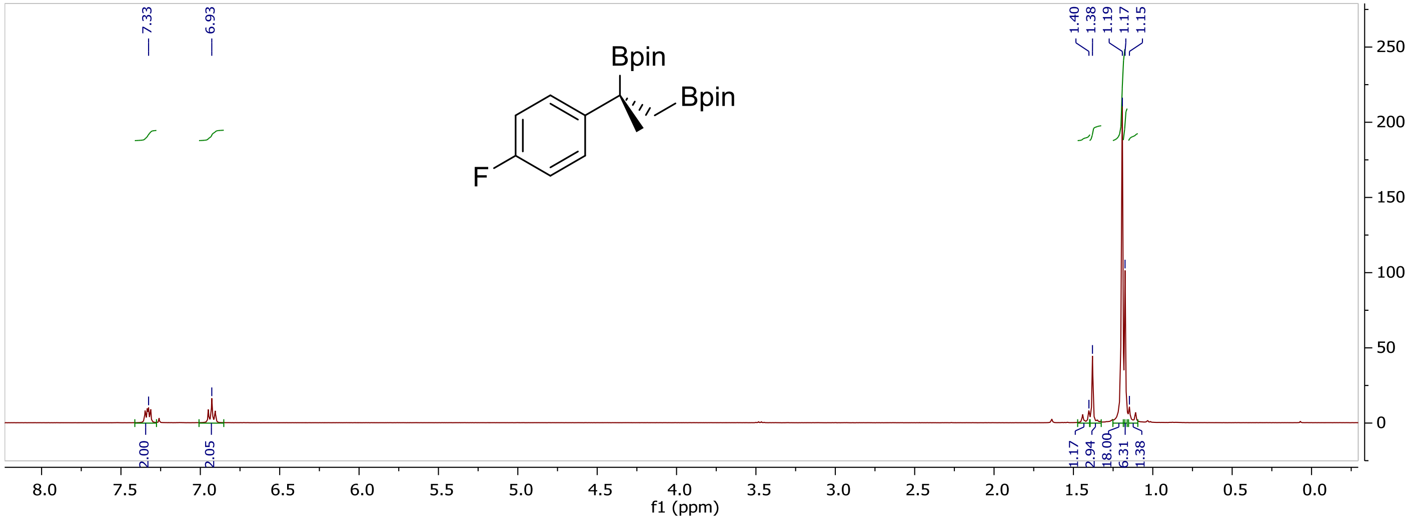

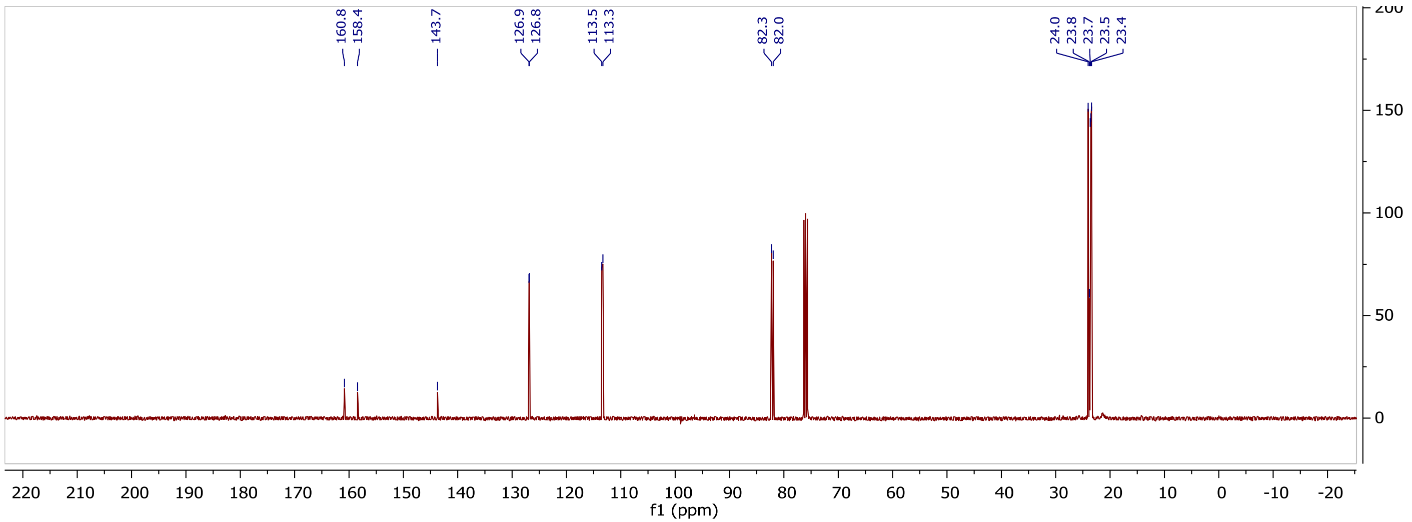

(*S*)-2,2'-(2-(2-fluorophenyl)propane-1,2-diyl)bis(4,4,5,5-tetramethyl-1,3,2-dioxaborolane) (17)

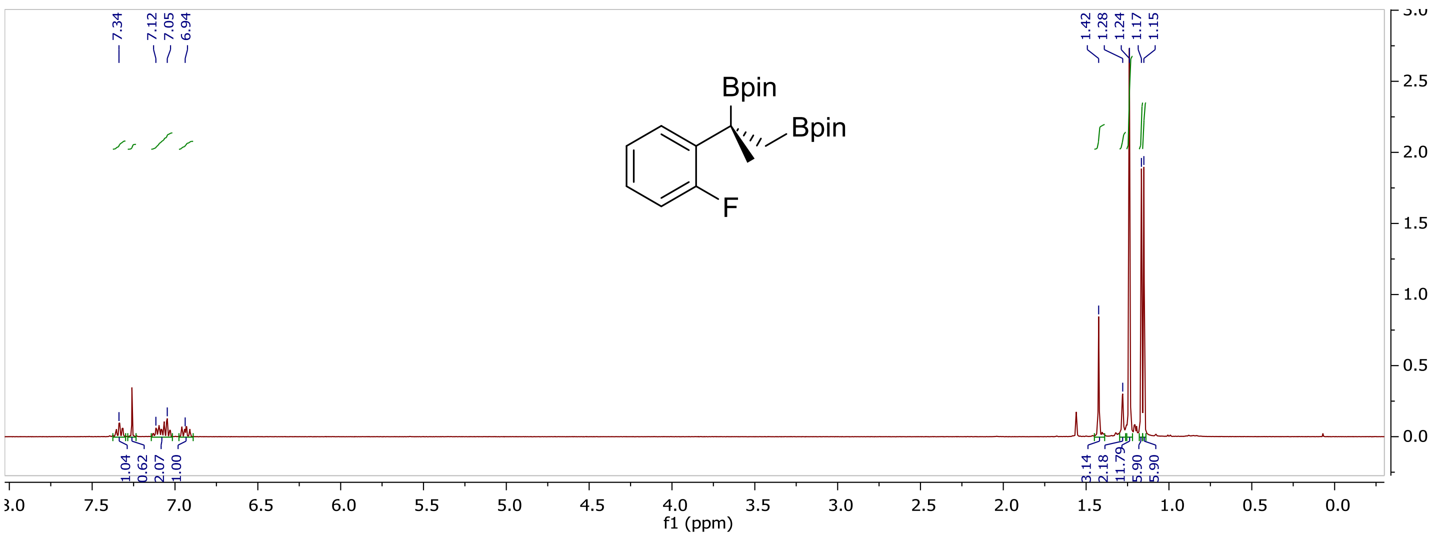

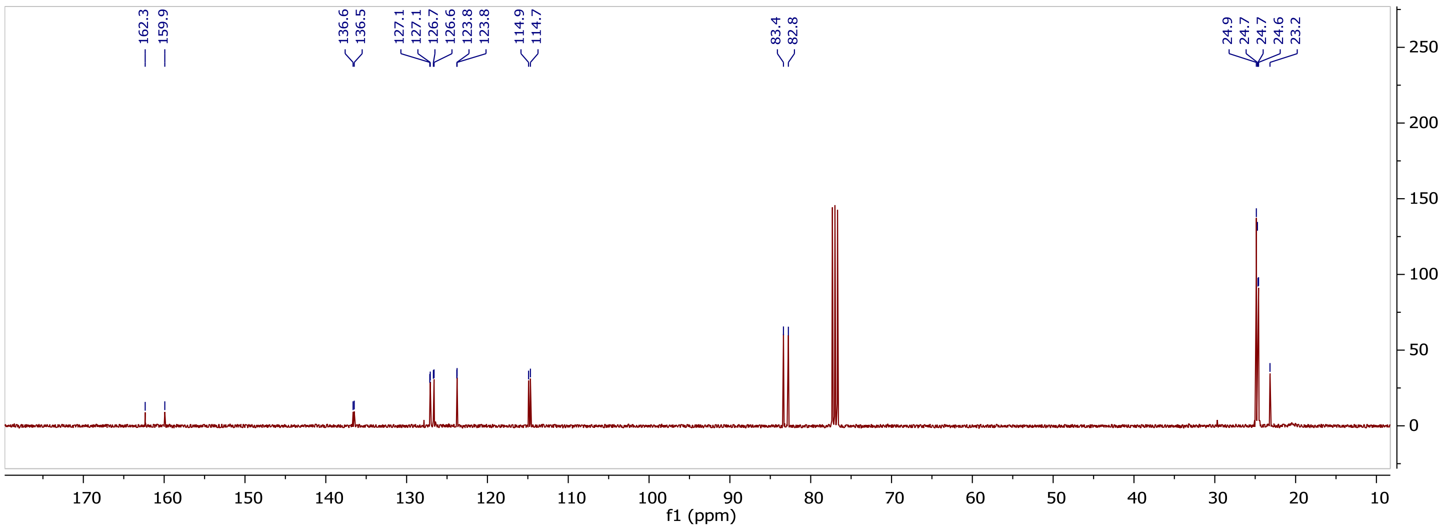

(*S*)-2,2'-(2-(2-methoxyphenyl)propane-1,2-diyl)bis(4,4,5,5-tetramethyl-1,3,2-dioxaborolane) (18)

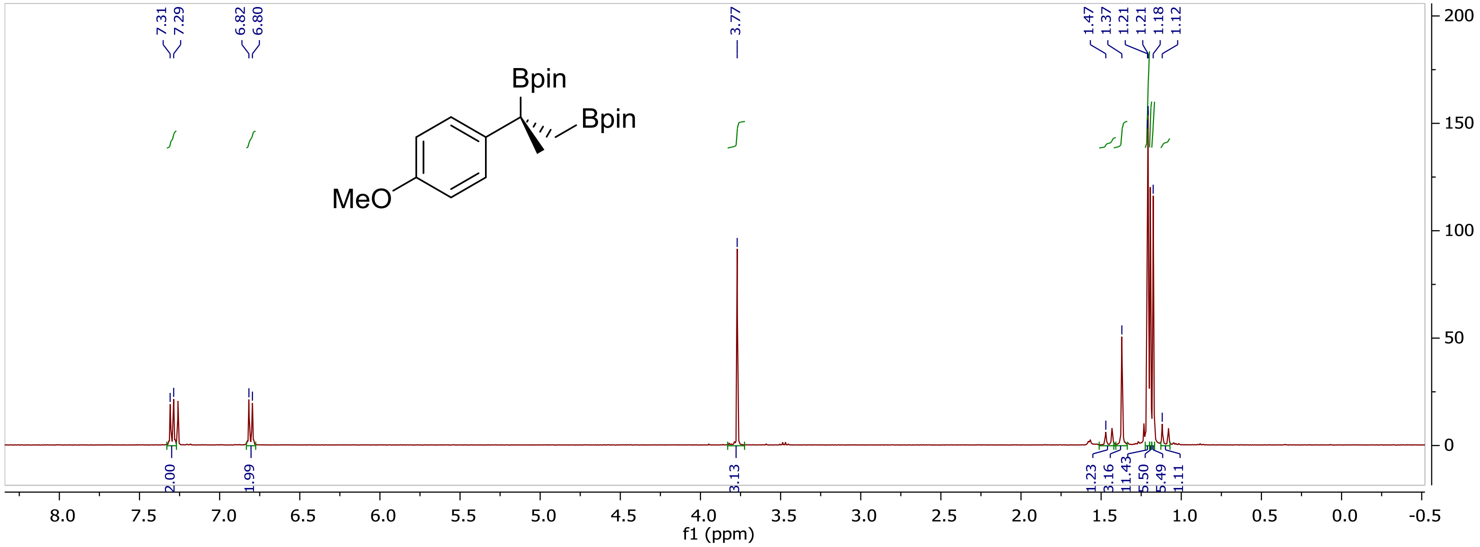

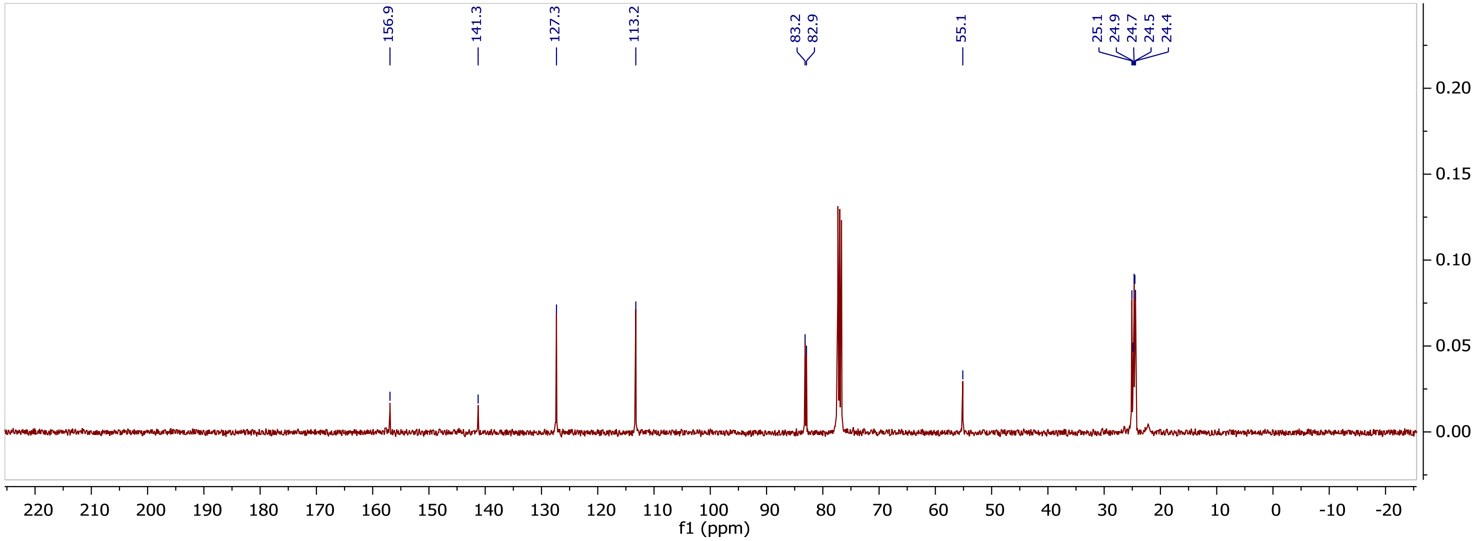

(*R*)-2,2'-(2-methyl-4-phenylbutane-1,2-diyl)bis(4,4,5,5-tetramethyl-1,3,2-dioxaborolane) (20)

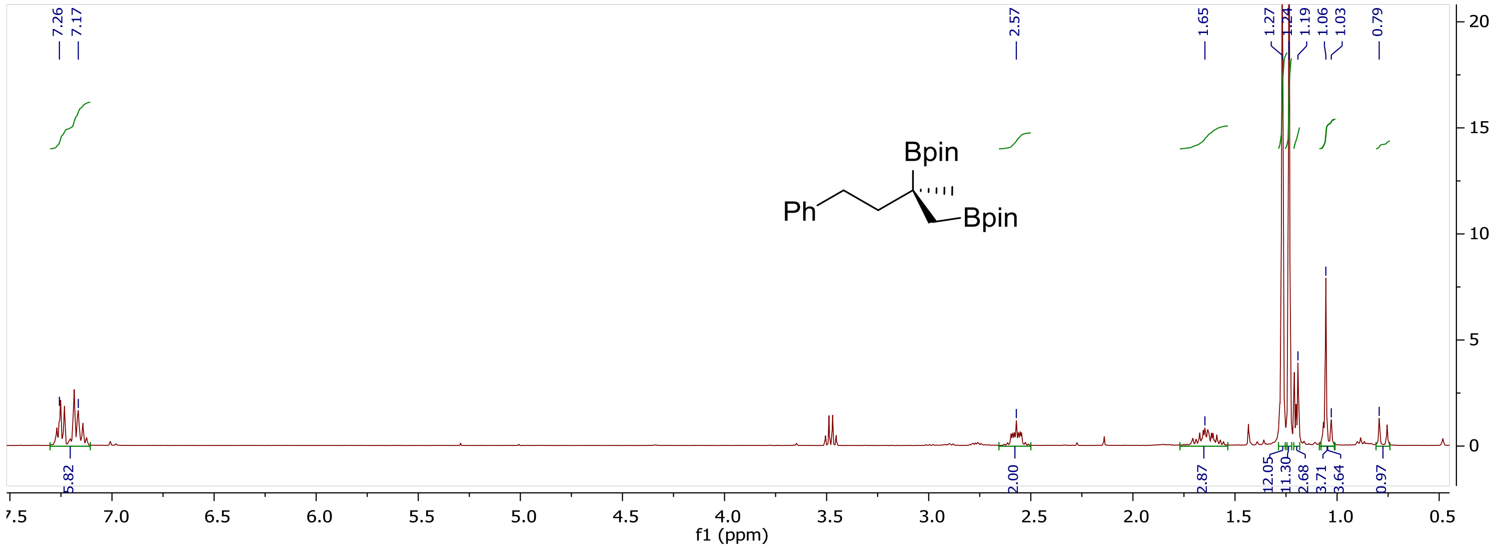

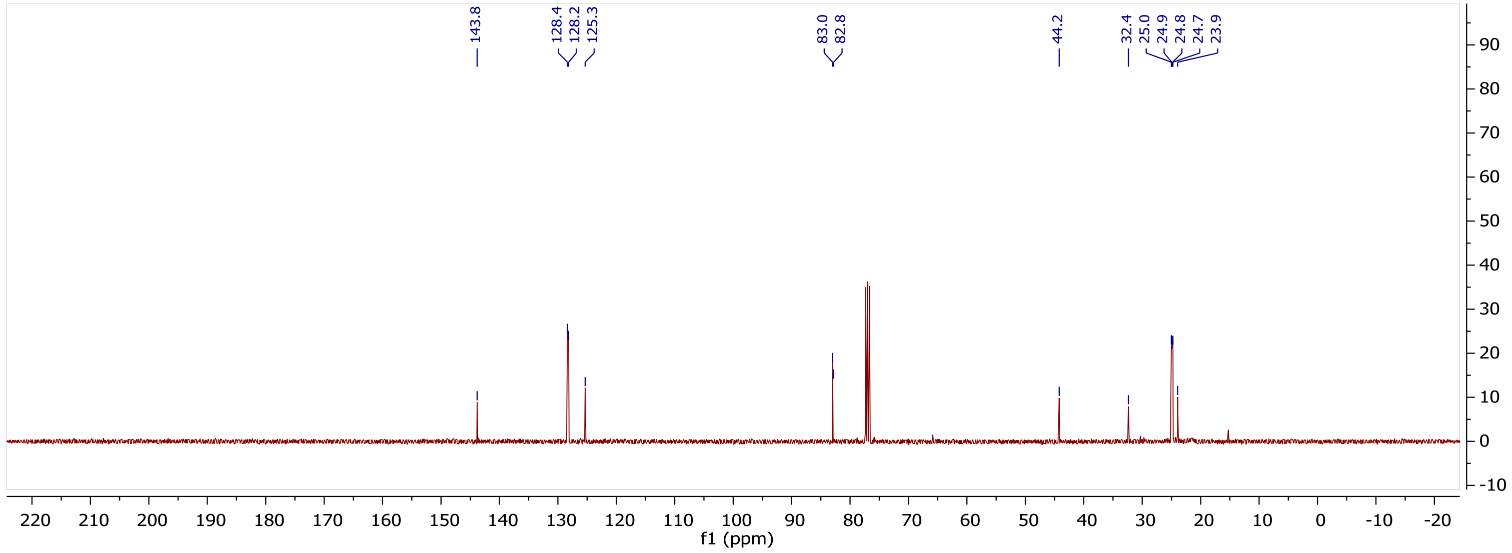

2,2'-((3*S*,5*S*)-1,7-bis(4-methoxyphenyl)heptane-3,5-diyl)bis(4,4,5,5-tetramethyl-1,3,2-dioxaborolane) (4)

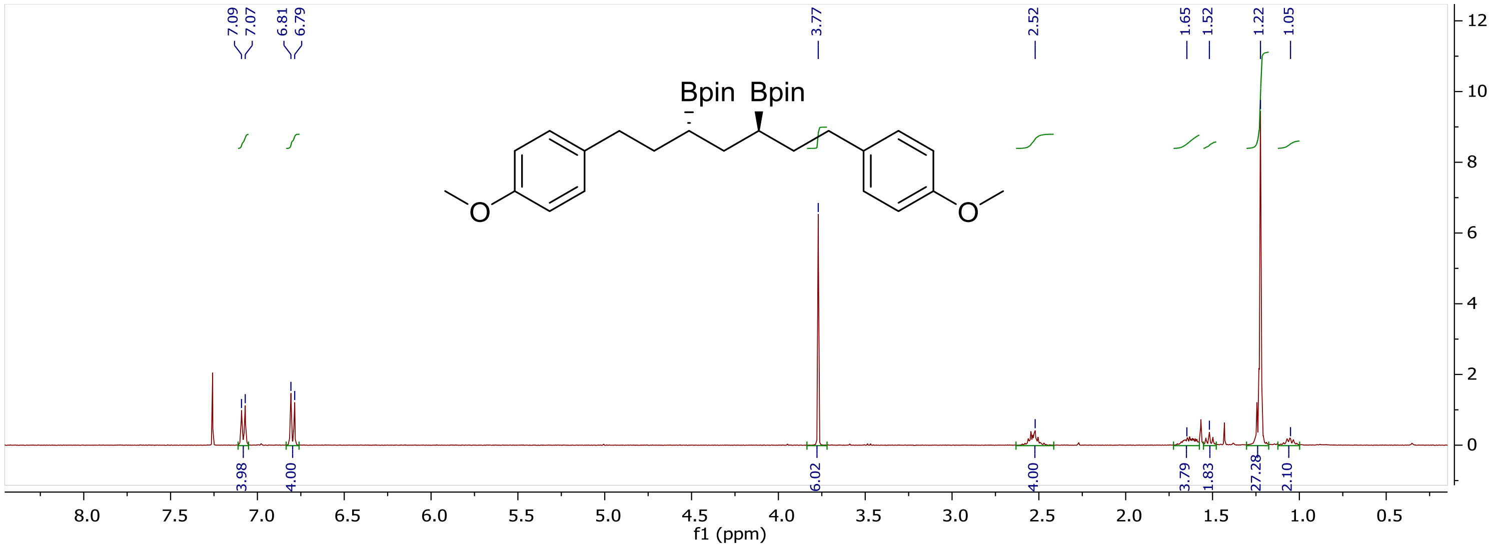

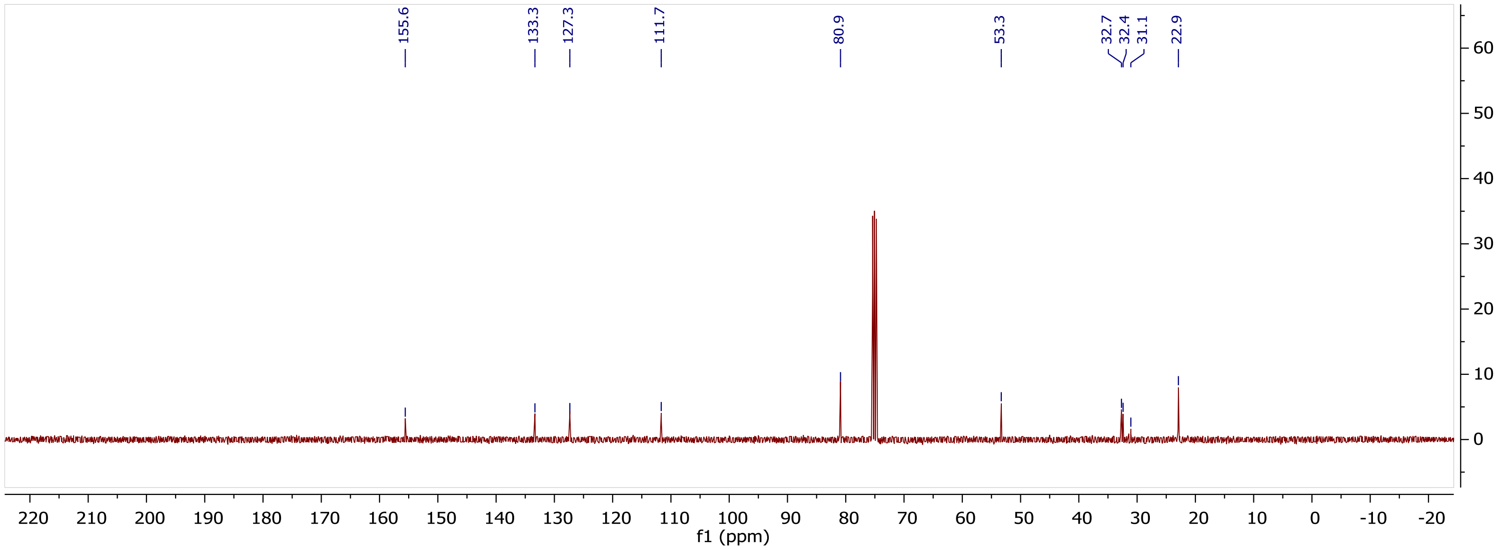

2,2'-((2*S*,4*S*)-pentane-2,4-diyl)bis(4,4,5,5-tetramethyl-1,3,2-dioxaborolane) (21)

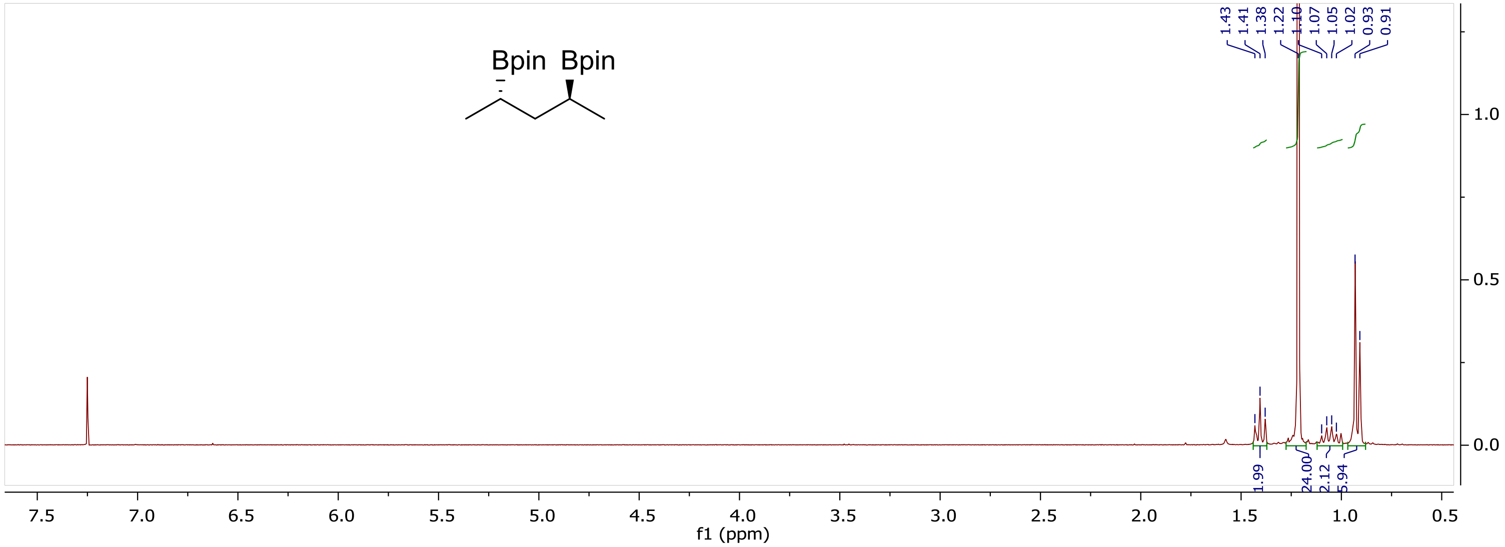

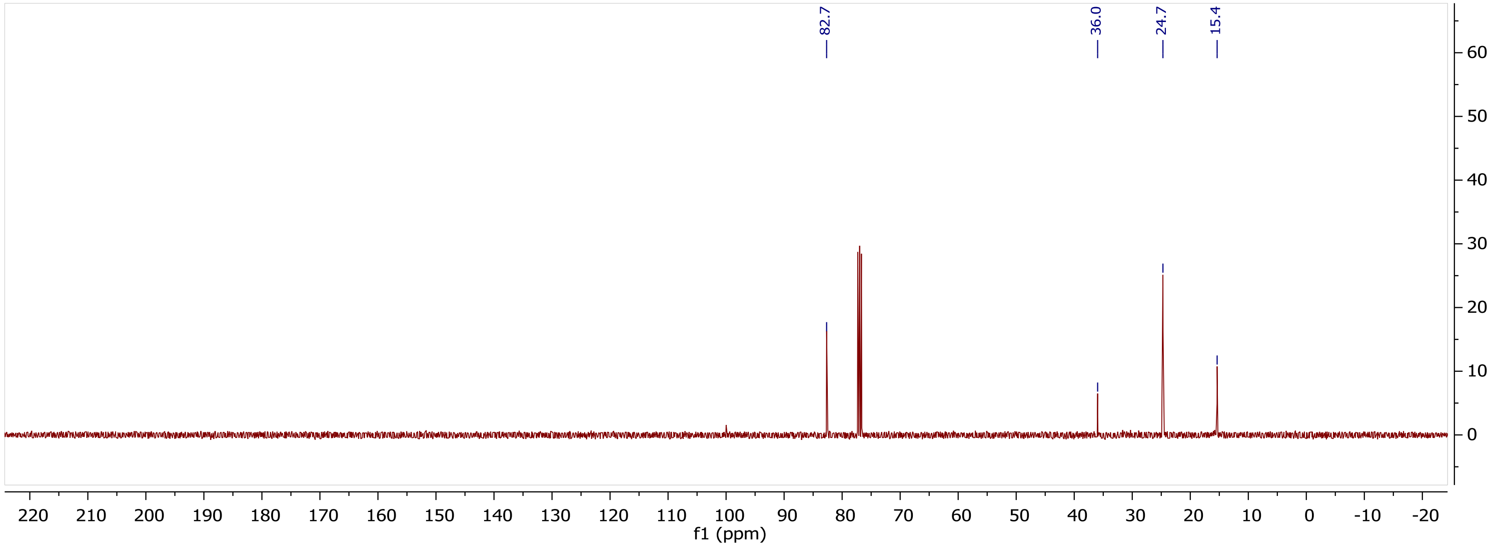

2,2'-((3*R*,5*R*)-2,6-dimethylheptane-3,5-diyl)bis(4,4,5,5-tetramethyl-1,3,2-dioxaborolane) (22)

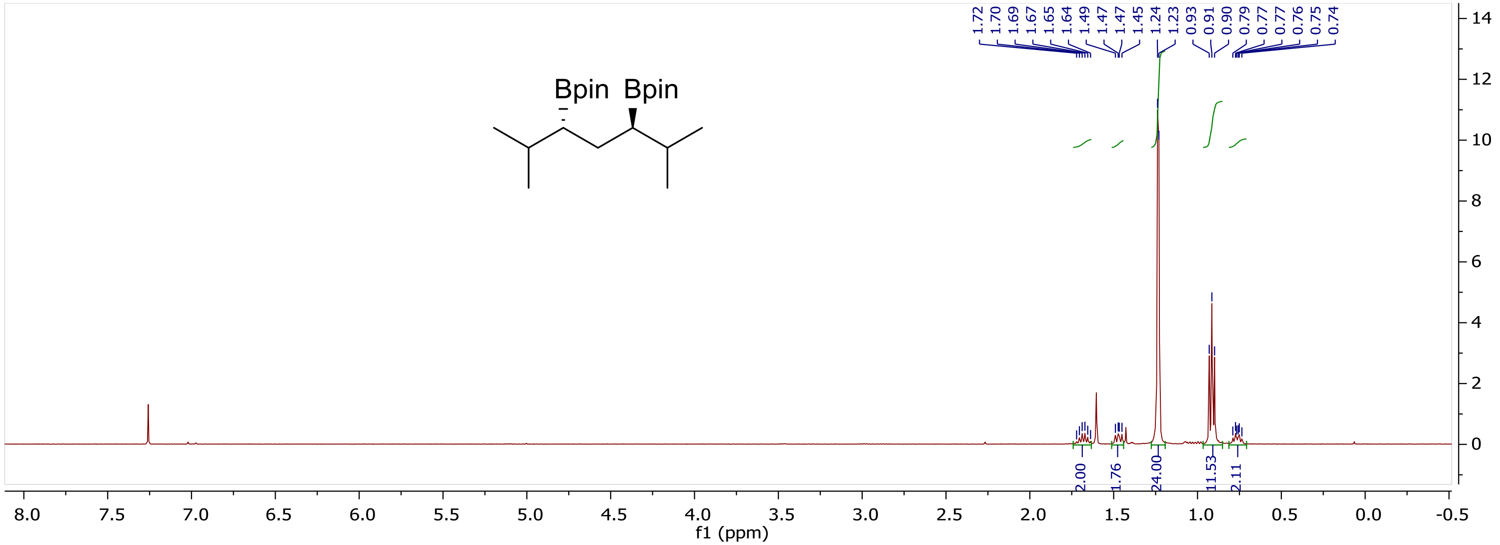

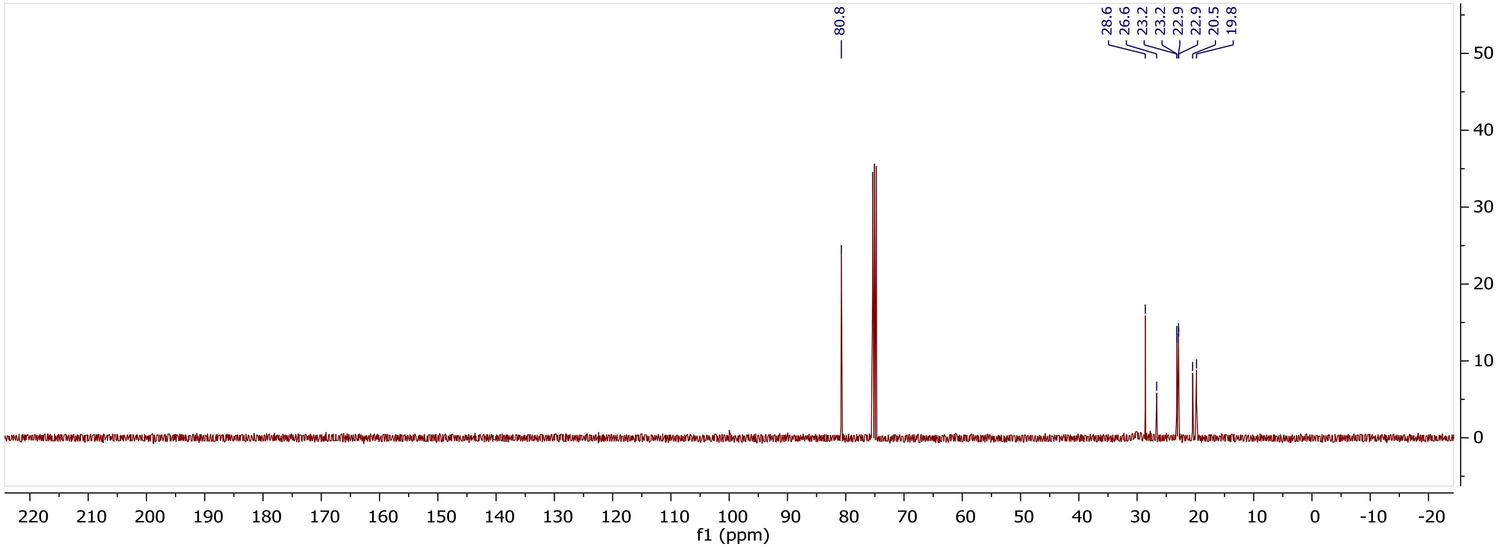

2,2'-((5S,7S)-undeca-1,10-diene-5,7-diyl)bis(4,4,5,5-tetramethyl-1,3,2-dioxaborolane) (23)

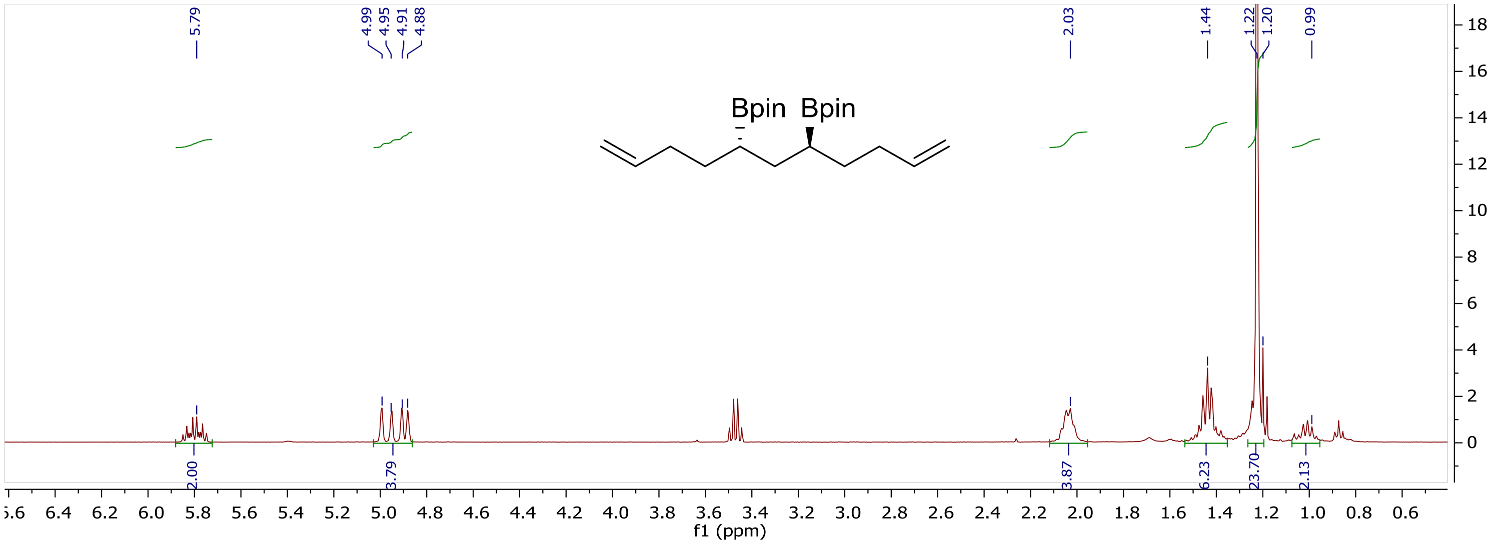

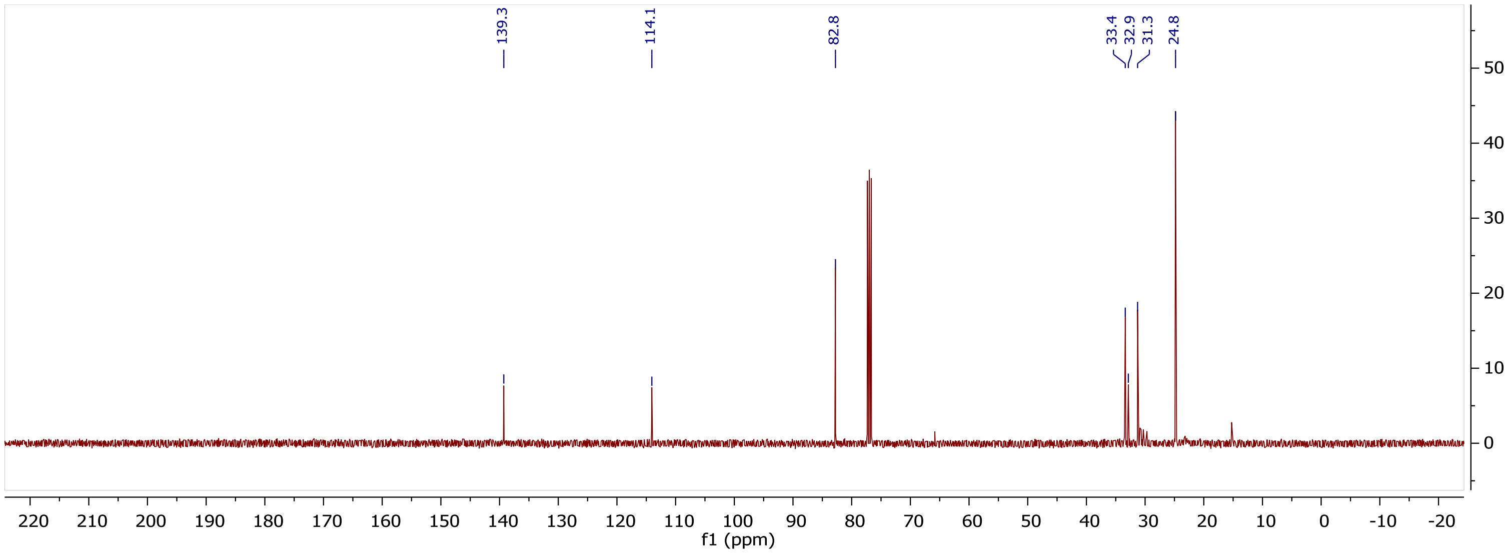

(6*R*,8*R*)-2,2,12,12-tetramethyl-6,8-bis(4,4,5,5-tetramethyl-1,3,2-dioxaborolan-2-yl)-3,11-dioxa-2,12-disilatridecane (24)

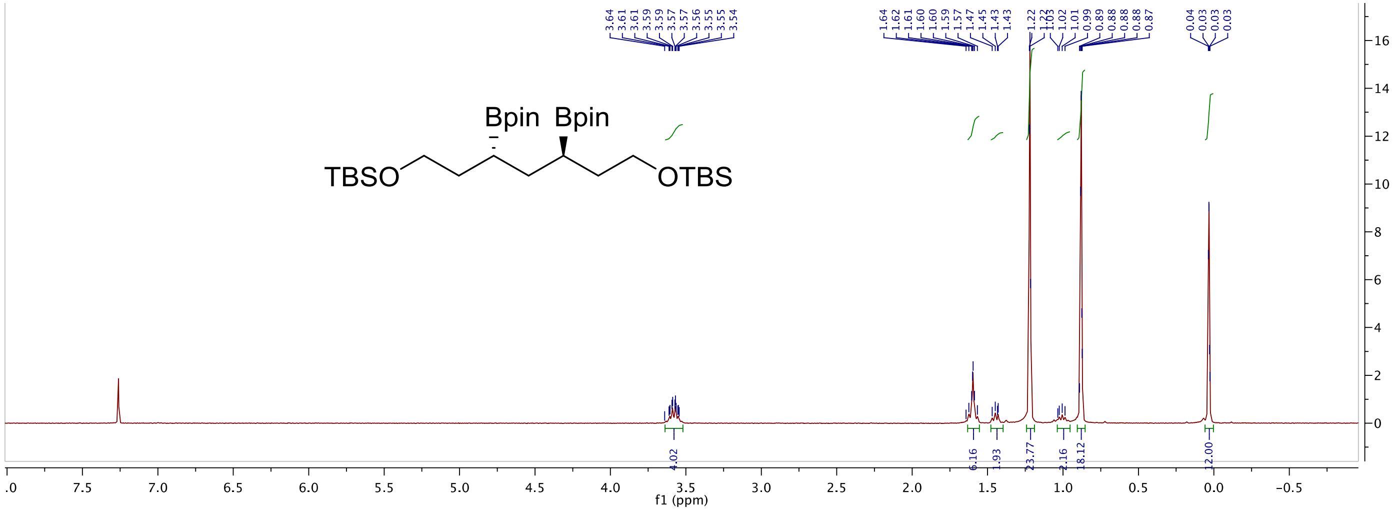

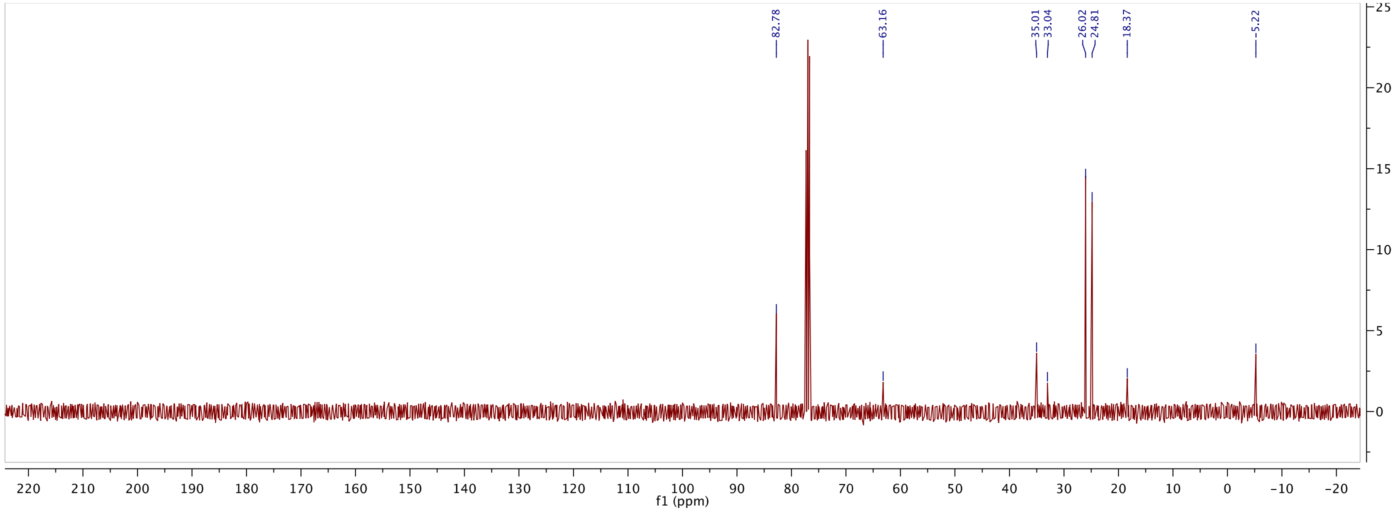

2,2'-((2*S*,4*S*)-2,4-bis(4-methoxyphenyl)pentane-2,4-diyl)bis(4,4,5,5-tetramethyl-1,3,2-dioxaborolane) (25)

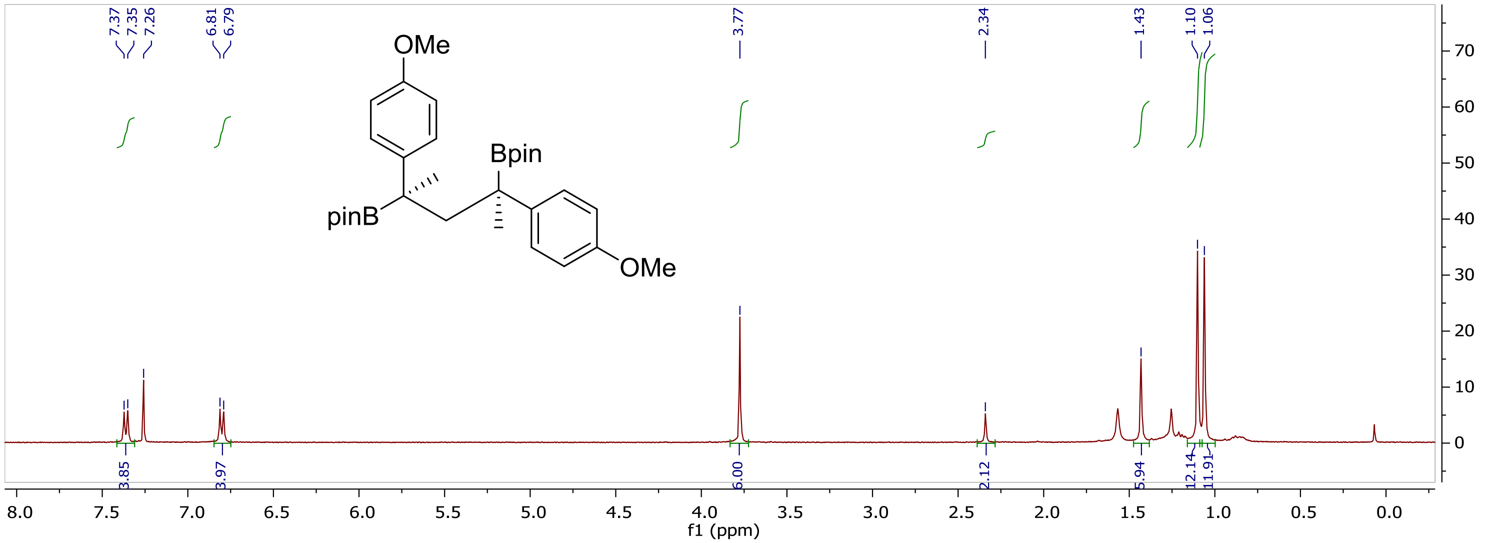

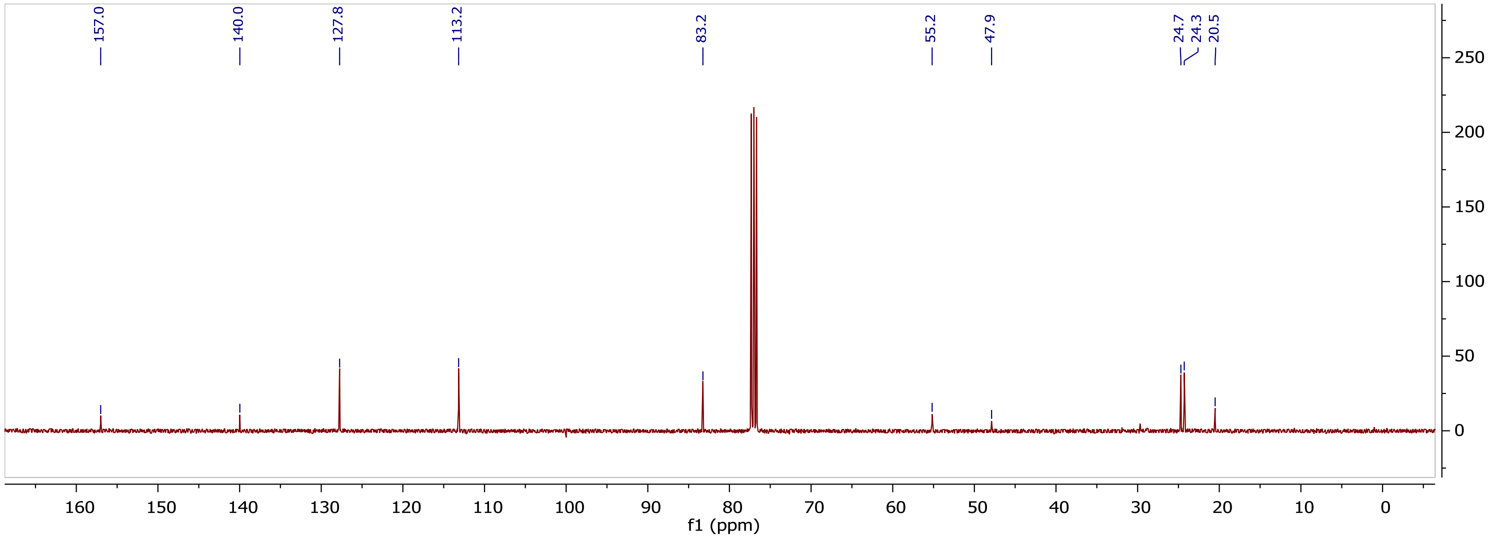

2,2'-((2*S*,4*R*)-2-(4-methoxyphenyl)pentane-2,4-diyl)bis(4,4,5,5-tetramethyl-1,3,2-dioxaborolane) (26)

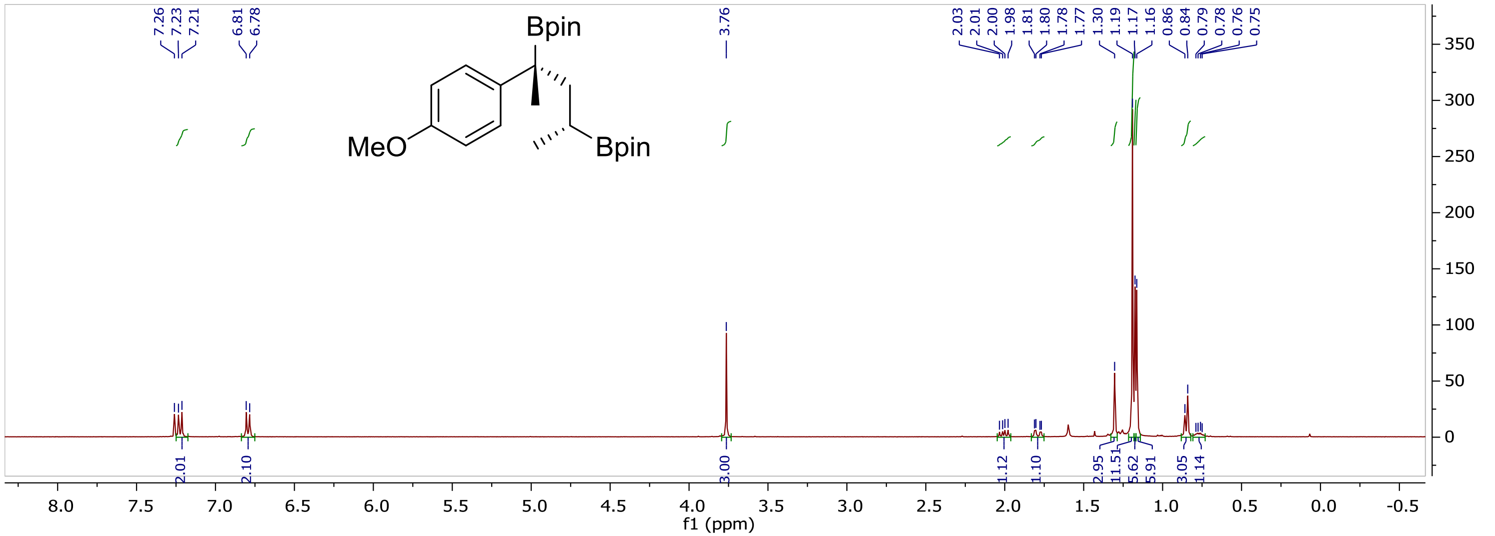

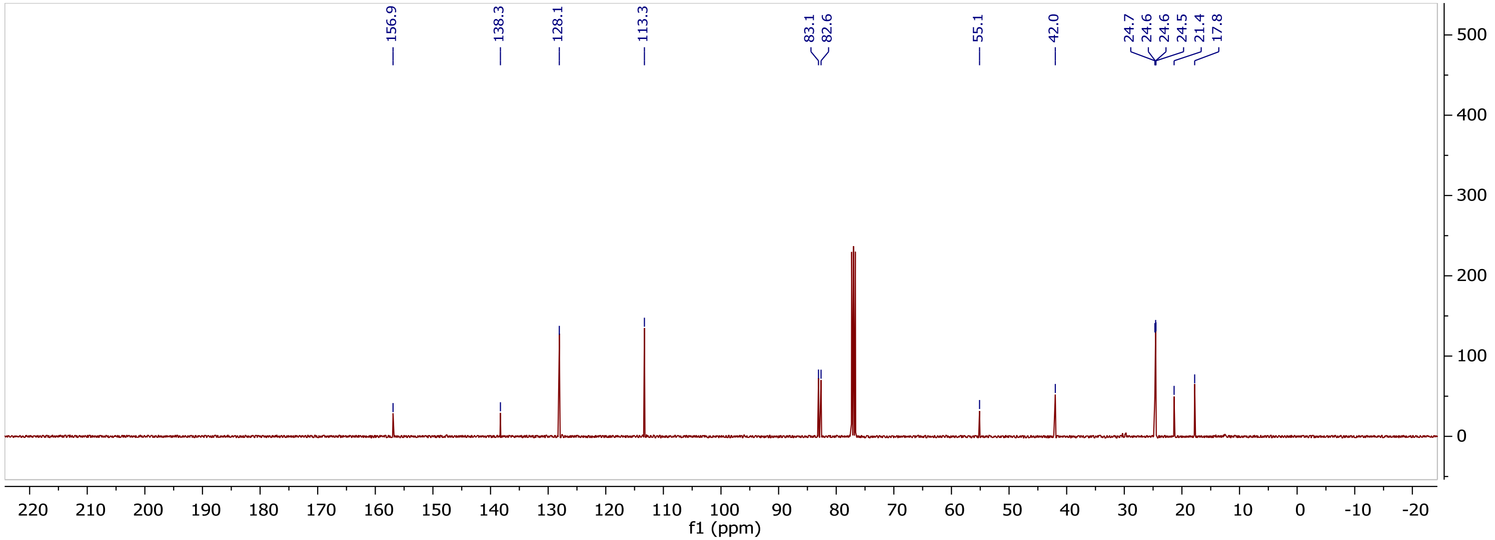

2,2'-((2*R*,3*S*,5*R*)-3-(4-methoxyphenyl)-3-methylhexane-2,5-diyl)bis(4,4,5,5-tetramethyl-1,3,2-dioxaborolane) (27)

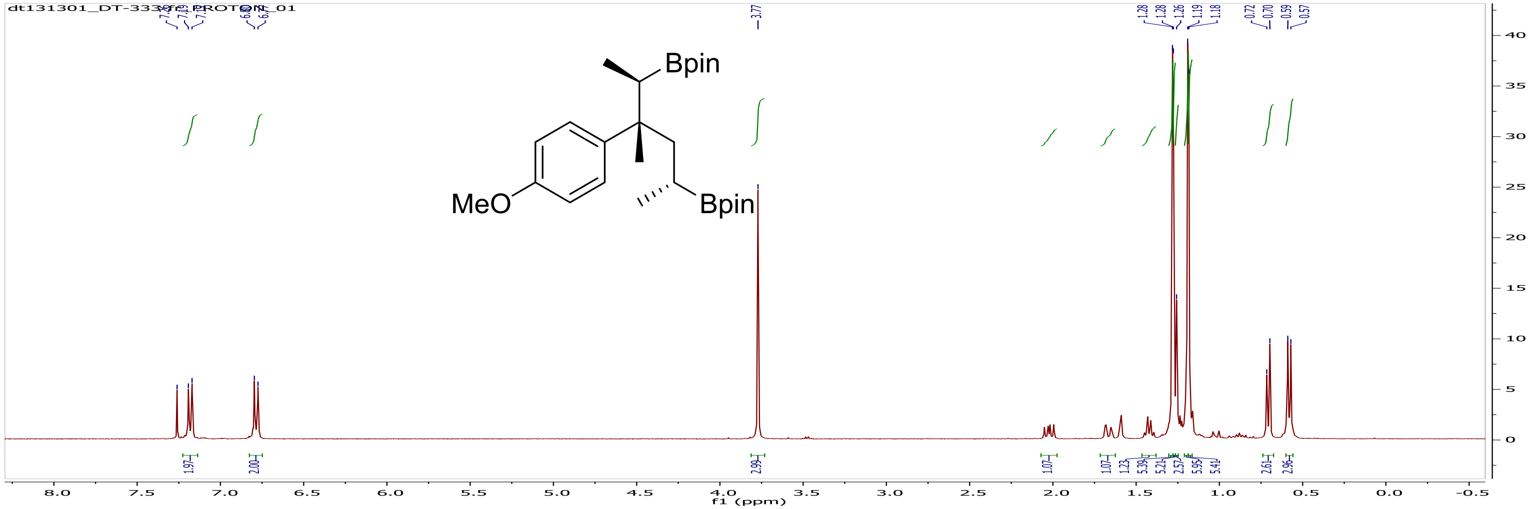

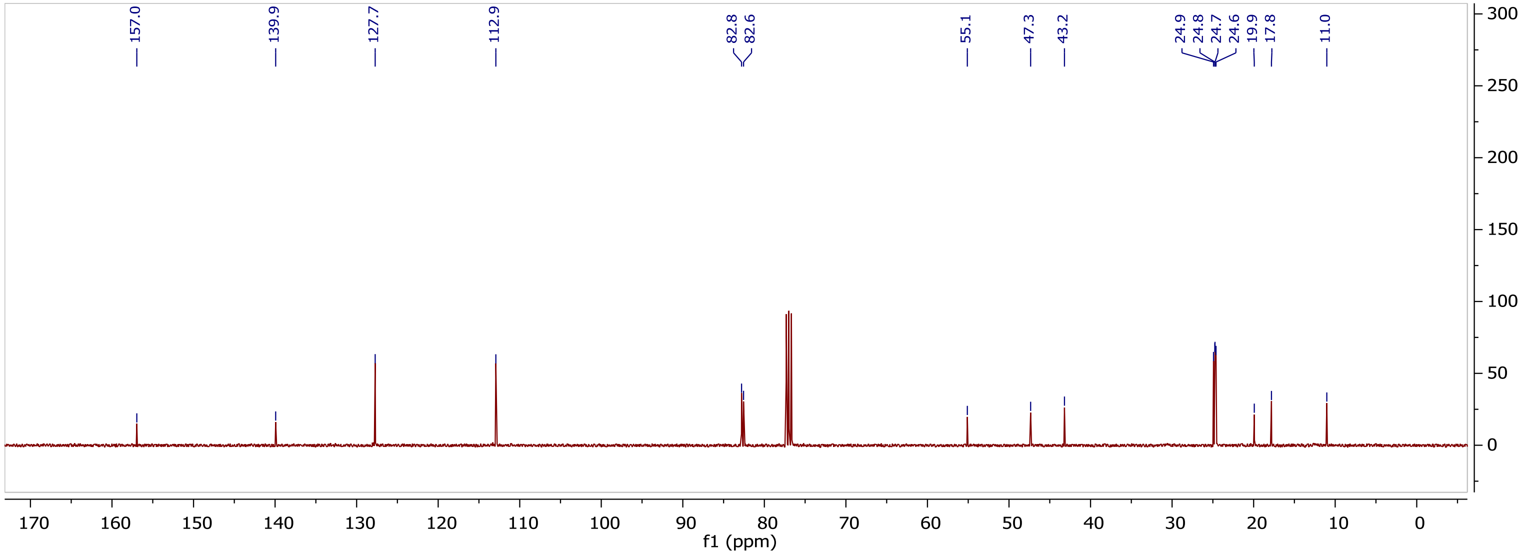

2,2'-((2*S*,4*S*)-6-(4-methoxyphenyl)hexane-2,4-diyl)bis(4,4,5,5-tetramethyl-1,3,2-dioxaborolane) (31)

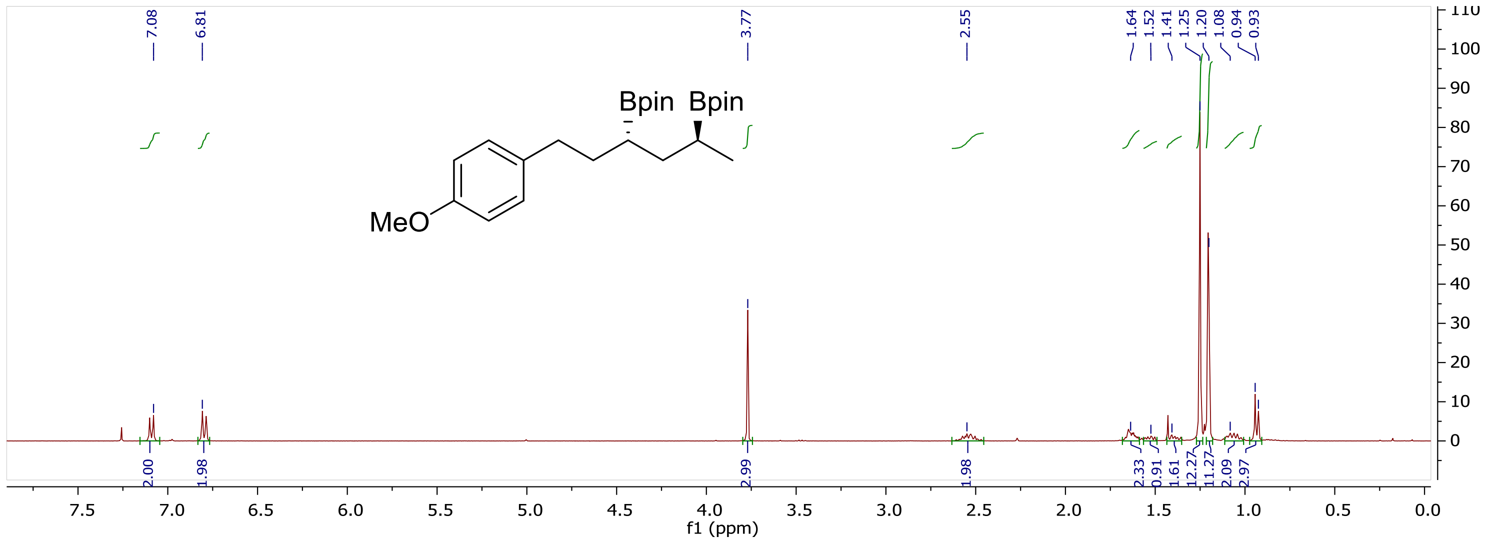

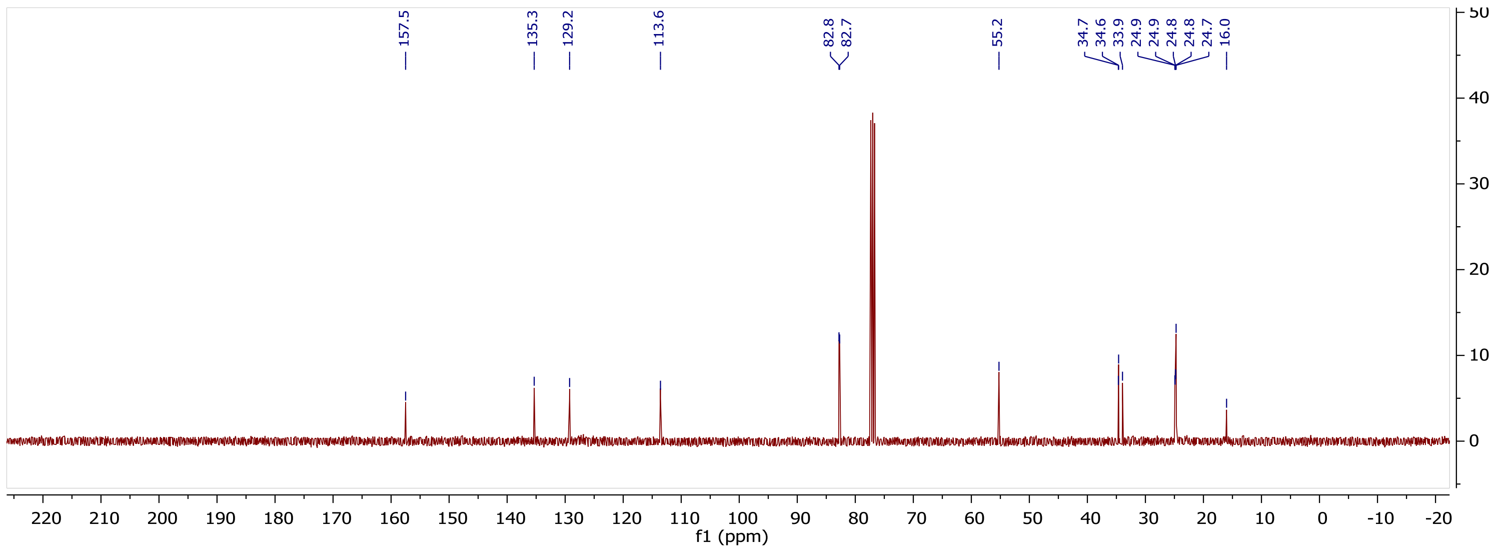

2,2'-((3*S*,5*S*)-1-(4-methoxyphenyl)-7-phenylheptane-3,5-diyl)bis(4,4,5,5-tetramethyl-1,3,2-dioxaborolane) (32)

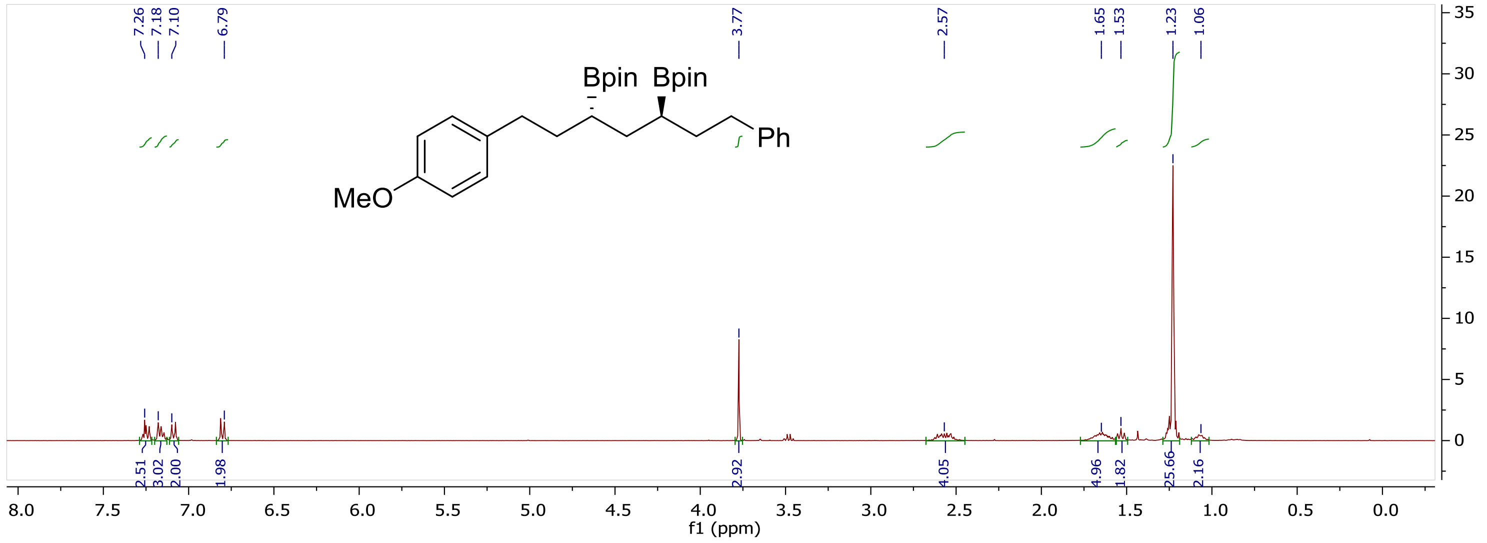

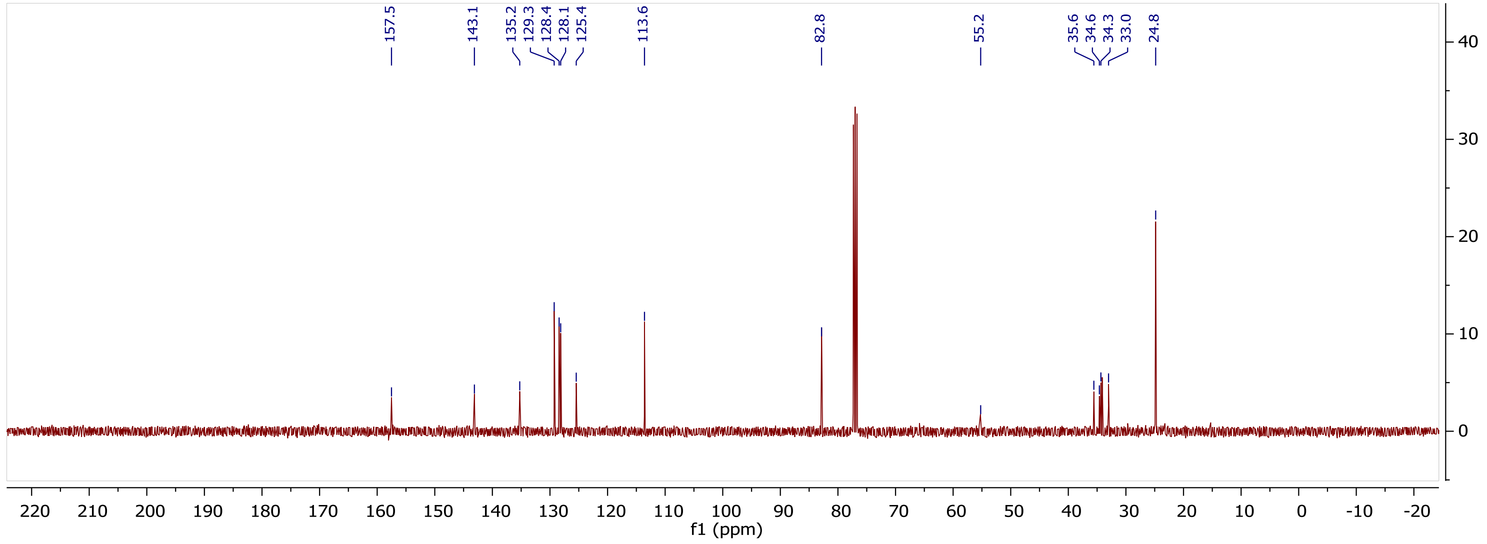

tert-butyl(((3_R_,5_S_)-7-(4-methoxyphenyl)-3,5-bis(4,4,5,5-tetramethyl-1,3,2-dioxaborolan-2-yl)heptyl)oxy)dimethylsilane (33)

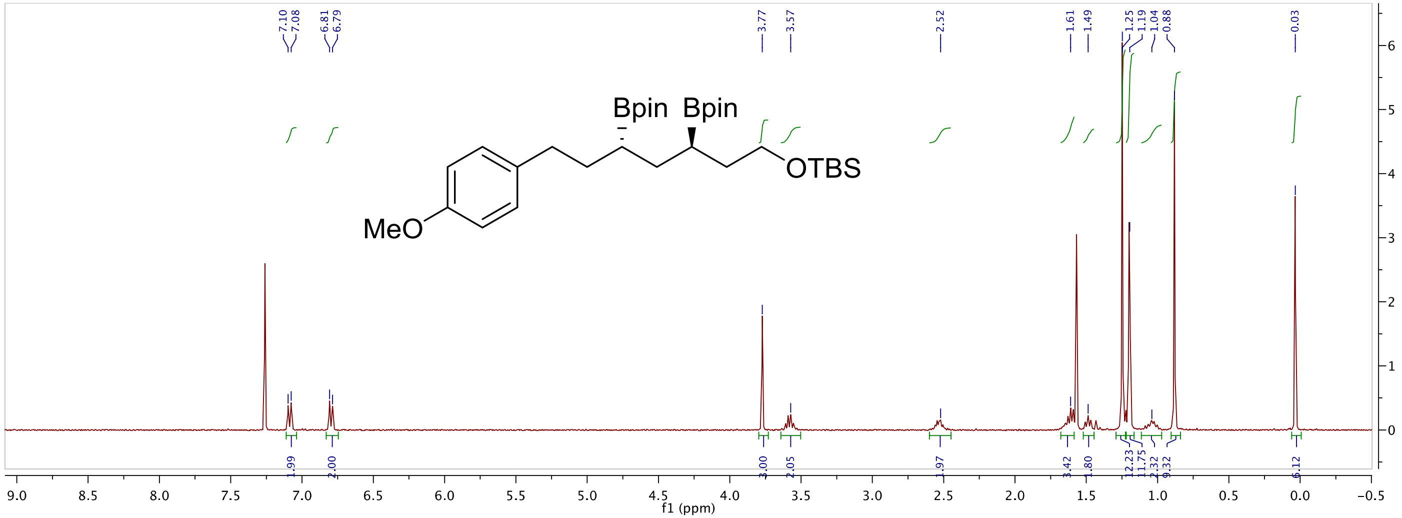

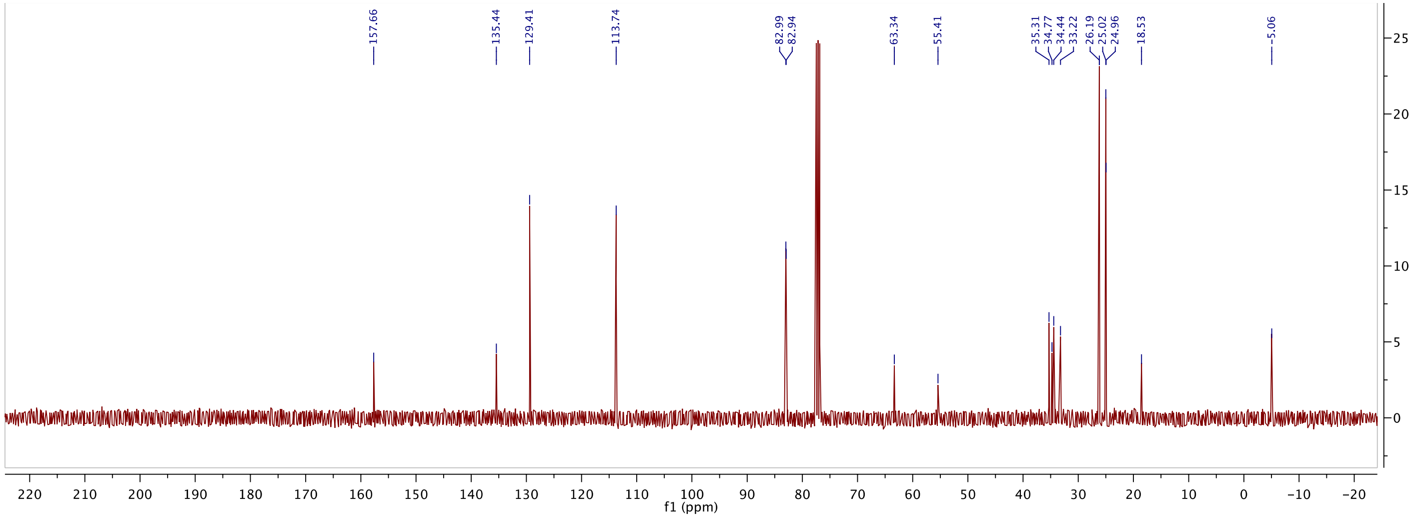

2,2'-((3*S*,5*S*)-1-(4-methoxyphenyl)non-8-ene-3,5-diyl)bis(4,4,5,5-tetramethyl-1,3,2-dioxaborolane) (34)

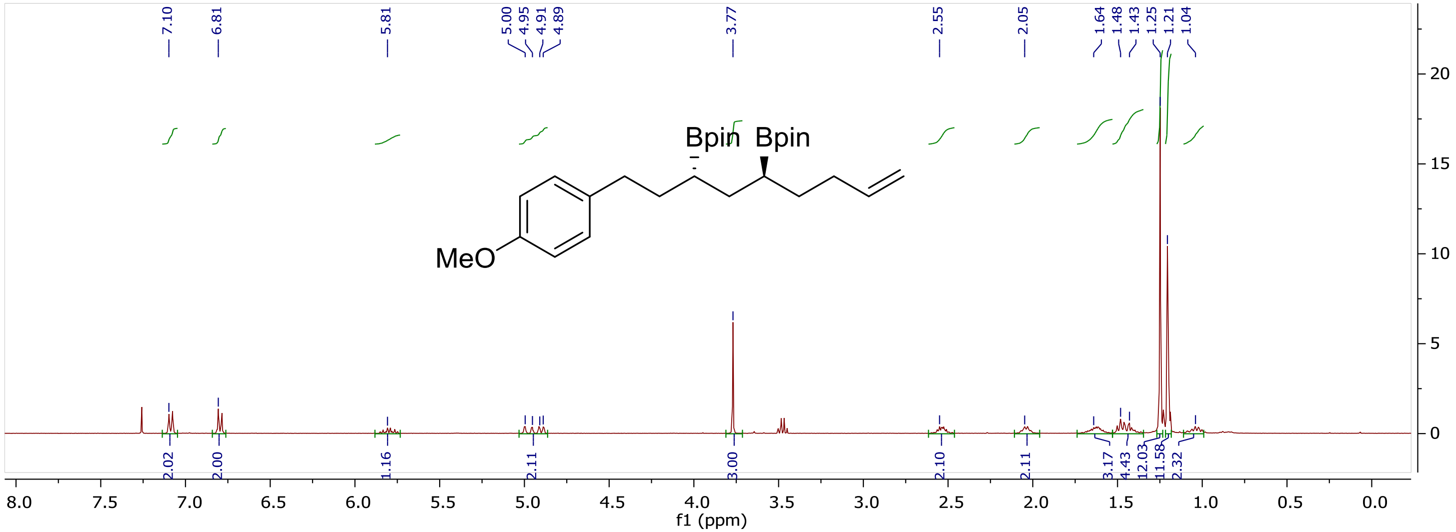

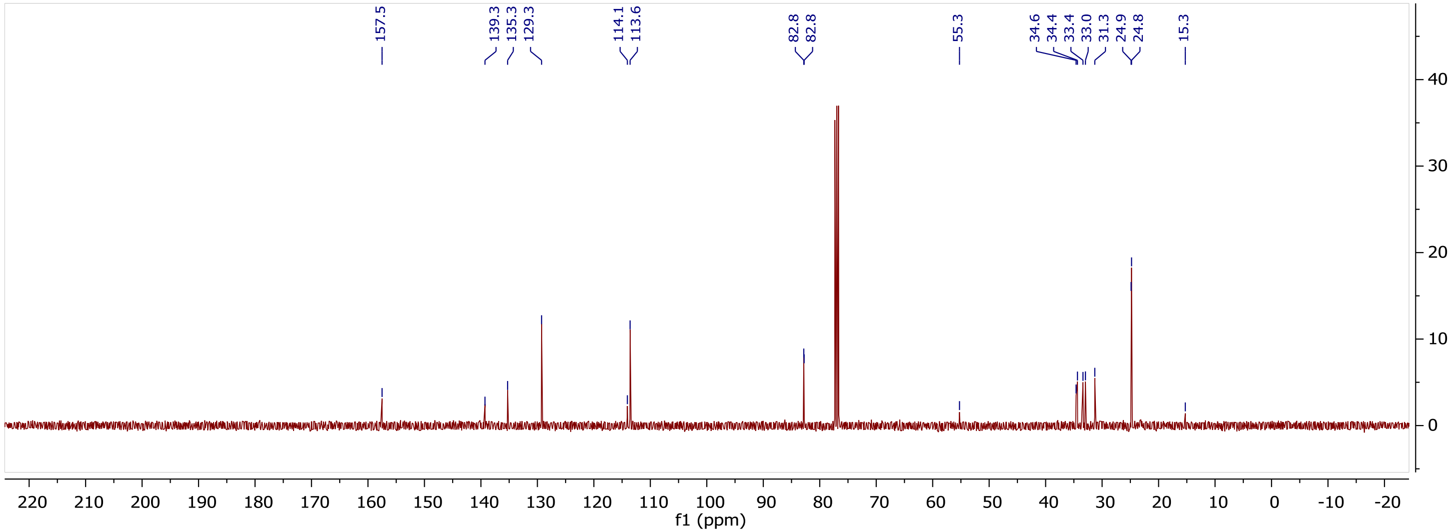

2,2'-((2*R*,4*S*)-6-(4-methoxyphenyl)hexane-2,4-diyl)bis(4,4,5,5-tetramethyl-1,3,2-dioxaborolane) (35)

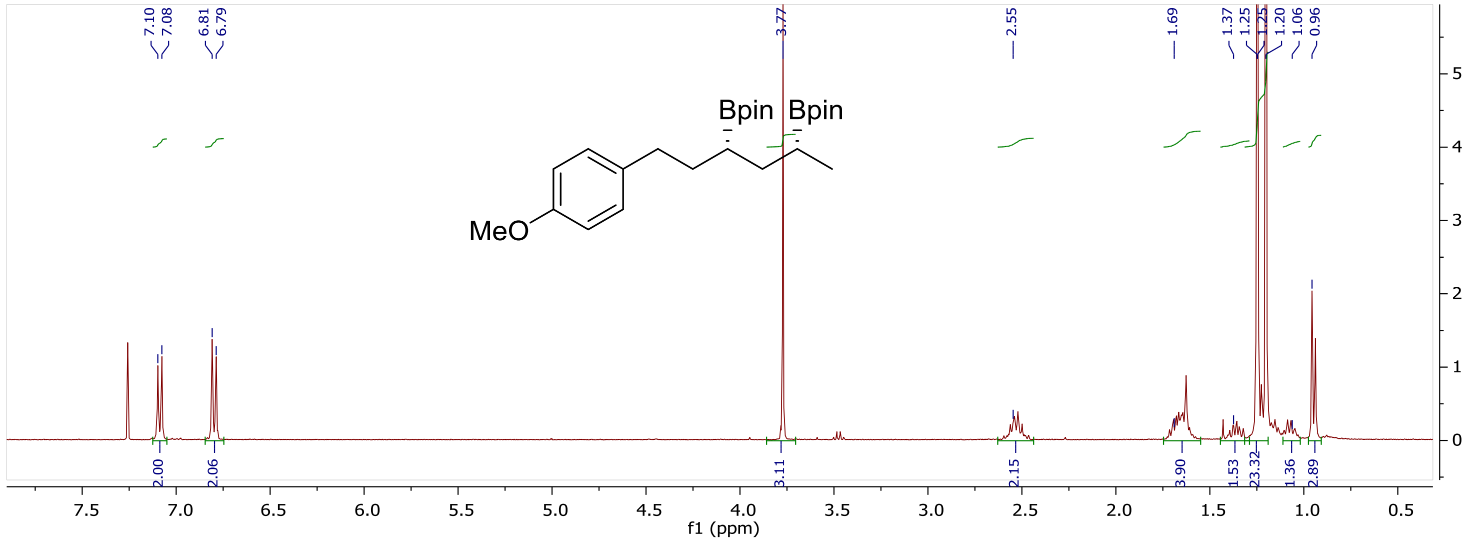

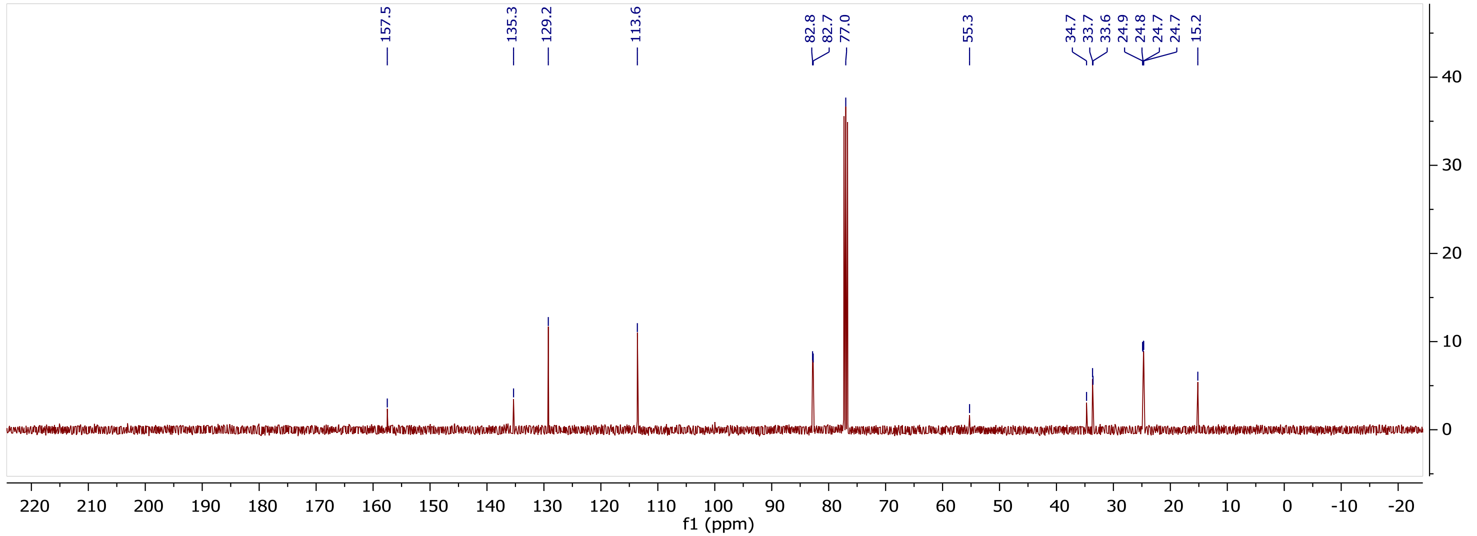

(*R*)-2,2'-(2-(4-methoxyphenyl)-2-methylbutane-1,4-diyl)bis(4,4,5,5-tetramethyl-1,3,2-dioxaborolane) (36)

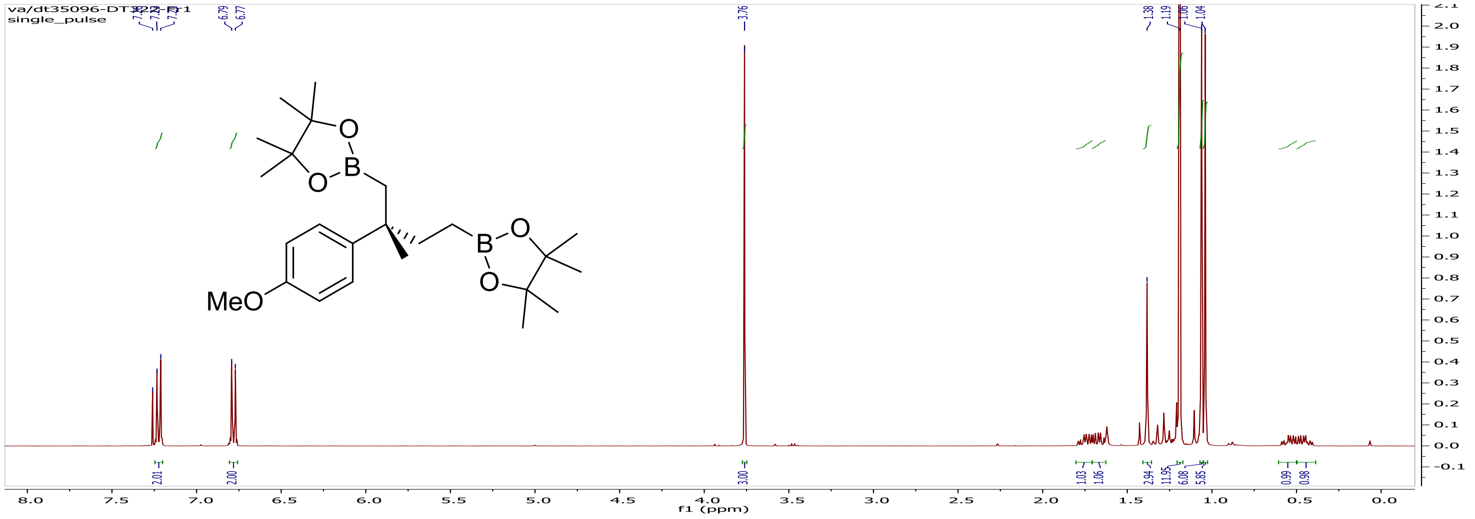

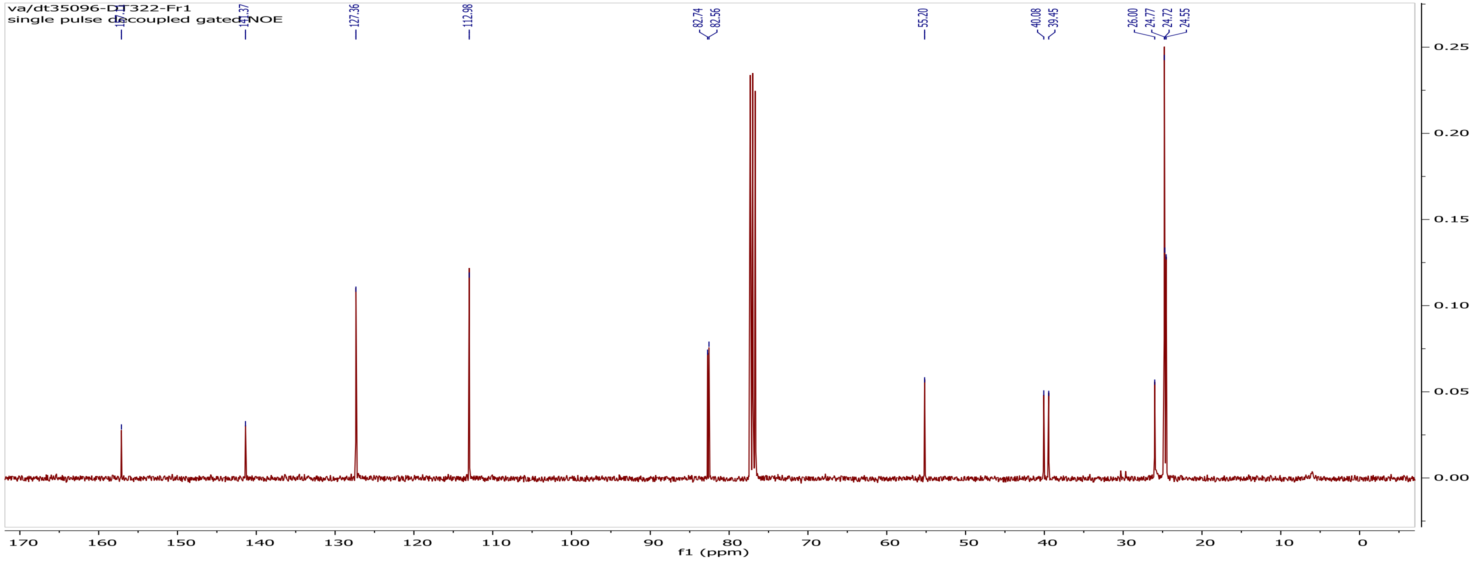

(*S*)-1-methoxy-4-(3-methylhexa-1,5-dien-3-yl)benzene (37)

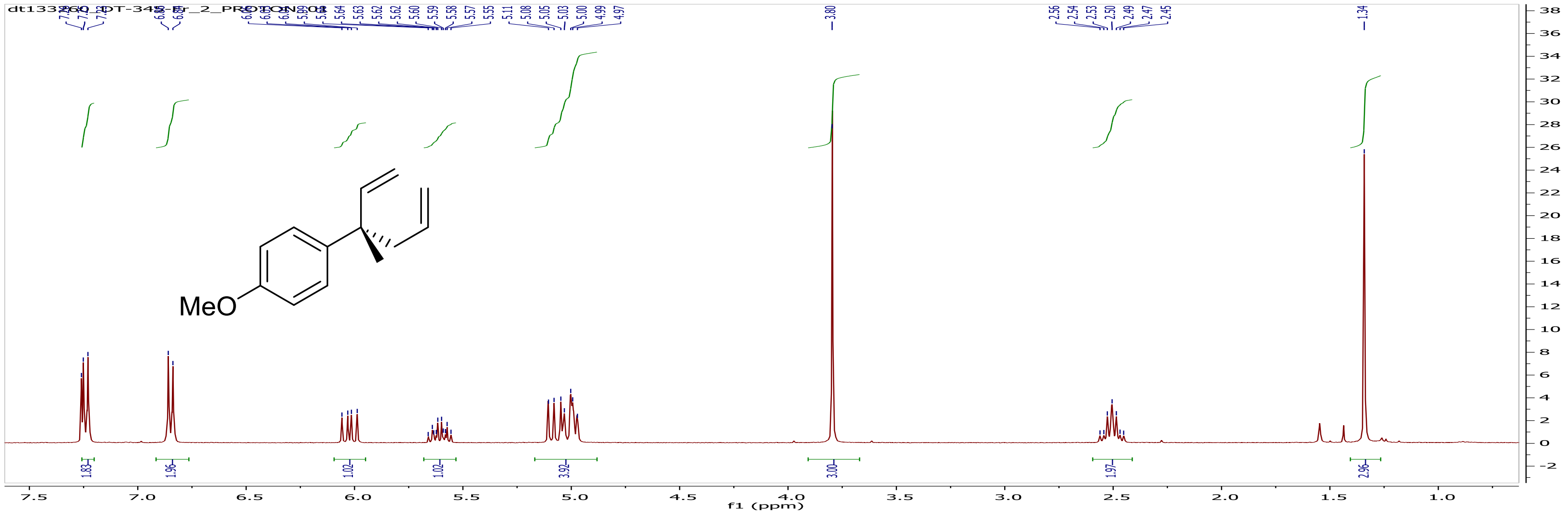

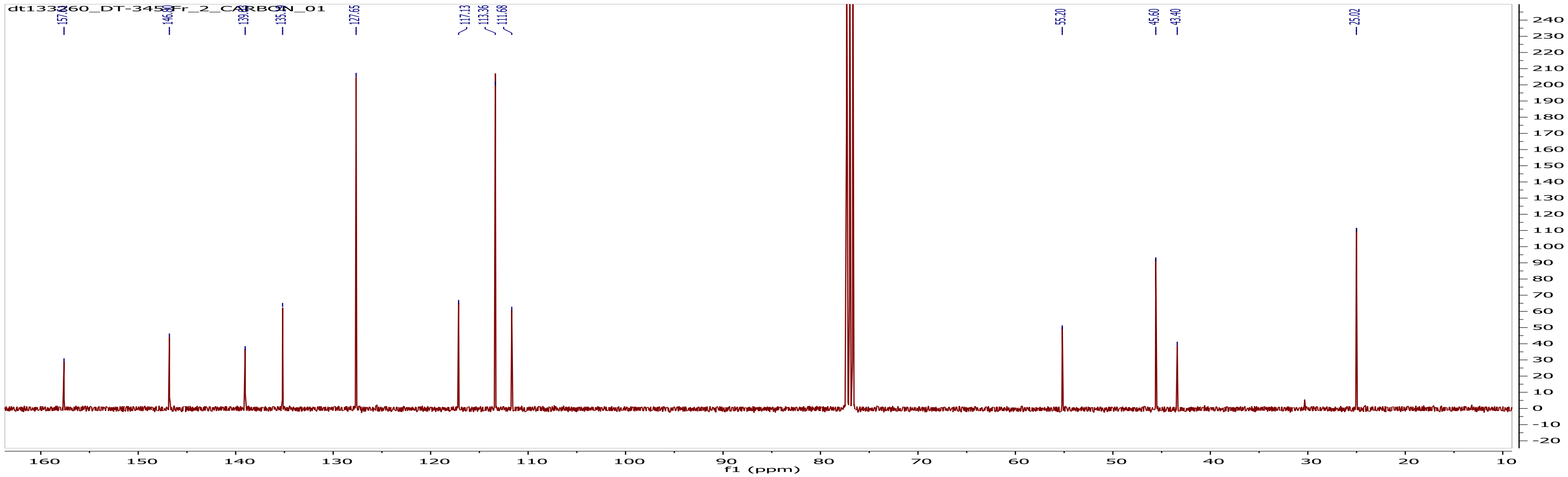

(*S*)-2-(4-methoxyphenyl)propane-1,2-diol (38)

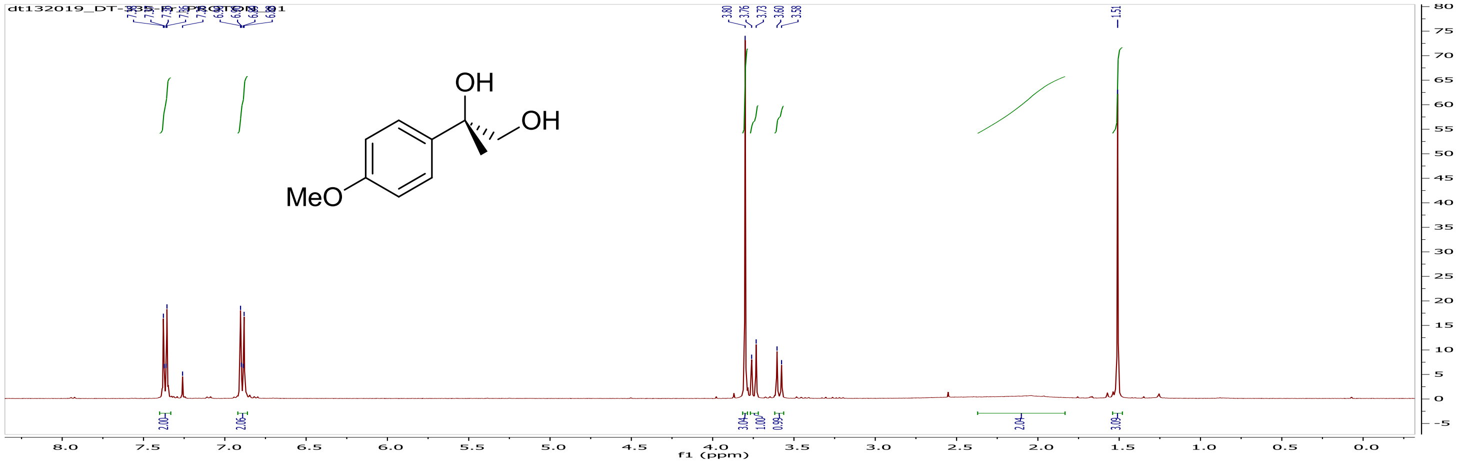

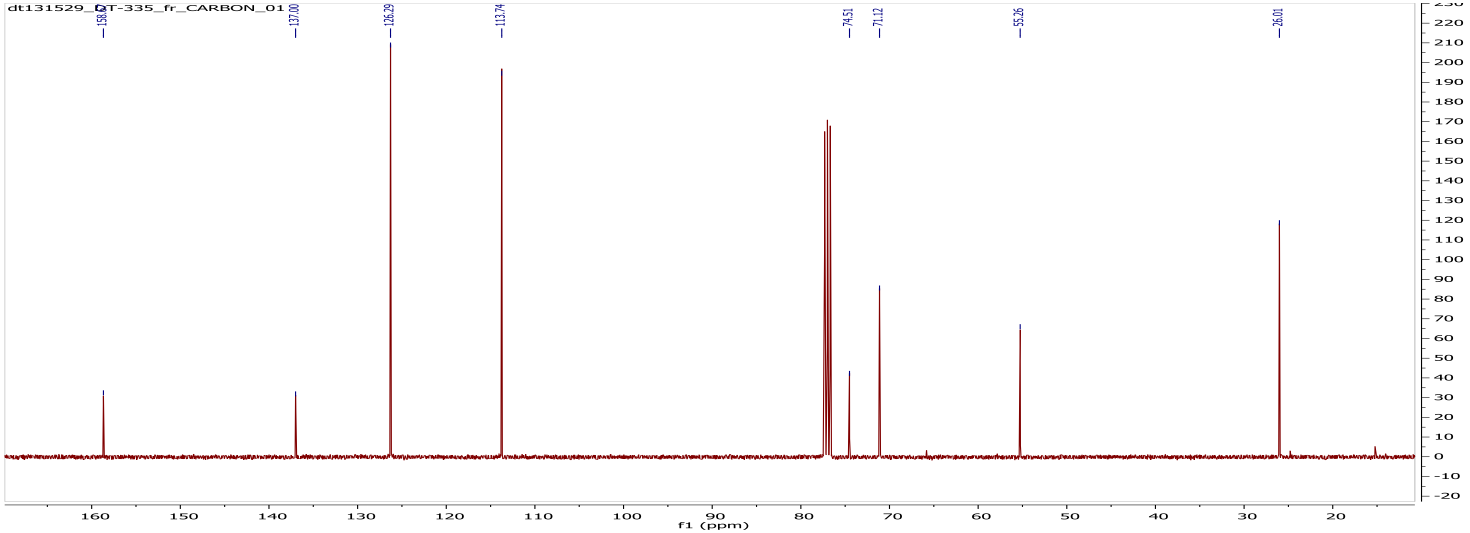

(*S*)-2-(2-(4-methoxyphenyl)propyl)-4,4,5,5-tetramethyl-1,3,2-dioxaborolane (39)

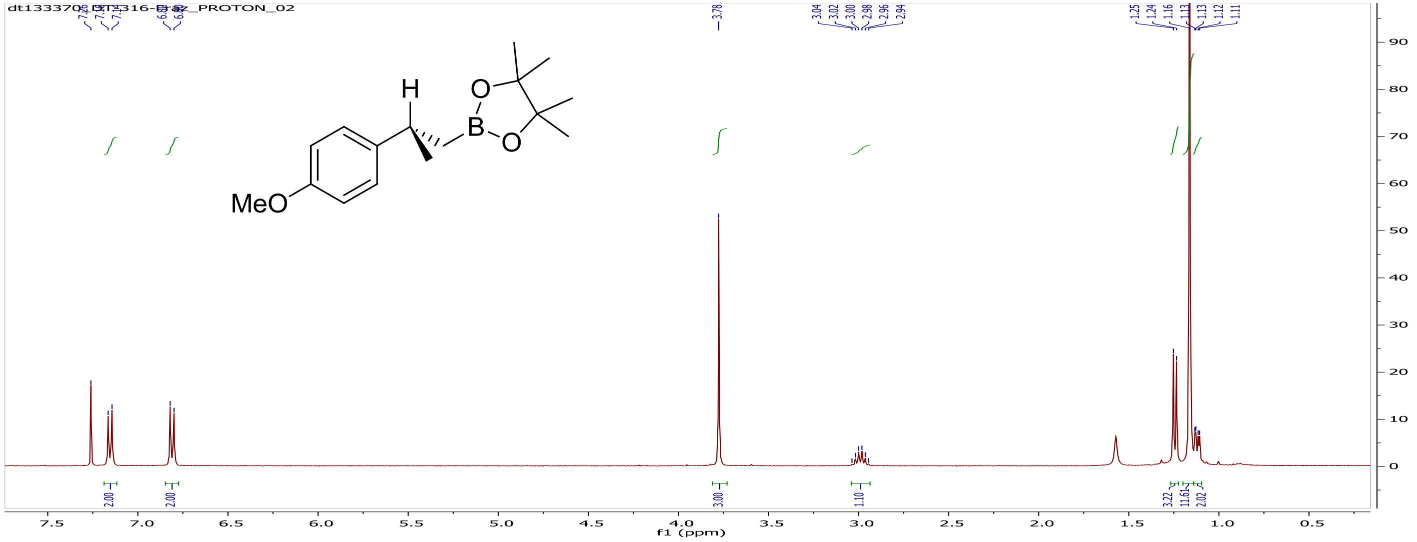

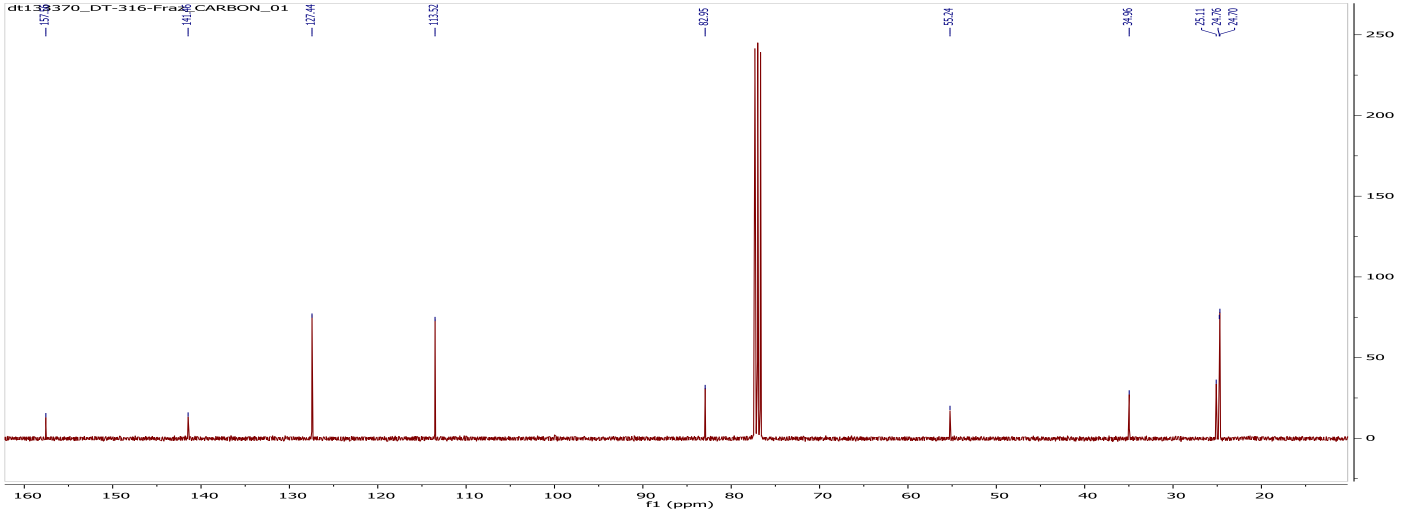

Supplement: Supplementary file 1 [file SC-008-C6SC05338F-s001.pdf]
